# Photodegradable antimicrobial agents: towards structure optimization†

**DOI:** 10.1039/d3ra05554j

**Published:** 2023-10-10

**Authors:** Gabriel Alves Souto de Aquino, Liza Nguyen Van Sang, Romane Valery, Maëlys Lanave, Susana Estopiñá-Durán, Katja S. Håheim, Sabrina Baptista Ferreira, Magne O. Sydnes

**Affiliations:** a Department of Chemistry, Bioscience and Environmental Engineering, Faculty of Science and Technology, University of Stavanger Stavanger NO-4036 Norway magne.o.sydnes@uis.no; b Department of Organic Chemistry, Chemistry Institute, Federal University of Rio de Janeiro Rio de Janeiro 21949-900 Brazil

## Abstract

Antibiotic resistance continues to be an ominous threat facing human health globally and urgent action is required to limit the loss of human life. The pollution of antibiotics into the environment is one of the drivers behind the crisis. With this in mind, we have developed novel photodecomposable antimicrobial agents based on an ethanolamine scaffold, which upon photoirradiation decomposes into two major inactive fragments. Herein we describe our further work on the synthesis of novel ethanolamines with a particular focus on structure activity relationship, resulting in four new active compounds which photodecomposed into inactive fragments.

## Abstract 2

Herein the synthesis of antimicrobial agents that photodecompose after use are described, resulting in a quicker removal of active compounds from the environment. This helps reduce the pollution of antibiotics and assist in the fight against antibiotic resistance.

## Graphical abstract

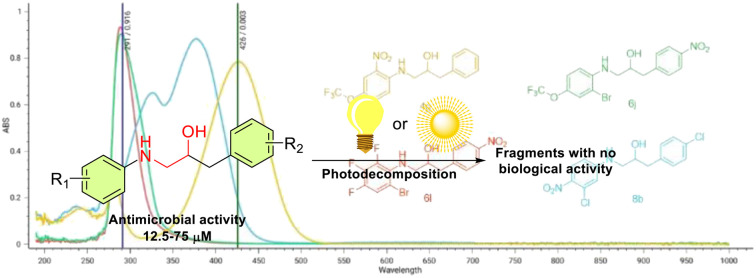

## Introduction

There is an urgent need for new antimicrobial agents to fight the ever-growing problem of antibiotic resistance.^1–3^ In 2019 the death toll as a direct consequence of antibiotic resistance passed 1.27 million people,^4^ and the World Health Organization (WHO) has estimated that within 2050 as many as 10 million people will die annually due to the lack of functioning antibiotics.^5^ WHO warns that new antimicrobial agents are urgently needed to combat the emerging crisis.^1,2^

Knowing that exposure of microbes to small concentrations of antibiotics in the environment is one of several drivers behind antibiotic resistance^6^ is an alarming fact when there are numerous literature reports of antibiotics, other pharmaceuticals and drug metabolites in drinking water, wastewater, ground water, and coastal waters, in addition to traces being found in marine organisms.^7–11^ Therefore it is obvious that in the process of developing new antibiotics there is a need to think alternatively in relation to the lifetime of the active ingredients in the environment after use.

**Fig. 1:**
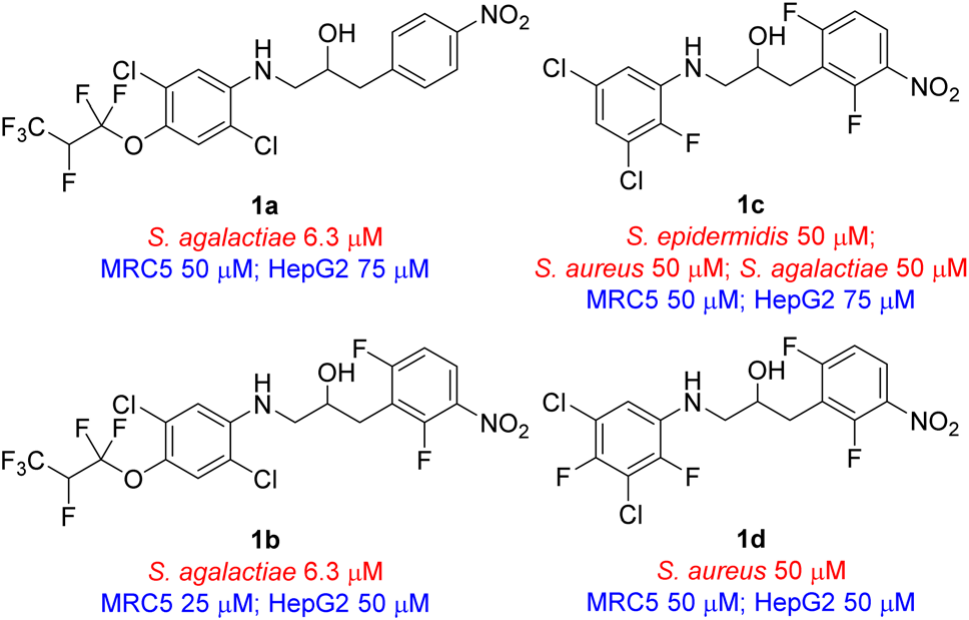
The first generation of active antimicrobial agents that can photodecompose giving products with no antimicrobial activity. Minimum inhibition concentration (MIC) values for microbes are shown in red and cytotoxicity MIC are shown in blue.

Benign by design was a concept that was introduced in 2007 by Kümmerer^12–14^ and it is currently gaining momentum.^15^ With this as a goal we developed a concept of photodegradable antimicrobial agents that are stable in the dark, but decompose into two major inactive fragments upon exposure to light.^16,17^ The concept is based on a built-in photochemical weak point in the compounds that is stable in the dark in a clinical setting, but readily decompose after being secreted by the patient when exposed to light. Our previous work resulted in the generation of four compounds, based on a new scaffold, with antimicrobial activity, *viz.* ethanolamine 1a–1d (Fig. 1). In addition, we have also applied this concept in our preliminary work on making more benign salmon lice treatment.^18^ One aspect with the active compounds developed to date was that they were all heavily halogenated, especially this was the case for the two most active compounds, *viz.* ethanolamines 1a and 1b, possessing a hexafluoropropoxy-substituted aniline unit. In efforts to broaden our understanding of the structure activity relationship (SAR) and reduce the degree of halogenation in compounds with antimicrobial activity we prepared a range of analogues based on the ethanolamine scaffold. Herein we present the preparation and antimicrobial evaluation of 27 new analogues of compounds 1a–1d resulting in four new compounds with interesting antimicrobial activity with three of the four possessing a reduced degree of halogenation in their structure compared to the previously reported compounds (Fig. 1).^16,17^ All four compounds resulted in biologically in-active fragments after photodecomposition.

## Results and discussion

### Synthesis

Inspired by our initial results highlighted in Fig. 1 we aimed to build on these results to further understand the structure activity relationship of our ethanolamine antimicrobial agents while simultaneously reducing the degree of halogenation in the active molecules. In this screening we decided to focus on the substitution pattern on the aromatic rings although enantiomerically pure analogues has the potential to give an enantiomer that is more potent then the other.

The oxirans, *viz.* compound 3, 5, and 7, required for assembling the ethanolamines described herein were prepared according to our previously reported procedure^16,17^ and the anilines utilized were either commercially available or prepared according to literature procedures (see ESI† for details). Initially we utilized lithium perchlorate (5 M solution in Et_2_O) to promote the epoxide ring-opening reaction (Method 1). However, as previously reported^16,17^ (and herein *vide infra*) the yields varied significantly for the formation of the ethanolamines due to the variation of the nucleophilicity of the anilines. In some cases, it was found that the use of lithium bromide in an alcoholic solvent (ethanol or methanol) was more efficient in promoting the nucleophilic attack and the following epoxide ring-opening (Method 2).^19^

**Scheme 1:**
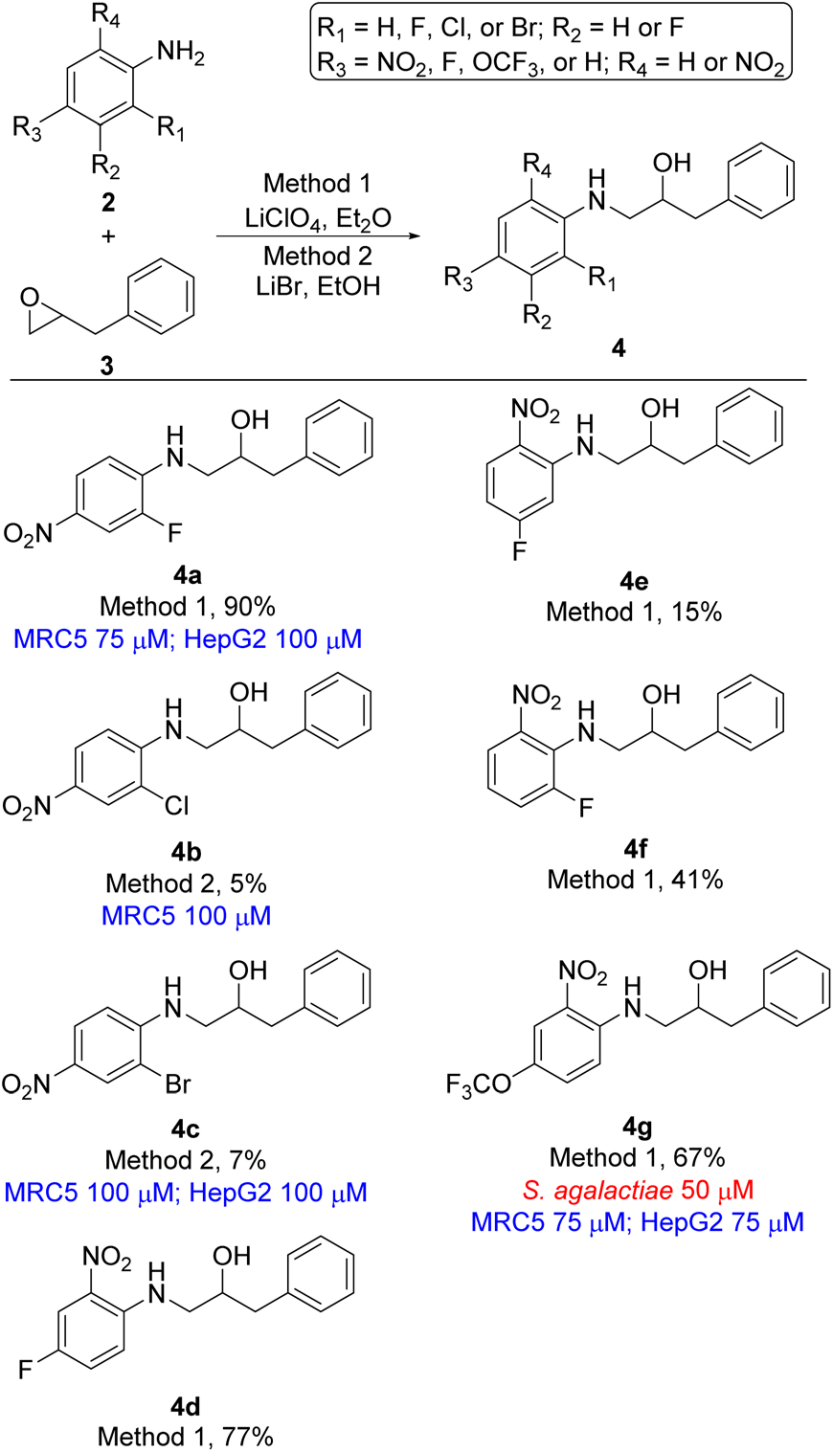
Ethanolamine analogues based on 2-benyloxirane (3). Minimum inhibition concentration (MIC) values for microbes are shown in red and cytotoxicity MIC are shown in blue.

In total seven analogues of ethanolamine 4 were prepared using lithium promoted aniline ring-opening of oxiran 3 with substituted nitro anilines 2 (Scheme 1). The desired products were formed in a broad range of yields reflecting the nucleophilicity of the aniline. However, since we in the first instance were mostly interested in generating new ethanolamines the yield of formation was of secondary interest if sufficient material for biological evaluation was obtained. Yields for the formation of compounds 4b, 4c, and 4e are good examples of that point. Ethanolamine 4b and 4c, which are chloro and bromo analogues of compound 4a, respectively, could not be formed using lithium perchlorate (Method 1) and only gave the desired product in minute yields (compound 4b 5%, compound 4c 7%) when treated with lithium bromide (Method 2). The increased electronegativity switching from fluorine to chlorine and bromine substitution in 2-position of the aniline changes the nucleophilicity of the aniline dramatically (4a*vs.*4b and 4c). For ethanolamines 4d–4f the nucleophilicity of the aniline is altered depending on the position of the fluorine. As an overall assessment it appears that *para*- or *ortho*-fluoro nitroanilines are sufficiently nucleophilic to undergo the epoxide ring-opening for the evaluated substrates, while *meta*-fluoro nitroanilines are poorer nucleophiles under our reaction conditions.

**Scheme 2:**
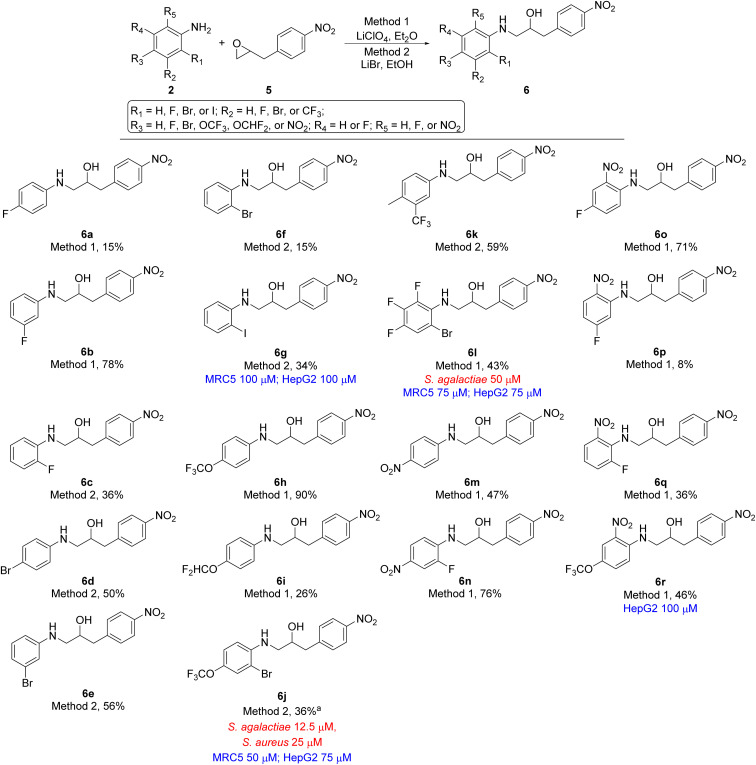
Ethanolamine analogues based on 2-(4-nitrobenyl)oxirane (5). MIC values for microbes are shown in red and cytotoxicity are shown in blue. ^*a*^Using methanol as solvent instead of ethanol give 6j in 40% yield.

2-(4-Nitrobenzyl)oxirane 5 was treated with 18 differently substituted anilines using either lithium perchlorate (Method 1) or lithium bromide (Method 2) as promotor for the ring-opening. This resulted in formation of ethanolamines 6a–6r in yields ranging from 8 to 90% (Scheme 2). Also, in this series of compounds the nucleophilic nature of the aniline strongly influenced the reactivity of the aniline and consequently the yield of the product. However, the clear trends seen for compounds 4a–4g (Scheme 1) were not present in this series of compounds.

**Scheme 3:**
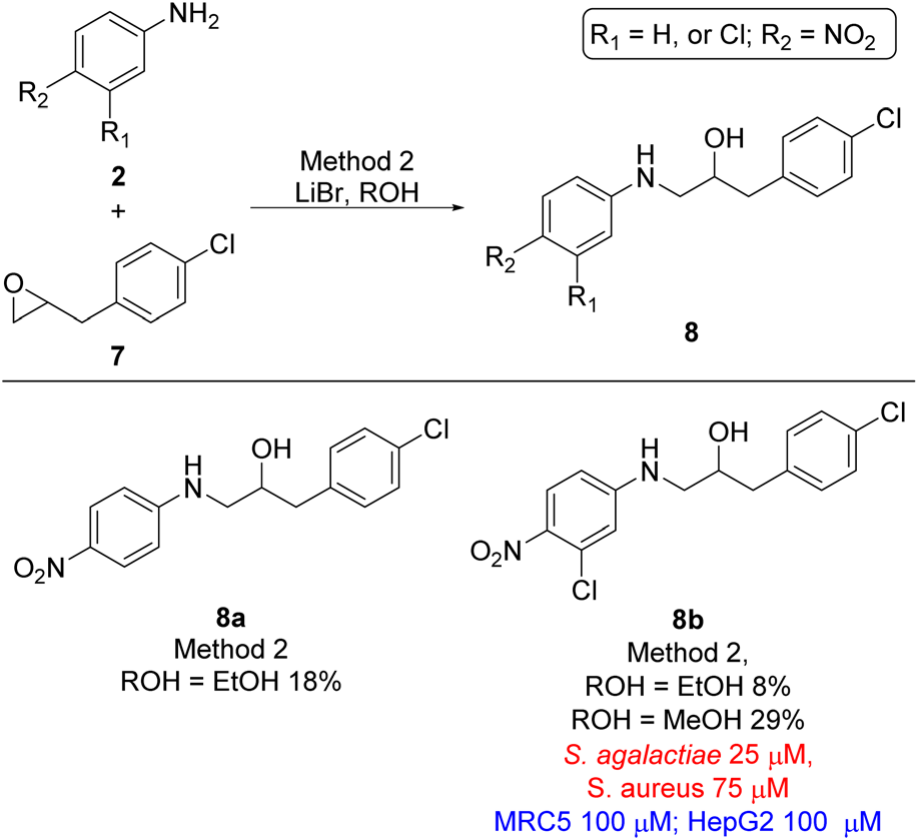
Ethanolamine analogues based on 2-(4-chlorobenyl)oxirane (7). MIC values for microbes are shown in red and cytotoxicity MIC are shown in blue.

Finally, the two last analogues, *viz.* ethanolamine 8a and 8b, were prepared by treating oxirane 7 with lithium bromide in ethanol resulting in formation of target compounds 8a and 8b in 18% and 8% yield, respectively (Scheme 3). Interestingly the yield of ethanolamine 8b could be increased from 8% to 29% when methanol was used as solvent instead of ethanol. Despite the broad variation in yields for the formation of the target ethanolamines 4a–4g, 6a–6r, 8a, and 8b from the corresponding oxiranes and anilines the synthetic strategy did provide sufficient material for biological evaluation.

### Biological evaluation

With the desired ethanolamine derivatives 4a–4g, 6a–6r, 8a, and 8b in hand we subjected them to a bacteria inhibition assay in liquid media against selected Gram-positive and Gram-negative bacteria, including *Escherichia faecalis*, *E. coli*, *Pseudomonas aeruginosa*, *Staphylococcus aureus*, and *S. agalactiae* following our previously reported method (Tables 1 and S1 in ESI†).^20^ Compounds 6j and 8b showed promising antimicrobial activity with a minimum inhibitory concentration (MIC) value of 12.5 μM and 25 μM, respectively, against *S. agalactiae* (Table 1 and Schemes 2 and 3). These two compounds also showed activity towards *S. aureus*, where compound 6j displayed a MIC of 25 μM and compound 8b showed considerable weaker activity with a MIC of 75 μM. *S. aureus* is high up on WHO's list of drug resistant bacteria that are in urgent need of new antibiotics.^21^ This highlights the fact that compounds 6j and 8b together with ethanolamine 1a and 1b from our previous work^16,17^ are interesting lead compounds for further optimalization. In addition, compound 4g and 6l (Table 1 and Schemes 1 and 2) also showed interesting activity against *S. agalactiae* with MIC of 50 μM.

**Table 1:** Minimum inhibitory concentration (MIC) in μM against Gram-positive bacteria and toxicity tests against MRC5 and HepG2 cell lines for compounds showing activity. The activity was screened at the following concentrations: 100, 75, 50, 25, 12.5, 6.3, 3.1, and 1.6 μM^a^

Compound	MIC		Tox	
	*S. agalactiae*	*S. aureus*	MRC5	HepG2
4a	I	I	75	100
4b	I	I	100	I
4c	I	I	100	100
4g	50	I	75	75
6g	I	I	100	100
6j	12.5	25	50	75
6l	50	I	75	75
6r	I	I	I	100
8b	25	75	100	100

a I = inactive at the tested concentrations.

The remaining compounds did not show antimicrobial activity (MIC > 100 μM). The fact that in particular ethanolamine 6j displayed promising activity towards *S. agalactiae* shows that it is possible to lower the degree of halogenation in the molecule compared with compound 1a and 1b (Fig. 1) while simultaneously keeping a significant proportion of the antimicrobial activity towards *S. agalactiae*. The activity for ethanolamines 4g and 6l were equivalent with the activity shown by compound 1c, however, compounds 4g and 6l shows selectivity towards *S. agalactiae*, which was not the case for 1c (Fig. 1). All four compounds showed cytotoxicity against MRC5 and HepG2 cells, but at higher concentrations then their antimicrobial activity, which indicates that it is a possible to control toxicity of the compounds at concentrations relevant for antimicrobial activity. Compounds 4a–4c (Table 1 and Scheme 1), 6r and 6g (Table 1 and Scheme 2) did not show antimicrobial activity (MIC > 100 μM) against the five microbes tested in this study, but these compounds possessed low cytotoxicity towards MRC5 and/or HepG2 (see Table 1 and Schemes 1 and 2 for details).

### Photodegradation

**Fig. 2:**
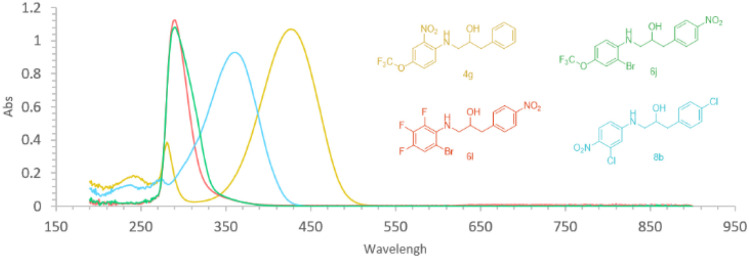
UV-vis spectroscopic data for compounds 4g [yellow; UV-vis (EtOH) *λ*_max_ 427 nm (*ε* 5883 M^−1^ cm^−1^)], 6j [green; UV-vis (EtOH) *λ*_max_ 290 nm (*ε* 20 045 M^−1^ cm^−1^)], 6l [red; UV-vis (EtOH) *λ*_max_ 290 nm (*ε* 18 301 M^−1^ cm^−1^)]), and 8b (blue; UV-vis (EtOH) *λ*_max_ 361 nm (*ε* 21 151 M^−1^ cm^−1^)].

A requirement for compounds to undergo photodegradation in nature is that they absorb light with wavelengths of *ca.* 300 nm and higher. This was the case for all the ethanolamines with antimicrobial activity, namely compounds 4g (*λ*_max_ 428 nm), 6j (*λ*_max_ 303 nm), 6l (*λ*_max_ 311 nm), and 8b (*λ*_max_ 316 nm) (Fig. 2). The four active compounds in acetonitrile/water (7 : 3 v/v) with pH 7.0 were subjected to photodegradation by irradiation with a medium pressure mercury lamp according to our previously reported method.^17^ Photoirradiation for 24 hours resulted in full decomposition of the four ethanolamines that possessed antimicrobial activity, *viz.* compound 4g, 6j, 6l, and 8b, resulting in decomposition by a photo-*retro*-aldol-type reaction giving fragments which are in line with products previously reported by us derived from photodecomposition of compound 1a.^17^ The resulting product mixtures were subjected to antimicrobial testing against the same five microbes as their parent compounds in addition to cytotoxicity testing (MRC5 and HepG2). All mixtures derived from photodecomposition of the active compounds possessed no activity (MIC >100 μM) in the biological tests.

### Structure activity relationship

**Fig. 3:**
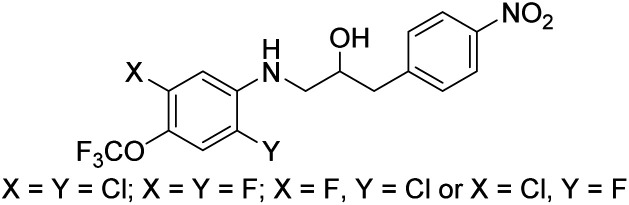
Preliminary structure activity relationship.

Based on the biological activity (both active and inactive compounds) obtained for the ethanolamines prepared in this study and our previously reported work^16,17^ gives room for a few preliminary conclusions. Results from compound 4g and 6j shows that it is possible to maintain a significant activity even with a truncated ether functionality compared with ethanolamines 1a and 1b. These results point towards a trifluoromethoxy group *para* to the amine functionality in the aniline moiety of the molecule is sufficient for activity towards *S. agalactiae* (Fig. 3). Furthermore, data from this study and our previous work^16,17^ also points towards the need for halogens *ortho* and *meta* to the amine functionality as depicted in Fig. 3. This conclusion is strongly evident from results obtained on ethanolamines **1a–1d** and further confirmed by results obtained in this study. From our previous work we also know that having the nitro group *para* to the propan-2-ol moiety, namely compound 1a, results in clear cut fragmentation when exposed to light.^17^

The biological data for compounds 8a and 8b shows that the aniline moiety in the ethanolamines needs a higher degree of substitution then just the nitro group as is the case for 8a. Dramatic improvement in activity is seen when chlorine is placed in the *meta-*position with respect to the aniline N-atom, however, further results are required to get a better understanding of the influence of substitution pattern on the biological activity for this series of ethanolamines.

## Experimental

### General procedure for Lewis acid (LiClO_4_) promoted epoxide ring opening (Method 1)

Anhydrous lithium perchlorate was dried under vacuum for 1 h and dissolved in anhydrous diethyl ether to give a 5 M solution. The appropriate aniline (1.0 equiv., 0.2 M) and oxirane (1.0–1.5 equiv., ≈0.2 M) was added, and the resulting reaction mixture was refluxed under an atm. of argon for 12 h-5 days. Water was then added to the reaction mixture and the product was extracted with DCM (3 × 20 mL). The combined organic phases were evaporated on Celite and subjected to silica-gel flash chromatography and concentration of the relevant fractions gave the title compound. Recrystallization was performed when necessary.

### General procedure for Lewis acid (LiBr) promoted epoxide ring opening (Method 2)

Aniline (1.0 equiv.), oxirane (1.0–1.5 equiv.), lithium bromide (0.5 equiv.), and ethanol or methanol (0.5 mL) was stirred at reflux for 24–72 h depending on the aniline. Water was added to the reaction mixture and the water phase was extracted with DCM (3 × 20 mL). The combined organic phases were evaporated on Celite and subjected to silica-gel flash chromatography and the concentration of the relevant fractions yielded the products. Recrystallization was performed when necessary.

### General procedure for photodecomposition of 4g, 6j, 6l, and 8b

A solution of ethanolamine (≈0.10 mmol) in acetonitrile was added to a photochemical reactor containing water with pH 7 to a concentration of ≈0.7 mM and a total volume of 75 mL (acetonitrile/water 7 : 3). The reaction vessel was left open to air and photolyzed with a 6 W low pressure mercury-vapor lamp (mainly 254 nm irradiation) for 24 h. After completion, the reaction mixture was transferred to a separatory funnel, saturated with NaCl, and extracted with EtOAc (3 × 50 mL). The combined organic layers were dried (MgSO_4_), filtered, and concentrated *in vacuo* on a rotary evaporator to yield a residue which was subjected for antimicrobial and cytotoxicity testing.

### Growth inhibition assay

Ethanolamines 4a–4g, 6a–6r, 8a, and 8b were tested for their bacteria growth inhibition in liquid media against *Staphylococcus aureus* (ATCC 25923), *Escherichia coli* (ATCC 259233), *Enterococcus faecalis* (ATCC 29122), *Pseudomonas aeruginosa* (ATCC 27853) and *Streptococcus agalactiae* (ATCC 12386) at concentrations ranging from 1.56 to 100 μM (the following test concentrations were used 100 μM, 75 μM, 50 μM, 25 μM, 12.5 μM, 6.25 μM, 3.1 μM, and 1.6 μM) (see ESI† for details).

### Cytotoxicity assay

The antiproliferative activities of compounds 4a–4g, 6a–6r, 8a, and 8b were evaluated against the melanoma cell line, the hepatocellular carcinoma cell line HepG2 (ATCC, HB-8065™), and the non-malignant lung fibroblast cell line MRC5 (ATCC, CCL-171™) in an MTS *in vitro* cell proliferation assay. The following concentrations were used in the assay 100 μM, 75 μM, 50 μM, 25 μM, 12.5 μM, 6.25 μM, 3.1 μM, and 1.6 μM (see ESI† for details).

## Conclusions

In conclusion, four compounds based on our novel ethanolamine scaffold, *viz.*4g, 6j, 6l, and 8b, possess antimicrobial activity towards *S. agalactiae.* Of the four active compounds particularly ethanolamine 6j is of interest to further follow up displaying a MIC of 12.5 μM. Compound 6j also displayed interesting activity (25 μM) towards *S. aureus*, a bacterium that WHO has on their list of bacteria with an urgent need of new treatment for. All compounds with antimicrobial activity also decomposed upon exposure to light into fragments that possess no antimicrobial activity (MIC > 100 μM) and no toxicity (MIC > 100 μM). The biological results also showed that the 1,1,2,3,3,3-hexafluoropropoxy functionality within our two most active compounds to date, namely ethanolamine 1a and 1b, can be truncated to a trifluoromethoxy with only a minor loss of antimicrobial activity. Furthermore, the biological results in this study also quite clearly shows that the aniline moiety needs to be halogenated (fluorine and/or chlorine, or a combination of the two) in 2- and 5-position in relation to the amine functionality in the aniline. None of the active compounds were active towards Gram-negative bacteria, however, work by Hergenrother and co-workers outlines strategies that can be applied to expand their potential to include Gram-negative bacteria as well.^22^ Work directed towards increasing the biological activity and obtaining compounds with activity against Gram-negative bacteria based on the ethanolamine scaffold are ongoing in these laboratories. Further work also includes the synthesis of enantiomerically pure ethanolamines (compounds 1a, 1b, 4g, 6j, 6l, and 8b) by using the relevant chiral oxiran as starting material with the aim to obtain enantiomers that has enhanced activity. In addition, we have also initiated further photodecomposition studies using LED-NMR with focused wavelengths to shade more light on the photodecomposition process. Results from these studies will be reported in due course.

## Declaration

We encourage the citation of primary research over review articles, where appropriate, in order to give credit to those who first reported a finding. Find out more about our commitments to the principles of San Francisco Declaration on Research Assessment (DORA).

## Author contributions

Conceptualization MOS; data curation GASA, LNVS, RV, ML, SED, KSH; formal analysis GASA, LNVS, RV, ML, SED, KSH; funding acquisition MOS; methodology MOS, GASA, LNVS, SED, KSH; project administration MOS; resources MOS, SBF; supervision MOS, SED, KSH; Validation MOS; visualization MOS, LNVS; writing—original draft MOS; writing—review & editing MOS, LNVS, KSH, SED, SBF.

## Conflicts of interest

There are no conflicts to declare.

## Supplementary Material

RA-013-D3RA05554J-s001

## References

[cit1] Miethke M. (2021). et al.. Nat. Rev. Chem..

[cit2] WHO , WHO publishes list of bacteria for which new antibiotics are urgently needed, https://www.who.int/news/item/27-02-2017-who-publishes-list-of-bacteria-for-which-new-antibiotics-are-urgently-needed, accessed 13 April 2023

[cit3] Laxminarayan R. (2013). et al.. Lancet Infect. Dis..

[cit4] Murray C. J. L. (2022). et al.. Lancet.

[cit5] May M. (2021). Nat. Med..

[cit6] Hanna N., Tamhankar A. J., Lundborg C. S. (2023). Lancet Planet. Health.

[cit7] Danner M. C., Robertson A., Behrends V., Reiss J. (2019). Sci. Total Environ..

[cit8] Khetan S. K., Collins T. J. (2007). Chem. Rev..

[cit9] Kümmerer K. (2009). Chemosphere.

[cit10] Sanseverino I. , Cuenca A. N., Loos R., Marinov D. and Lettieri T., JRC Technical Reports, Stat of the Art on the Contribution of Water to Antimicrobial Resistance, 2018, https://publications.jrc.ec.europa.eu/repository/handle/JRC114775, accessed 13 April 2023

[cit11] Carvalho I. T., Santos L. (2016). Environ. Int..

[cit12] Kümmerer K. (2007). Green Chem..

[cit13] Kümmerer K. (2019). Sustainable Chem. Pharm..

[cit14] Moermond C. T. A., Puhlmann N., Brown R., Owen S. F., Ryan J., Snape J., Venhuis B. J., Kümmerer K. (2022). Environ. Sci. Technol. Lett..

[cit15] Sydnes M. O. (2023). Curr. Green Chem..

[cit16] Eikemo V., Sydnes L. K., Sydnes M. O. (2021). RSC Adv..

[cit17] Eikemo V., Holmelid B., Sydnes L. K., Sydnes M. O. (2022). J. Org. Chem..

[cit18] Sydnes M. O., Eikemo V., Espedal P. G., Sydnes L. K., Nilsen F. (2023). Aquaculture.

[cit19] Yang Z., Sheng X., Yan M., Ramström O. (2019). Mol. Catal..

[cit20] Håheim K. S., Albrigtsen M., Helland K., Andersen J. H., Sydnes M. O. (2023). Heterocycles.

[cit21] Willyard C. (2017). Nature.

[cit22] Richter M. F., Drown B. S., Riley A. P., Garcia A., Shirai T., Svec R. L., Hergenrother P. J. (2017). Nature.

