# Supplementary material for: Photodegradable antimicrobial agents: towards structure optimization
Source: RSC Adv. 2023 Oct 10;13(42):29729–34. doi: 10.1039/d3ra05554j (PMC10563738; doi:10.1039/d3ra05554j)
Supplement: RA-013-D3RA05554J-s001 [file RA-013-D3RA05554J-s001.pdf]

Electronic Supplementary Material (ESI) for RSC Advances.
This journal is © The Royal Society of Chemistry 2023

Supplementary information

Photodegradable Antimicrobial Agents: Towards Structure Optimization

Gabriel Alves Souto de Aquino,[ab] Liza Nguyen Van Sang,[a] Romane Valery,[a] Maëlys Lanave,[a] Susana Estopiñá-Durán,[a] Katja S. Håheim,[a] Sabrina Baptista Ferreira[b] and Magne O. Sydnes*[a]

[a]Department of Chemistry, Bioscience and Environmental Engineering, Faculty of Science and Technology, University of Stavanger, Stavanger NO-4036, Norway.

[b]Department of Organic Chemistry, Chemistry Institute, Federal University of Rio de Janeiro, Rio de Janeiro 21949-900, Brazil.

Corresponding author e-mail: magne.o.sydnes@uis.no

Table S1	2
General experimental information	3
General procedure for synthesis of ethanolamine using lithium perchlorate - Method 1	4
General procedure for synthesis of ethanolamine using lithium bromide - Method 2	4
Preparation of ethanolamines **4**, **6**, and **8**	4
Procedures for preparing precursors for ethanoalamine synthesis	14
Growth inhibition assay	18
Cytotoxicity assay	19
References	20
NMR spectra	21

Table S1. Minimum inhibitory concentration (MIC) in μM against Gram-positive and Gram-negative bacteria and toxicity tests against MRC5 and HepG2 cell lines. The activity was screened at the following concentrations: 100, 75, 50, 25, 12.5, 6.3, 3.1, and 1.6 μM.[a]

	MIC					Tox	
Comp.	*S. agalactiae*	*S. aureus*	*E. faecalis*	*E. coli*	*P. aeruginosa*	MRC5	HepG2
**4a**	I	I	I	I	I	75	100
**4b**	I	I	I	I	I	100	I
**4c**	I	I	I	I	I	100	100
**4d**	I	I	I	I	I	I	I
**4e**	I	I	I	I	I	I	I
**4f**	I	I	I	I	I	I	I
**4g**	50	I	I	I	I	75	75
**6a**	I	I	I	I	I	I	I
**6b**	I	I	I	I	I	I	I
**6c**	I	I	I	I	I	I	I
**6d**	I	I	I	I	I	I	I
**6e**	I	I	I	I	I	I	I
**6f**	I	I	I	I	I	I	I
**6g**	I	I	I	I	I	100	100
**6h**	I	I	I	I	I	I	I
**6i**	I	I	I	I	I	I	I
**6j**	12.5	25	I	I	I	50	75
**6k**	I	I	I	I	I	I	I
**6l**	50	I	I	I	I	75	75
**6m**	I	I	I	I	I	I	I
**6n**	I	I	I	I	I	I	I
**6o**	I	I	I	I	I	I	I
**6p**	I	I	I	I	I	I	I
**6q**	I	I	I	I	I	I	I
**6r**	I	I	I	I	I	I	100
**8a**	I	I	I	I	I	I	I
**8b**	25	75	I	I	I	100	100

[a]I = inactive at the tested concentrations.

Experimental Section

General Information

All chemicals were purchased from VWR Avantor, Merck or Enamine Ltd and used without further purification. Anhydrous THF was prepared by distillation from sodium benzophenone ketyl over a nitrogen atmosphere and stored over 4 Å MS.

IR spectra were recorded on an Agilent Cary 630 FT-IR spectrophotometer equipped with an attenuated total reflectance (ATR) attachment. Samples were analyzed neat, and the absorption frequencies are given in wave numbers (cm^{-1}) and are assigned as broad (br) when relevant.

UV-vis spectra were obtained on an Agilent 8453 single-beam UV-vis spectrophotometer with a deuterium discharge lamp for the UV range and a tungsten lamp for the visible wavelength range. Samples were analyzed in an Agilent open-top UV quartz cell (10 mm, 3.0 mL) with ethanol as solvent. Wavelengths are reported in nm and extinction coefficients in $M^{-1}cm^{-1}$.

NMR spectra were recorded on a Bruker Ascend™ 400 spectrometer (400.13 MHz for ^{1}H, 100.61 MHz for ^{13}C, and 376.46 MHz for ^{19}F). Coupling constants (*J*) are given in Hz and the multiplicity is reported as singlet (s), doublet (d), triplet (t), quartet (q), multiplet (m), double doublet (dd), doublet of doublet of doublet (ddd), and broad singlet (bs). The chemical shift values are reported upfield to downfield in ppm and calibration is done using the residual solvent signals for chloroform-*d* (^{1}H 7.26 ppm; ^{13}C 77.16 ppm), and acetonitrile-d_3 (^{1}H 1.94 ppm; ^{13}C 1.32 ppm). Calibration for ^{19}F NMR is done using α,α,α-trifluorotoluene as internal standard in chloroform-*d* (-62.61 ppm) and acetonitrile-d_3 (-63.10 ppm).[1]

Thin-layer chromatography (TLC) was carried out with silica gel (60 F_{254}) on aluminium sheets with solvent systems consisting of various mixtures of petroleum ether, ethyl acetate, and methanol. Visualization was achieved with either exposure to UV light (254 and/or 365 nm) or sulfuric vanillin stain. Flash chromatography was performed with a hand pump and 230-400 mesh silica gel.

HRMS data has proved to be very difficult to obtain for the ethanolamines due to very poor ionization under conditions and instrumentation available for us. However, the spectroscopical data obtained for our final products are in full accord with their assigned structures.

General procedure for synthesis of ethanolamine using lithium perchlorate - Method 1

Lithium perchlorate was dried under vacuum for 1 h and dissolved in anhydrous diethyl ether to a 5 M solution. The appropriate aniline (1.0 equiv.), (0.2 M) and epoxide (1.0 - 1.5 equiv.), (~ 0.2 M) was added and the reaction mixture was refluxed under an argon atmosphere for 12-48 h depending to the aniline. Water was added to the reaction mixture, then extracted with DCM (3 x 20 mL). The combined organic phases were evaporated on celite and subjected to silica-gel flash chromatography and the concentration of the relevant fractions yielded the products. Recrystallization was performed when necessary.

General procedure for synthesis of ethanolamine using lithium bromide - Method 2

The appropriate aniline (1.0 equiv.), epoxide (1.0 - 1.5 equiv.), LiBr (0.5 equiv.), and ethanol (2 mL) was stirred at ambient temperature for 24-72 h depending on the aniline. Water was added to the reaction mixture and the aqueous phase was extracted with DCM (3 x 20 mL). The combined organic phases were evaporated on celite and subjected to silica-gel flash chromatography and the concentration of the relevant fractions yielded the products. Recrystallization was performed when necessary.

Preparation of ethanolamines 4, 6, and 8

Synthesis of 1-((2-fluoro-4-nitrophenyl)amino)-3-phenylpropan-2-ol (4a)

2-fluoro-4-nitroaniline (65 mg, 0.42 mmol) and 2-benzyloxirane (84 mg, 0.63 mmol) was reacted according to Method 1 for 48 h to yield compound **4a** (109 mg, 90%) (silica gel chromatography DCM; R_f = 0.20 in DCM) as a dark yellow oil.

IR (neat): ν_{max} 3470, 3293, 3087, 3025, 2923, 1607, 1550, 1492, 1451, 1284, 1258, 1190, 1085, 1066, 735, 698; UV-vis (EtOH): λ_{max} 386 nm; ^{1}H NMR (400 MHz, CD_3CN): δ 7.49-7.45 (m, 2H), 7.33-7.20 (m, 5H), 7.15-7.10 (m, 1H), 4.96 (bs, 1H), 4.03-3.98 (m, 1H), 3.30 (ddd, *J* = 13.3 Hz, 6.4 Hz, 3.7 Hz, 1H), 3.16-3.10 (m, 2H), 2.85 (dd, *J* = 13.7 Hz, 5.4 Hz, 1H), 2.77 (dd, *J* = 13.7 Hz, 7.7 Hz, 1H); ^{13}C NMR (100 MHz, CD_3CN): δ 155.6 (d, *J* = 248.7 Hz), 146.2, 139.8, 138.8 (d, *J* = 13.4 Hz), 130.4, 129.3, 127.2, 115.5, (d, *J* = 21.8 Hz), 112.6, (d, *J* = 8.6 Hz), 107.4 (d, *J* = 6.1 Hz), 71.5, 49.3, 42.0; ^{19}F NMR (376 MHz, CD_3CN): δ -127.3.

Synthesis of 1-((2-chloro-4-nitrophenyl)amino)-3-phenylpropan-2-ol (4b)

2-Chloro-4-nitroaniline (100 mg, 0.58 mmol) and 2-benzyloxirane (86 mg, 0.64 mmol) was reacted according to Method 2 for 65 h to yield compound **4b** (9 mg, 5%) (silica gel chromatography pet. ether/DCM 3:7, v/v → chloroform/methanol, 99:1 v/v; R_f = 0.20 in pet. ether/DCM 3:7, v/v) as a yellow oil.

IR (neat): ν_{max} 3394, 3178, 3025, 2918, 1586, 1527, 1491, 1452, 1317, 1275, 1121, 1082, 744, 699; UV-vis (EtOH): λ_{max} 298 nm; ^{1}H NMR (400 MHz, CD_3CN): δ 8.19 (d, J = 2.6 Hz, 1H), 8.02 (ddd, J = 9.3 Hz, 2.6 Hz, 0.6 Hz, 1H), 7.33-7.23 (m, 5H), 6.70 (d, J = 9.2 Hz, 1H), 5.79 (bs, 1H), 4.07-4.00 (m, 1H), 3.39 (ddd, J = 13.5 Hz, 6.2 Hz, 3.7 Hz, 1H), 3.26-3.20 (m, 2H), 2.85 (dd, J = 13.8 Hz, 5.4 Hz, 1H), 2.77 (dd, J = 13.6 Hz, 7.7 Hz, 1H); ^{13}C NMR (100 MHz, CD_3CN): δ 150.6, 139.6, 137.9, 130.4, 129.3, 127.3, 126.2, 125.7, 110.5, 71.4, 49.3, 42.1, one signal is obscured or overlapping.

Synthesis of 1-((2-Bromo-4-nitrophenyl)amino)-3-phenylpropan-2-ol (4c)

2-Benzyloxirane (50 mg, 0.372 mmol) and 2-bromo-4-nitrianiline (81 mg, 0.372 mmol) was reacted according to Method 2 for 6 days to yield compound **4c** (9 mg, 7%) (silica gel chromatography pet. ether/DCM, 3:7, v/v; R_f = 0.17 in pet. ether/DCM, 3:7, v/v) as a yellow oil.

IR (neat): ν_{max} 3509, 3393, 2922, 1709, 1495, 700 cm^{-1}; UV-vis (EtOH): λ_{max} 332 nm; ^{1}H NMR (400 MHz, CD_3CN): δ 8.33 (d, J = 2.6 Hz, 1H), 8.05 (dd, J = 8.5, 2.8 Hz, 1H), 7.37-7.17 (m, 5H), 6.67 (d, J = 9.2 Hz, 1H), 5.73 (bs), 4.05-3.96 (m, 1H), 3.39 (ddd, J = 13.4, 6.1, 3.7 Hz, 1H), 3.24-3.21 (m, 2H), 2.85 (dd, J = 13.7, 5.5 Hz, 1H), 2.78 (dd, J = 13.7, 7.6 Hz, 1H); ^{13}C NMR (100 MHz, CD_3CN): δ 151.5, 139.5, 138.2, 130.4, 129.4, 129.3, 127.3, 126.2, 110.4, 108.1, 71.3, 49.4, 42.1.

Synthesis of 1-((4-fluoro-2-nitrophenyl)amino)-3-phenylpropan-2-ol (4d)

4-fluoro-2-nitroaniline (50 mg, 0.32 mmol) and 2-benzyloxirane (65 mg, 0.48 mmol) was reacted according to Method 1 for 48 h to yield compound **4d** (69 mg, 77%) (silica gel chromatography pet. ether/DCM 1:9, v/v → pet. ether/EtOAc, 7:3 v/v; R_f = 0.40 in pet. ether/EtOAc, 7:3 v/v) as a red oil.

IR (neat): ν_{max} 3370, 3088, 3027, 2920, 2859, 1578, 1517, 1271, 1225; UV-vis (EtOH): λ_{max} 405 nm; ^{1}H NMR (400 MHz, CD_3CN): δ 8.16 (bs, 1H), 7.85 (dd, J = 9.1 Hz, 3.1 Hz, 1H), 7.35-7.20 (m, 6H), 6.94 (dd, J = 9.6 Hz, 4.7 Hz, 1H), 4.10-4.03 (m, 1H), 3.43 (ddd, J = 13.2 Hz, 5.8 Hz, 3.5 Hz, 1H), 3.26-3.20 (m, 2H), 2.86 (dd, J = 13.6 Hz, 5.7 Hz, 1H); 2.80 (dd, J = 13.6 Hz, 7.5 Hz, 1H); ^{13}C NMR (100 MHz, CD_3CN): δ 153.1 (d, J = 235.2

Hz), 144.0, 139.5, 130.4, 129.3, 127.3, 125.8 (d, J = 24.0 Hz), 117.2 (d, J = 7.4 Hz), 112.3 (d, J = 26.3 Hz), 71.4, 49.1, 42.1, one signal is obscured or overlapping; ^{19}F NMR (376 MHz, CD_3CN): δ -130.5.

Synthesis of 1-((5-Fluoro-2-nitrophenyl)amino)-3-phenylpropan-2-ol (4e)

5-Fluoro-2-nitroaniline (51 mg, 0.320 mmol) and 2- benzyloxirane (66 mg, 0.480 mmol) was reacted according to Method 1 for 112 h to yield compound **4e** (14 mg, 15%) (silica gel chromatography pet. ether/EtOAc, 8:2, v/v; R_f = 0.28 in pet. ether/EtOAc, 8:2, v/v) as an orange solid, mp. 77-80 °C.

IR (neat): ν_{max} 3528, 3371, 1904, 1507, 1200; UV-vis (EtOH): λ_{max} 376 nm; ^{1}H NMR (400 MHz, CD_3CN): δ 8.36 (bs, NH), 8.19 (dd, J = 9.5, 6.2 Hz, 1H), 7.38-7.17 (m, 5H), 6.62 (dd, J = 12.1, 2.6 Hz, 1H), 6.42 (m, 1H), 4.12-4.01 (m, 1H), 3.40 (ddd, J = 13.4, 5.8, 3.6 Hz, 1H), 3.25 (d, J = 5.2 Hz, 1H), 3.25-3.19 (m, 1H), 2.87 (dd, J = 13.7, 5.7 Hz, 1H), 2.80 (dd, J = 13.7, 7.5 Hz, 1H); ^{13}C NMR (100 MHz, CD_3CN): δ 168.4 (d, J = 253.0 Hz), 148.7 (d, J = 13.8 Hz), 139.4, 130.8 (d, J = 12.6 Hz), 130.4, 129.3, 127.2, 104.4 (d, J = 25.3 Hz), 100.7 (d, J = 27.8 Hz), 71.2, 49.1, 42.1, one signal is obscured or overlapping; ^{19}F NMR (376 MHz, CD_3CN): δ -102.2.

Synthesis of 1-((2-fluoro-6-nitrophenyl)amino)-3-phenylpropan-2-ol (4f)

2-fluoro-6-nitroaniline (77 mg, 0.49 mmol) and 2-benzyloxirane (99 mg, 0.74 mmol) was reacted according to Method 1 for 72 h to yield compound **4f** (59 mg, 41%) (silica gel chromatography pet. ether/DCM 2:8, v/v; R_f = 0.20 in pet. ether/DCM 2:8, v/v; recrystallization from hexane and DCM) as a yellow solid, mp 110-113 ^{o}C.

IR (neat): ν_{max} 3540, 3365, 3085, 3026, 2917, 2860, 1523, 1495, 1341, 1243, 1081, 738, 697; UV-vis (EtOH): λ_{max} 365 nm; ^{1}H NMR (400 MHz, CD_3CN): δ 7.96-7.93 (m, 1H), 7.87 (dd, J = 12.1 Hz, 2.5 Hz, 1H), 7.33-7.21 (m, 5H), 6.72 (t, J = 8.8 Hz, 1H), 5.57 (bs, 1H), 4.02-3.97 (m, 1H), 3.36 (ddd, J = 13.6 Hz, 6.2 Hz, 3.8 Hz, 1H), 3.24-3.17 (m, 1H), 3.13 (d, J = 5.1 Hz, 1H), 2.85 (dd, J = 13.6 Hz, 5.2 Hz, 1H), 2.75 (dd, J = 13.7 Hz, 7.8 Hz, 1H); ^{13}C NMR (100 MHz, CD_3CN): δ 150.0 (d, J = 240.8 Hz), 144.4 (d, J = 11.5 Hz), 139.7, 136.9, 130.4, 129.3, 127.2, 123.2, 111.4 (d, J = 23.4 Hz), 110.7 (d, J = 4.4 Hz), 71.5, 49.1, 41.9; ^{19}F NMR (376 MHz, CD_3CN): δ -136.3.

Synthesis of 1-((2-nitro-4-(trifluoromethoxy)phenyl)amino)-3-phenylpropan-2-ol (4g)

2-nitro-4-(trifluoromethoxy)aniline (75 mg, 0.34 mmol) and 2-benzyloxirane (68 mg, 0.51 mmol) was reacted according to Method 1 for 65 h to yield compound **4g** (79 mg, 66%) (silica gel chromatography pet. ether/EtAOc 8:2, v/v; R_f = 0.28 in pet. ether/EtAOc 8:2, v/v) as an orange oil.

IR (neat): ν_{max} 3518, 3372, 2922, 1630, 1574, 1520, 1244, 1209, 1142, 1060; UV-vis (EtOH): λ_{max} 427 nm (ε 5883 $M^{-1}cm^{-1}$); ^{1}H NMR (400 MHz, CD_3CN): δ 8.29 (bs, 1H), 8.03 (d, *J* = 2.7 Hz, 1H), 7.41 (dd, *J* = 9.4 Hz, 2.5 Hz, 1H), 7.32-7.20 (m, 5H), 6.98 (d, *J* = 9.6 Hz, 1H), 4.08-4.05 (m, 1H), 3.45 (ddd, *J* = 13.4 Hz, 6.0 Hz, 3.8 Hz, 1H), 3.30-3.23 (m, 2H), 2.87 (dd, *J* = 13.6 Hz, 5.6 Hz, 1H), 2.80 (dd, *J* = 13.6 Hz, 7.3 Hz, 1H); ^{13}C NMR (100 MHz, CD_3CN): δ 145.8, 139.4, 137.9, 131.7, 131.0, 130.4, 129.3, 127.3, 121.6 (q, *J* = 255.6 Hz), 120.1, 117.1, 71.4, 49.0, 42.1; ^{19}F NMR (376 MHz, CD_3CN): δ -59.6.

Synthesis of 1-((4-fluorophenyl)amino)-3-(4-nitrophenyl)propan-2-ol (6a)

4-Fluoraniline (39 mg, 0.35 mmol) and 2-(4-nitrobenzyl)oxirane (0.93 mg, 0.52 mmol) was reacted according Method 1 for 20 h to yield compound **6a** (15 mg, 15%) (silica gel chromatography pet. ether/EtOAc, 1:1 v/v; R_f = 0.32 in pet. ether/EtOAc, 1:1 v/v) as a yellowish solid, mp 115-118 ºC.

IR (neat): ν_{max} 3275, 3111, 2909, 2841, 1604, 1507, 1338, 1218, 1075, 820; UV-vis (EtOH): λ_{max} 322 nm; ^{1}H NMR (400 MHz, $CDCl_3$): δ 8.17 (d, *J* = 8.8 Hz, 2H), 7.42 (d, *J* = 8.7 Hz, 2H), 6.91-6.87 (m, 2H), 6.57 (dd, *J* = 9.1 Hz, 4.4 Hz, 2H), 4.13-4.07 (m, 1H), 3.88 (bs, 1H), 3.27 (dd, *J* = 12.8 Hz, 3.4 Hz, 1H), 3.07 (dd, *J* = 12.9 Hz, 8.1 Hz, 1H), 2.98 (dd, *J* = 13.8 Hz, 4.8 Hz, 1H), 2.92 (dd, *J* = 13.8 Hz, 8.0 Hz, 1H); ^{13}C NMR (100 MHz, $CDCl_3$): δ 156.3 (d, *J* = 237.2 Hz), 146.9, 145.8, 144.2 (d, *J* = 2.0 Hz), 130.2, 123.7, 115.8 (d, *J* = 22.4 Hz), 114.4 (d, *J* = 7.7 Hz), 70.6, 50.5, 41.2; ^{19}F NMR (376 MHz, $CDCl_3$): δ -126.8.

Synthesis of 1-((3-fluorophenyl)amino)-3-(4-nitrophenyl)propan-2-ol (6b)

2-(4-Nitrobenzyl)oxirane (100 mg, 0.558 mmol) and 3-fluoro-aniline (41 mg, 0.372 mmol) was reacted according to Method 1 for 18 h to yield compound **6b** (84 mg, 78%) (silica gel chromatography pet. ether/EtOAc 1:1, v/v; R_f = 0.56 in pet. ether/EtOAc 1:1, v/v) as an orange solid, mp 98-101 °C.

IR (neat): ν_{max} 3509, 3346, 1900, 1491, 1335 cm^{-1}; UV-vis (EtOH): λ_{max} 300 nm; ^{1}H NMR (400 MHz, CD_3CN): δ 8.15 (d, *J* = 8.8 Hz, 2H), 7.49 (d, *J* = 8.8 Hz, 2H), 7.08 (dd, *J* = 15.4, 8.2 Hz, 1H), 6.44-6.41 (m, 1H), 6.37-6.30 (m, 2H), 4.77 (bs, 1H), 4.06-3.90 (m, 1H), 3.20 (ddd, *J* = 13.0, 6.5, 4.3 Hz, 1H), 3.12 (d, *J* = 5.2 Hz, 1H), 3.08-2.94 (m, 2H), 2.82 (dd, *J* = 13.7, 8.5 Hz, 1H); ^{13}C NMR (100 MHz, CD_3CN): δ 165.0 (d, *J* = 239.7 Hz), 151.8 (d, *J* = 11.1 Hz), 148.5, 147.6, 131.5, 131.3, 124.2, 109.7 (d, *J* = 2.1 Hz), 103.5 (d, *J* = 21.7 Hz), 99.6 (d, *J* = 25.6 Hz), 71.0, 50.1, 41.7; ^{19}F NMR (376 MHz, CD_3CN): δ -114.9.

Synthesis of 1-((2-fluorophenyl)amino)-3-(4-nitrophenyl)propan-2-ol (6c)

2-fluoroaniline (50 mg, 0.45 mmol) and 2-(4-nitrobenzyl)oxirane (81 mg, 0.45 mmol) was reacted according to Method 2 for 24 h to yield compound **6c** (47 mg, 36%) (silica gel chromatography pet ether/DCM 2:9, v/v; R_f = 0.20 in pet ether/DCM 2:9, v/v; recrystallization with hexane and DCM)) as a yellow solid, mp 98-101 ºC.

IR (neat): ν_{max} 3589, 3513, 3386, 3294, 3065, 1604, 1508, 1338, 1075, 838, 734 cm^{-1}; UV-vis (EtOH): λ_{max} 292 nm; ^{1}H NMR (400 MHz, CD_3CN): δ 8.14 (d, *J* = 8.7 Hz, 2H), 7.48 (d, *J* = 8.6 Hz, 2H), 7.00-6.96 (m, 2H), 6.75-6.71 (m, 1H), 6.63-6.58 (m, 1H), 4.53 (bs, 1H), 4.06-3.99 (m, 1H), 3.27 (ddd, *J* = 13.0 Hz, 6.7 Hz, 4.1 Hz, 1H), 3.18 (d, *J* = 5.3 Hz, 1H), 3.12-3.06 (m, 1H), 2.99 (dd, *J* = 13.8 Hz, 4.4 Hz, 1H), 2.84 (dd, *J* = 13.8 Hz, 8.5 Hz, 1H); ^{13}C NMR (100 MHz, CD_3CN): δ 152.5 (d, *J* = 236.4 Hz), 148.5, 147.7, 137.9, (d, *J* = 11.4 Hz), 131.5, 125.7 (d, *J* = 3.5 Hz), 124.2, 117.4 (d, *J* = 7.1 Hz), 115.2 (d, *J* = 18.6 Hz), 113.3 (d, *J* = 3.6 Hz), 71.0, 49.9, 41.8; ^{19}F NMR (376 MHz, CD_3CN): δ -137.8.

Synthesis of 1-((4-bromophenyl)amino)-3-(4-nitrophenyl)propan-2-ol (6d)

4-bromoaniline (80 mg, 0.47 mmol) and 2-(4-nitrobenzyl)oxirane (83 mg, 0.47 mmol) was reacted according to Method 2 for 23 h to yield compound **6d** (80 mg, 50%) (silica gel chromatography pet. ether/EtOAc 6:4, v/v; R_f = 0.30 in pet. ether/EtOAc 6:4, v/v) as a yellow solid, mp 117-120 ºC.

IR (neat): ν_{max} 3498, 3413, 3373, 3070, 2840, 1591, 1505, 1336, 1096, 1066, 818, 741, 696 cm^{-1}; UV-vis (EtOH): λ_{max} 320 nm; ^{1}H NMR (400 MHz, CD_3CN): δ 8.14 (d, *J* = 8.7 Hz, 2H), 7.47 (d, *J* = 8.7 Hz, 2H), 7.21 (d, *J* = 8.9 Hz, 2H), 6.55 (d, *J* = 8.9 Hz, 2H), 4.66 (t, *J* = 4.9 Hz, 1H), 4.01-3.94 (m, 1H), 3.18 (ddd, *J* = 13.1 Hz, 6.5 Hz, 4.3 Hz, 1H), 3.11 (d, *J* = 5.1 Hz, 1H), 3.04-2.95 (m, 2H), 2.81 (dd, *J* = 13.7 Hz, 8.4 Hz, 1H); ^{13}C NMR (100 MHz, CD_3CN): δ 149.0, 148.5, 147.6, 132.6, 131.5, 124.2, 115.3, 108.4, 71.0, 50.1, 41.7.

Synthesis of 1-((3-bromophenyl)amino)-3-(4-nitrophenyl)propan-2-ol (6e)

3-Bromoaniline (80 mg, 0.47 mmol) and 2-(4-nitrobenzyl)oxirane (83 mg, 0.47 mmol) was reacted according to Method 2 for 65 h to yield compound **6e** (91 mg, 56%) (silica gel chromatography pet. ether/DCM 2:8, v/v; R_f = 0.22 in pet. ether/DCM 2:8, v/v; recrystallization with hexane and DCM) as a yellow solid, mp 112-115 ºC.

IR (neat): ν_{max} 3517, 3329, 2919, 2839, 1596, 1502, 1401, 1337, 765 cm^{-1}; UV-vis (EtOH): λ_{max} 291 nm; ^{1}H NMR (400 MHz, CD_3CN): δ 8.14 (d, *J* = 8.7 Hz, 1H), 7.47 (d, *J* = 8.8 Hz, 2H), 7.01 (t, *J* = 8.0 Hz, 1H), 6.76-6.72 (m, 2H), 6.59-6.57 (m, 1H), 4.73 (bs, 1H), 4.00-3.94 (m, 1H), 3.19 (ddd, *J* = 13.2 Hz, 6.6 Hz, 4.2 Hz, 1H), 3.13 (d, *J* = 5.2 Hz, 1H), 3.05-2.95 (m, 2H), 2.82 (dd, *J* = 13.6 Hz, 8.5 Hz, 1H); ^{13}C NMR (100 MHz, CD_3CN): δ 151.3, 148.5, 147.6, 131.6, 123.8, 123.6, 120.0, 115.6, 112.6, 70.0, 49.9, 41.7, one signal is obscured or overlapping.

Synthesis of 1-((2-bromophenyl)amino)-3-(4-nitrophenyl)propan-2-ol (6f)

2-bromoaniline (80 mg, 0.47 mmol) and 2-(4-nitrobenzyl)oxirane (83 mg, 0.47 mmol) was reacted according to Method 2 for 65 h to yield compound **6f** (25 mg, 15%) (silica gel chromatography pet. ether/DCM 3:7, v/v → chloroform/methanol, 99:1, v/v; R_f = 0.20 in pet. ether/DCM 3:7, v/v) as a beige solid, mp 83-86 ºC.

IR (neat): ν_{max} 3397, 3202, 3073, 2929, 2841, 1593, 1508, 1336, 1105, 1061, 1016, 735 cm^{-1}; UV-vis (EtOH): λ_{max} 300 nm; ^{1}H NMR (400 MHz, CD_3CN): δ 8.14 (d, J = 8.7 Hz, 2H), 7.48 (d, J = 8.6 Hz, 2H), 7.40 (dd, J = 7.9 Hz, 1.4 Hz, 1H), 7.20-7.16 (m, 1H), 6.68 (dd, 8.2 Hz, 1.2 Hz, 1H), 6.58-6.54 (m, 1H), 4.78 (bs, 1H), 4.08-4.03 (m, 1H), 3.32-3.26 (m, 2H), 3.14-3.07 (m, 1H), 2.99 (dd, J = 13.6 Hz, 4.4 Hz, 1H), 2.87 (dd, J = 13.6 Hz, 8.2 Hz, 1H); ^{13}C NMR (100 MHz, CD_3CN): δ 148.3, 147.7, 146.2, 133.3, 131.5, 129.6, 124.2, 118.7, 112.7, 110.1, 70.9, 49.9, 41.9.

Synthesis of 1-((2-iodophenyl)amino)-3-(4-nitrophenyl)propan-2-ol (6g)

2-Iodoaniline (100 mg, 0.46 mmol) and 2-(4-nitrobenzyl)oxirane (90 mg, 0.50 mmol) was reacted according to Method 2 for 96 h to yield compound **6g** (61 mg, 34%) (silica gel chromatography chloroform/methanol 99:1, v/v; R_f = 0.20 in chloroform/methanol 99:1, v/v) as a yellow oil.

IR (neat): ν_{max} 3526, 3377, 3070, 2919, 2849, 1586, 1508, 1341, 1314, 698 cm^{-1}; UV-vis (EtOH): λ_{max} 314 nm; ^{1}H NMR (400 MHz, CD_3CN): δ 8.14 (d, J = 8.8 Hz, 2H), 7.65 (dd, J = 7.8 Hz, 1.5 Hz, 1H), 7.47 (d, J = 8.8 Hz, 2H), 7.22-7.18 (m, 1H), 6.59 (dd, J = 8.2 Hz, 1.3 Hz, 1H), 6.45-6.41 (m, 1H), 4.61 (bs, 1H) 4.07-4.03 (m, 1H), 3.31-3.26 (m, 2H), 3.12-3.05 (m, 1H), 2.99 (dd, J = 13.8 Hz, 4.8 Hz, 1H), 2.87 (dd, J = 13.8, 8.2 Hz, 1H); ^{13}C NMR (100 MHz, CD_3CN): δ 148.5, 148.3, 147.7, 140.0, 131.5, 130.5, 124.2, 119.6, 111.9, 85.6, 70.8, 50.2, 41.9.

Synthesis of 1-(4-nitrophenyl)-3-((4-(trifluoromethoxy)phenyl)amino)propan-2-ol (6h)

4-(trifluoromethoxy)aniline (65 mg, 0.34 mmol) and 2-(4-nitrobenzyl)oxirane (100 mg, 0.51 mmol) was reacted according to Method 1 for 15 h to yield compound **6h** (119 mg, 90%) (silica gel chromatography pet. ether/EtOAc 1:1, v/v; R_f = 0.50 in pet. ether/EtOAc 1:1, v/v) as a white solid, mp 97-100 ºC.

IR (neat): ν_{max} 3282, 3198, 2919, 2848, 1599, 1509, 1342, 1266, 1202, 1049 cm^{-1}; UV/vis (EtOH): λ_{max} 325 nm; ^{1}H NMR (400 MHz, $CDCl_3$): δ 8.18 (d, J = 8.7 Hz, 2 H), 7.40 (d, J = 8.7 Hz, 2H), 7.04 (d, J = 8.8 Hz, 2H), 6.59 (d, J = 9.0 Hz, 2H), 4.15-4.09 (m, 2H), 3.30 (dd, J = 13.0 Hz, 3.4 Hz, 1H), 3.11 (dd, J = 13.0 Hz, 8.0 Hz, 1H), 3.00 (dd, J = 13.8 Hz, 4.8 Hz, 1H), 2.92 (dd, J = 13.7 Hz, 8.0 Hz, 1H), 2.06 (bs, 1H); ^{13}C NMR (100 MHz,

$CDCl_3$): δ 147.1, 146.8, 145.8, 141.1 (d, J = 1.9 Hz), 130.4, 123.9, 122.6, 120,8 (q, J = 255.0 Hz), 113.7, 70.7, 49.9, 41.3; ^{19}F NMR (376 MHz, $CDCl_3$): δ -58.5.

Synthesis of 1-((4-difluoromethoxyphenyl)amino)-3-(4-nitrophenyl)propan-2-ol (6i)

4-(Difluoromethoxy)aniline (69.9 mg, 0.440 mmol) and 2-(4-nitrobenzyl)oxirane (111.2 mg, 0.621 mmol) was reacted according to Method 1 for 24 h to yield compound **6i** (38.7 mg, 26%) (silica gel chromatography pet. ether/EtOAc 1:1, vv; R_f = 0.36 in pet. ether/EtOAc 1:1, vv) as a white solid, mp 101-104.5 °C.

IR (neat): ν_{max} = 3277, 3210, 2910, 2845, 1514, 1342, 1115 cm^{-1}; UV-vis (EtOH) λ_{max} 324 nm; ^{1}H NMR (400 MHz, $CDCl_3$): δ 8.20 (d, J = 8.8 Hz, 2H), 7.43 (d, J = 8.8 Hz, 2H), 6.97 (d, J = 8.9, 2H), 6.60-6.58 (m, 2H), 6.38 (t, J = 74.5 Hz, 1H), 4.14-4.09 (m, 1H), 3.99 (bs, 1H), 3.30 (dd, J = 13.0 Hz, 4.2 Hz, 1H), 3.10 (d, J = 4.1 Hz, 1H), 3.03-2.87 (m, 2H), 2.03 (d, J = 5.1 Hz, 1H); ^{13}C NMR (100 MHz, CD_3CN): δ 147.1, 145.9, 145.8, 130.3, 123.9, 121.8, 116.5, 114.1, 70.8, 50.2, 41.3, one signal is obscured or overlapping; ^{19}F NMR (376 MHz, CD_3CN): δ –81.5.

Synthesis of 1-((2-bromo-4-trifluoromethoxyphenyl)amino)-3-(4-nitorphenyl)propan-2-ol (6j)

2-Bromo-4-trifluoromethylaniline (161.0 mg, 0.629 mmol) and 2-(4-nitrobenzyl)oxirane (116.7 mg, 0.652 mmol) was reacted according to Method 2 for 5 days to yield compound **6j** (97.1 mg, 36%) (silica gel chromatography pet. ether/DCM 3:7, v/v; R_f = 0.16 in pet. ether/DCM 3:7, v/v) as a white solid, mp 92.1-99.5 °C.

IR (neat): ν_{max} 3527, 3389, 2911, 2849, 1511, 1345, 1225 cm^{-1}; UV-vis (EtOH) λ_{max} 303 nm (ε 1319 $M^{-1}cm^{-1}$); ^{1}H NMR (400 MHz, CD_3CN): δ 8.15 (d, J = 8.8 Hz, 2H), 7.48 (d, J = 8.8 Hz, 2H), 7.43-7.42 (m, 1H), 7.16-7.13 (m, 1H), 6.70 (d, J = 9.0 Hz, 1H), 4.89 (t, J = 5.7 Hz, 1H), 4.09-4.02 (m, 1H), 3.31 (ddd, J = 13.0, 6.5, 3.9 Hz, 1H), 3.25 (d, J = 5.4 Hz, 1H), 3.15-3.09 (m, 1H), 2.99 (dd, J = 13.7, 4.5 Hz, 1H), 2.86 (dd, J = 13.7, 8.3 Hz, 1H); ^{13}C NMR (100 MHz, CD_3CN): δ 148.2, 147.7, 145.7, 139.9, 131.5, 126.6, 124.3, 122.8, 112.3, 109.0, 70.8, 50.0, 41.8, one signal is obscured or overlapping; ^{19}F NMR (376 MHz, CD_3CN) : δ - 59.5.

Synthesis of 1-((4-methyl-3-trifluoromethylphenyl)amino)-3-(4-nitorphenyl)propan-2-ol (6k)

4-Methyl-3-trifluoromethylaniline (126.0 mg, 0.719 mmol) and 2-(4-nitrobenzyl)oxirane (102.1 mg, 0.570 mmol) was reacted according to Method 2 for 8 h to yield compound **6k** (119.5 mg, 59%) (silica gel chromatography pet. ether/DCM 2:8, v/v; Rf = 0.09 in pet. ether/DCM 2:8, v/v) as a white solid, mp 96.8-101.1 °C.

IR (neat): ν_{max} = 3315, 3211, 3077, 2924, 2852, 1510, 1338 cm^{-1}; UV-vis (EtOH) λ_{max} 304 nm; ^{1}H NMR (400 MHz, CD_3CN): δ 8.14 (d, J = 8.8 Hz, 2H), 7.48 (d, J = 8.8 Hz, 2H), 7.09 (d, J = 8.3 Hz, 1H), 6.87 (d, J = 2.5 Hz, 1H), 6.73 (dd, J = 8.3, 2.4 Hz, 1H), 4.69 (bs, 1H), 4.00-3.95 (m, 1H), 3.22 (ddd, J = 13.1, 6.7, 4.2 Hz, 1H), 3.12 (d, J = 5.2 Hz, 1H), 3.07-3.00 (m, 1H), 2.98 (dd, J = 13.6, 4.8Hz, 1H), 2.83 (dd, J = 13.7, 8.4 Hz, 1H), 2.30 (q, J = 1.8 Hz, 3H); ^{13}C NMR (100 MHz, CD_3CN): δ 148.5, 147.9, 147.6, 133.8, 131.5, 129.6, 128.3 (q, J = 223,4 Hz), 124.7, 124.2, 116.6, 110.7 (q, J = 5.8 Hz), 71.1, 50.1, 41.7, 18.3 (q, J = 2.0 Hz); ^{19}F NMR (376 MHz, CD_3CN) : δ - 62.1.

Synthesis of 1-((6-bromo-2,3,4-trifluorophenyl)amino)-3-(4-nitrophenyl)propan-2-ol (6l)

2-(4-Nitrobenzyl)oxirane (59 mg, 0.33 mmol) and 6-bromo-2,3,4-trifluoroaniline (75 mg, 0.33 mmol) was reacted according to Method 1 for 38 h to yield compound **6l** (57 mg, 43%) (silica gel chromatography DCM; R_f = 0.57 in DCM) as a white solid, mp 80-83 °C.

IR: ν_{max} 3547, 3341, 1930, 1501, 1350, 699 cm^{-1}; UV-vis (EtOH): λ_{max} 311 nm (ε 1920 M^{-1} cm^{-1}); ^{1}H NMR (400 MHz, CD_3CN): δ 8.14 (d, J = 8.8 Hz, 2H), 7.46 (d, J = 8.7 Hz, 2H), 7.33 (ddd, J = 10.1, 7.8, 2.5 Hz, 1H), 4.44 (bs, 1H), 4.01-3.90 (m, 1H), 3.52-3.41 (m, 1H), 3.24-3.19 (m, 2H), 2.95 (dd, J = 13.7, 4.5 Hz, 1H), 2.82 (dd, J = 13.7, 8.3 Hz, 1H); ^{13}C NMR (100 MHz, CD_3CN): δ 148.2, 147.7, 144.2 (ddd, J = 241.2, 11.1, 3.3 Hz), 143.1 (ddd, J = 246.3, 12.5, 3.7 Hz), 141.3 (app. dt, J = 246.9, 15.7 Hz), 134.4 (d, J = 8.7 Hz), 131.5, 124.2, 116.3 (dd, J = 20.8, 3.5 Hz), 106.2 (ddd, J = 9.9, 5.8, 4.1 Hz) 71.8, 52.4, 41.6; ^{19}F NMR (376 MHz, CD_3CN): δ -149.7, -161.4.

Synthesis of 1-((4-nitrophenyl)amino)-3-(4-nitrophenyl)propan-2-ol (6m)

2-(4-Nitrobenzyl)oxirane (116.5 mg, 0.651 mmol) and 4-nitroaniline (60.7 mg, 0.439 mmol) was reacted according to Method 1 for 36 h to yield compound **6m** (65.9 mg, 47%) (silica gel chromatography pet. ether/EtOAc 1:1, v/v; R_f = 0.14 in pet. ether/EtOAc 1:1, v/v) as a light-yellow solid, mp 157-161 °C.

IR (neat): ν_{max} 3446, 3315, 1595, 1276, 1097, 836 cm^{-1}; UV-vis (EtOH): λ_{max} 318 nm; ^{1}H NMR (400 MHz, $CDCl_3$): δ 8.21 (d, J = 8.7 Hz, 2H), 8.09 (d, J = 9.2 Hz, 2H), 7.43 (d, J = 8.7 Hz, 2H), 6.57 (d, J = 9.2 Hz, 2H) ; 4.86 (bs, 1H), 4.19-4.15 (m, 1H), 3.43 (ddd, J = 13.2, 6.2, 3.4 Hz, 1H), 3.27-3.21 (m, 1H), 3.02 (dd, J = 13.7, 4.7 Hz, 1H), 2.94 (dd, J = 13.7, 8.3 Hz, 1H); ^{13}C NMR (100 MHz, $CDCl_3$): δ 153.1, 147.3, 145.1, 138.8, 130.4, 126.6, 124.1, 111.6, 70.7, 48.7, 41.4.

Synthesis of 1-((2-fluoro-4-nitrophenyl)amino)-3-(4-nitrophenyl)propan-2-ol (6n)

2-fluoro-4-nitroaniline (50 mg, 0.32 mmol) and 2-(4-nitrobenzyl)oxirane (86 mg, 0.48 mmol) was reacted according to Method 1 for 48 h to yield compound **6n** (82 mg, 76%) (silica gel chromatography pet. ether/EtOAc 7:3, v/v; R_f = 0.20 in pet. ether/EtOAc 7:3, v/v; recrystallization with hexane and DCM)) as a yellow solid, mp 132-135 ºC.

IR (neat): ν_{max} 3469, 3294, 3091, 2911, 1606, 1548, 1514, 1492, 1285, 1191, 1084, 733; UV-vis (EtOH): λ_{max} 380 nm; ^{1}H NMR (400 MHz, CD_3CN): δ 8.17 (d, *J* = 8.7 Hz, 2H), 7.53-7.48 (m, 4H), 7.14 (dd, *J* = 11.2 Hz, 8.7 Hz, 1H), 5.01 (bs, 1H), 4.05-4.01 (m, 1H), 3.35 (ddd, *J* = 13.4 Hz, 6.5 Hz, 4.1 Hz, 1H), 3.26 (d, *J* = 5.2 Hz, 1H), 3.22-3.16 (m, 1H), 3.00 (dd, *J* = 13.8 Hz, 4.5 Hz, 1H), 2.86 (dd, *J* = 13.8 Hz, 8.4 Hz, 1H); ^{13}C NMR (100 MHz, CD_3CN): δ 155.7 (d, *J* = 248.6 Hz), 148.3, 147.7 (proved by ^{1}H-^{13}C HMBC analysis), 146.3 (proved by ^{1}H-^{13}C HMBC analysis), 138.8 (d, *J* = 13.3 Hz) (proved by ^{1}H-^{13}C HMBC analysis), 131.5, 124.3, 115.6 (d, *J* = 21.5 Hz), 112.8 (d, *J* = 8.4 Hz), 107.5 (d, *J* = 6.1 Hz), 71.0, 49.4, 41.6; ^{19}F NMR (376 MHz, CD_3CN): δ -127.2.

Synthesis of 1-((4-fluoro-2-nitrophenyl)amino)-3-(4-nitrophenyl)propan-2-ol (6o)

4-Fluoro-2-nitroaniline (58 mg, 0.38 mmol) and 2-(4-nitrobenzyl)oxirane (100 mg, 0.56 mmol) was reacted according to Method 1 for 48 h to yield compound **6o** (89 mg, 71%) (silica gel chromatography pet. ether/EtOAc, 7:3 v/v; R_f = 0.20 in pet. ether/EtOAc, 7:3 v/v) as a red solid, mp 126-129 ºC.

IR (neat): ν_{max} 3498, 3372, 2926, 2853, 1579, 1511, 1341, 1241, 1241, 855, 710; UV-vis (EtOH): λ_{max} 436 nm; ^{1}H NMR (400 MHz, CD_3CN): δ 8.15 (d, *J* = 9.5 Hz, 2H), 7.85 (dd, *J* = 9.5 Hz, 3.0 Hz, 1H), 7.48 (d, *J* = 8.8 Hz, 2H), 7.37-7.32 (m, 1H), 7.00 (dd, *J* = 9.5 Hz, 4.7 Hz, 1H), 4.11-4.09 (m, 1H), 3.47-3.44 (m, 1H), 3.37 (d, *J* = 5.4 Hz, 1H), 3.32-3.25 (m, 1H), 3.00 (dd, *J* = 13.7 Hz, 4.6 Hz, 1H), 2.90 (dd, *J* = 13.7 Hz, 8.2 Hz, 1H); ^{13}C NMR (100 MHz, CD_3CN): δ 153.2 (d, *J* = 235.2 Hz), 147.9, 147.7, 143.9, 131.5, 125.8 (d, *J* = 24.0 Hz), 124.3, 117.2 (d, *J* = 7.4 Hz), 112.3 (d, *J* = 26.7 Hz), 70.8, 49.3, 41.8, one signal is obscured or overlapping; ^{19}F NMR (376 MHz, CD_3CN): δ -130.3.

Synthesis of 1-((5-Fluoro-2-nitrophenyl)amino)-3-(4-nitrophenyl)propan-2-ol (6p)

5-Fluoro-2-nitroaniline (66 mg, 0.427 mmol) and 2-(4-nitrobenzyl)oxirane (114 mg, 0.640 mmol) was reacted according to Method 1 for 88 h to yield compound **6p** (12 mg, 8%) (silica gel chromatography pet. ether/EtOAc 1:1, v/v; R_f = 0.23 in pet. ether/EtOAc, 7:3, v/v) as a yellow solid, mp 100-103 °C.

IR (neat): ν_{max} 3478, 3348, 1918, 1505, 1342 cm^{-1}; UV-vis (EtOH): λ_{max} 432 nm; ^{1}H NMR (400 MHz, CD_3CN): δ 8.34 (bs, 1H), 8.19 (dd, *J* = 9.5, 6.2 Hz, 1H), 8.15 (d, *J* = 8.8 Hz, 2H), 7.49 (d, *J* = 8.8 Hz, 2H), 6.69 (dd, *J* =

12.1, 2.6 Hz, 1H), 6.43 (ddd, J = 9.6, 7.6, 2.6 Hz, 1H), 4.16- 4.08 (m, 1H), 3.47-3.38 (m, 1H), 3.39 (d, J = 5.4 Hz, 1H), 3.30-3.23 (m, 1H), 3.0 (dd, J = 13.7, 4.6 Hz, 1H), 2.89 (dd, J = 13.7, 8.2 Hz, 1H); ^{13}C NMR (100 MHz, CD_3CN): δ 167.8 (d, J = 256.7 Hz), 148.7 (d, J = 13.2 Hz), 147.8, 131.6, 130.9 (d, J = 12.4 Hz), 130.4, 124.3, 104.6 (d, J = 24.6 Hz), 100.9 (d, J = 27.3 Hz), 70.7, 49.3, 41.7, one signal is obscured or overlapping; ^{19}F NMR (376 MHz, CD_3CN): δ -96.9.

Synthesis of 1-((2-fluoro-6-nitrophenyl)amino)-3-(4-nitrophenyl)propan-2-ol (6q)

2-Fluoro-6-nitroaniline (63 mg, 0.41 mmol) and 2-(4-nitrobenzyl)oxirane (109 mg, 0.61 mmol) was reacted according to Method 1 for 66 h to yield compound **6q** (47 mg, 36%) (silica gel chromatography pet. ether/EtAOc 1:1, v/v; R_f = 0.34 in pet. ether/EtAOc 1:1, v/v; recrystallization with hexane and DCM)) as a yellow solid, mp 142-145 ºC.

IR (neat): ν_{max} 3453, 3345, 3104, 2935, 1633, 1522, 1343, 868, 839, 740; UV/vis (EtOH): λ_{max} 365 nm; ^{1}H NMR (400 MHz, CD_3CN): δ 8.15 (d, J = 8.8 Hz, 2H), 7.98-7.95 (m, 1H), 7.87 (dd, J = 12.0 Hz, 2.6 Hz, 1H), 7.48 (d, J = 8.8 Hz, 2H), 6.79 (t, J = 8.9 Hz, 1H), 5.60 (bs, 1H), 4.09-4.01 (m, 1H), 3.40 (ddd, J = 13.6 Hz, 6.2 Hz, 4.0 Hz, 1H), 3.29-3.22 (m, 2H), 2.99 (dd, J = 13.7 Hz, 4.2 Hz, 1H), 2.84 (dd, J = 13.7 Hz, 8.6 Hz, 1H); ^{13}C NMR (100 MHz, CD_3CN): δ 151.2 (d, J = 242.4 Hz), 148.1, 147.7, 144.3 (d, J = 11.4 Hz), 137.0 (d, J = 8.4 Hz), 131.5, 124.2, 123.2 (d, J = 2.1 Hz), 111.5 (d, J = 23.4 Hz), 110.8 (d, J = 4.3 Hz), 70.9, 49.2, 41.5; ^{19}F NMR (376 MHz, CD_3CN): δ -136.0.

Synthesis of 1-((2-nitro-4-(trifluoromethoxy)phenyl)amino)-3-(4-nitrophenyl)propan-2-ol (6r)

2-nitro-4-(trifluoromethoxy)aniline (75 mg, 0.34 mmol) and 2-(4-nitrobenzyl)oxirane (90 mg, 0.51 mmol) was reacted according to Method 1 for 65 h to yield compound **6r** (63 mg, 46%) (silica gel chromatography pet. ether/EtOAc 7:3, v/v; R_f = 0.23 in pet. ether/EtOAc 7:3, v/v; recrystallization with hexane and DCM) as a yellow solid, mp 140-143 ºC.

IR (neat): ν_{max} 3537, 3369, 3117, 2926, 1512, 1346, 1220, 1187, 821; UV-vis (EtOH): λ_{max} 426 nm; ^{1}H NMR (400 MHz, CD_3CN): δ 8.26 (bs, 1H), 8.14 (d, J = 8.8 Hz, 2H), 8.03 (d, J = 2.7 Hz, 1H), 7.48 (d, J = 8.8 Hz, 2H), 7.44-7.42 (m, 1H), 7.04 (d, J = 9.5 Hz, 1H), 4.15-4.08 (m, 1H), 3.49 (ddd, J = 13.4 Hz, 5.9 Hz, 3.9 Hz, 1H), 3.40 (d, J = 5.4 Hz, 1H), 3.35-3.28 (m, 1H), 3.00 (dd, J = 13.8 Hz, 4.7 Hz, 1H), 2.90 (dd, J = 13.7 Hz, 8.2 Hz, 1H); ^{13}C NMR (100 MHz, CD_3CN): δ 147.9, 147.7, 145.7, 138.0 (d, J = 2.1 Hz), 131.8, 131.5, 131.0, 124.3, 122.9 (q, J = 255.6 Hz), 120.2, 117.2, 70.8, 49.2, 41.7; ^{19}F NMR (376 MHz, CD_3CN): δ -59.6.

Synthesis of 1-(4-chlorophenyl)-3-((4-nitrophenyl)amino)propan-2-ol (8a)

2-(4- Chlorobenzyl)oxirane (50 mg, 0.296 mmol) and 4-nitro-aniline (41 mg, 0.296 mmol) was reacted according to Method 2 for 23 h to yield compound **8a** (16 mg, 18%) (silica gel chromatography DCM; R_f = 0.25 in DCM) as a yellow powder, mp 156-159 °C.

IR: ν_{max} 3451, 3304, 2921, 1773, 1592, 797 cm^{-1}; UV-vis (EtOH): λ_{max} 341 nm; ^1H NMR (400 MHz, CD_3CN): δ 8.01 (d, J = 9.3 Hz, 2H), 7.31 (d, J = 8.5 Hz, 2H), 7.24 (d, J = 8.5 Hz, 2H), 6.62 (d, J = 9.3 Hz, 2H), 5.70 (bs, 1H), 3.96-3.92 (m, 1H), 3.32-3.26 (m, 1H), 3.13 (d, J = 5.2 Hz, 2H), 2.84 (dd, J = 13.8, 4.8 Hz, 1H), 2.71 (dd, J = 13.7, 8.1 Hz, 1H); ^{13}C NMR (100 MHz, CD_3CN): δ 155.4 , 138.7, 138.1, 132.1, 129.1, 129.0, 127.0, 112.0, 71.2, 49.3, 41.2.

Synthesis of 1-(4-chlorophenyl)-3-((3-methyl-4-nitrophenyl)amino)propan-2-ol (8b)

2-(4-Chlorobenzyl)oxirane (50 mg, 0.296 mmol) and 3-chloro-4-nitro-aniline (51 mg, 0.296 mmol) was reacted according to Method 2 for 23 h to yield compound **8b** (29 mg, 29%) (silica gel chromatography DCM; R_f = 0.39 in DCM) as a yellow powder, mp 123-126 °C.

IR (neat): ν_{max} 3538, 3350, 3095, 2920, 1912, 1594, 795 cm^{-1}; UV-vis (EtOH): λ_{max} 316 nm; ^1H NMR (400 MHz, CD_3CN): δ 7.95 (d, J = 9.2 Hz, 1H), 7.31 (d, J = 8.4 Hz, 2H), 7.24 (d, J = 8.4 Hz, 2H), 6.68 (d, J = 2.6 Hz, 1H), 6.58 (d, J = 9.2, 2.6 Hz, 1H), 5.72 (bs, 1H), 3.96-3.88 (m, 1H), 3.26 (ddd, J = 13.6, 6.2, 3.9 Hz, 1H), 3.12 (d, J = 5.2 Hz, 1H), 3.12-3.05 (m, 1H), 2.84 (dd, J = 13.7, 5.0 Hz, 1H), 2.70 (dd, J = 13.7, 8.0 Hz, 1H); ^{13}C NMR (100 MHz, CD_3CN): δ 154.5, 138.7, 135.2 (determined by ^1H-^{13}C HMBC), 132.1, 130.9, 130.3 (determined by ^1H-^{13}C HMBC), 129.8, 129.2, 114.1, 111.2, 71.2, 49.2, 41.1.

Procedures for preparing precursors for the ethanoalamine synthesis

Synthesis of 1-allyl-4-nitrobenzene

A 25 mL round-bottom flask fitted with a condenser was charged with (4-nitrophenyl)boronic acid (0.50 g, 3.0 mmol), allyl bromide (1,09 g, 9.0 mmol), a solution of K_2CO_3 (1,24 g in 5 mL of water, 9 mmol), $Pd(PPh_3)_4$ (0,150 g, 5 mol%), THF (20 mL), and water (5 mL). The reaction mixture was refluxed under an atmosphere of argon for 48 h. After cooling to rt, the product mixture was evaporated onto celite and purified by silica-gel flash chromatography. Concentration of the relevant fractions (R_f = 0.47 (pet. ether/EtOAc 97:3, v/v)) yielded 1-allyl-4-nitrobenzene (279 mg, 57%) as a slightly yellow liquid.

IR (neat): ν_{max} 3091, 3017, 2918, 1593, 1502; ^{1}H NMR (400 MHz, $CDCl_3$): δ 8.15 (d, J = 8.8 Hz, 2H), 7.34 (d, J = 8.8 Hz, 2H), 5.99-5.89 (m, 1H), 5.18-5.09 (m, 2H), 3.49 (d, J = 6.7 Hz, 2H); ^{13}C NMR (100 MHz, $CDCl_3$): δ 147.9, 146.7, 135,6, 129.5, 123.8, 117.5, 40.0. The spectroscopic data were in accordance with previously reported data.[2,3]

Synthesis of 2-(4-nitrobenzyl)oxirane

A 25 mL round-bottom flask fitted with 1-allyl-4-nitrobenzene (0.5138 g, 3.15 mmol) in anhydrous DCM (20 mL) was cooled (ice bath) and stirred under an atmosphere of argon followed by addition of *m*CPBA (1.36 g, 7.87 mmol). The reaction mixture was stirred at ambient temperature for 48 h. The mixture was quenched with sat. aq. $Na_2S_2O_3$ solution (40 mL) and 5 M aq. K_2CO_3 solution (40 mL). The phases were separated, and the aq. phase was extracted with DCM (3 x 20 mL). The combined organic phases were dried with $MgSO_4$, filtered, and evaporated onto celite and purified by silica-gel flash chromatography. Concentration of the relevant fractions (R_f = 0.30 (pet. ether/EtOAc 8:2 v/v)) yielded 2-(4-nitrobenzyl)oxirane (0.534 g, 95%) as a slightly yellow liquid. The spectroscopic data were in accordance with previously reported data.[4]

IR (neat): ν_{max} 2957, 2923, 2853, 1599, 1516, 1345; ^{1}H NMR (400 MHz, $CDCl_3$): δ 8.18 (d, J = 8.5 Hz, 2H), 7.43 (d, J = 8.5 Hz, 2H), 3.20-3.16 (m, 1H), 3.06 (dd, J = 14.8 Hz, 4.2 Hz, 1H), 2.90 (dd, J = 14.8 Hz, 6.4 Hz, 1H), 2.84 (dd, J = 4.7 Hz, 4.0 Hz, 1H), 2.55 (dd, J = 4.7 Hz, 2.6 Hz, 1H); ^{13}C NMR (100 MHz, $CDCl_3$): δ 147.1, 145.0, 130.0, 123.9, 51.7, 46.8, 38.6.

General procedure for the synthesis of the protected anilines

The appropriate aniline (1.0 equiv.) was dissolved in 5 mL of DCM and put in ice bath, then acetyl chloride (1.6 equiv.) was added dropwise followed by the addition of triethylamine (1.6 equiv.). The mixture was stirred for 30 minutes at ambient temperature, then 30 minutes at room temperature. 15 mL of water was added to the mixture, then 15 mL of a saturated solution of $NaHCO_3$, then extracted with DCM (3 x 30 mL), and the combined organic phases were dried with $MgSO_4$, filtered, and evaporated onto celite and purified by silica-gel flash chromatography.

Synthesis of *N*-(4-fluorophenyl)acetamide

4-fluoraniline (200 mg, 1.80 mmol) was dissolved in 5 mL of DCM, then acetyl chloride (0.21 mL, 2.88 mmol) was added followed by the addition of triethylamine (0.40 mL, 2.88 mmol) according to the general procedure

for 1 h to give the title compound (252 mg, 92%) (R_f = 0.30 (pet. ether/EtOAc 1:1 v/v)) as a white solid, mp. 152-156 ºC (lit.[5]153-155 ºC).

IR (neat): ν_{max} 3288, 3267, 3148, 3071, 1660, 1614, 1504, 831; ^1H NMR (400 MHz, $CDCl_3$): δ 7.46-7.43 (m, 2H), 7.32 (bs, 1H), 7.03-6.98 (m, 2H), 2.17 (s, 3H); ^{13}C NMR (100 MHz, $CDCl_3$): δ 168.4, 159.5 (d, J = 243.6 Hz), 134.0 (d, J = 2.5 Hz), 121.9 (d, J = 7.94 Hz), 115.8 (d, J= 22.4 Hz), 24.6; ^{19}F NMR (376 MHz, $CDCl_3$): δ -118.0. The spectroscopic data were in accordance with previously reported data.[2]

Synthesis of *N*-(2-fluorophenyl)acetamide

2-fluoraniline (200 mg, 1.80 mmol) was dissolved in 5 mL of DCM, then acetyl chloride (0.21 mL, 2.88 mmol) was added followed by the addition of triethylamine (0.40 mL, 2.88 mmol) according to the general procedure for 1 h to give the title compound (272 mg, 99%) (R_f = 0.25 (pet. ether/EtOAc 8:2 v/v)) as a white solid, mp. 78-80 ºC (lit.[5] 84-86 ºC).

IR (neat): ν_{max} 3244, 3190, 3131, 3063, 3026, 1661, 1615, 1544, 1454, 732; ^1H NMR (400 MHz, $CDCl_3$): δ 8.29 (t, J = 8.0 Hz, 1H), 7.43 (bs, 1H), 7.14-7.02 (m, 3H), 2.22 (s, 3H); ^{13}C NMR (100 MHz, $CDCl_3$): δ 168.4, 152.4 (d, J = 242.7 Hz), 126.5 (d, J = 9.8 Hz), 124.7 (d, J = 3.6 Hz), 124.4 (d, J = 7.7 Hz), 121.9, 114.9 (d, J = 19.2 Hz), 24.8; ^{19}F NMR (376 MHz, $CDCl_3$): δ -131.4.

Synthesis of *N*-(4-(trifluoromethoxy)phenyl)acetamide

4-(trifluormethoxy)aniline (200 mg, 1.12 mmol) was dissolved in 5 mL of DCM, then acetyl chloride (0.13 mL, 1.79 mmol) was added followed by the addition of triethylamine (0.25 mL, 1.79 mmol) according to the general procedure for 1 h to give the title compound (224 mg, 57%) (R_f = 0.35 (pet. ether/EtOAc 1:1, v/v)) as a white solid, mp. 117-120 ºC (lit.[6] 114-115 ºC).

IR (neat): ν_{max} 3268, 3211, 3152, 3093, 1664, 1615, 1552, 1507, 1256, 1190, 1148, 841; ^1H NMR (400 MHz, $CDCl_3$): δ 7.57 (bs, 1H), 7.52 (d, J = 9.0 Hz, 2H), 7.15 (d, J = 8.5 Hz, 2H), 2.17 (s, 3H); ^{13}C NMR (100 MHz, $CDCl_3$): δ 168.7, 145.4, 136.7, 121.8, 121.2, 120.7 (q, J = 256.5 Hz), 24.6; ^{19}F NMR (376 MHz, $CDCl_3$): δ -58.2.

General procedure for the nitration of the protected anilines

The appropriate protected aniline (1.0 equiv.) was dissolved in 95-97% sulfuric acid (4 mL) and cooled to 0 ºC. To this was added an ice-cold mixture of 95-97% sulfuric acid (4 mL) and 65 % nitric acid (5.2 mL) dropwise

over 15 min. The reaction mixture was stirred at 0 ºC for 1 h, poured over ice, and extracted with DCM (3 x 15 mL) and the combined organic phases were dried with $MgSO_4$, filtered, and concentrated under reduced pressure.[3] It was not possible to purify the products by silica-gel flash chromatography since the reaction gives two nitrated isomers, which retention factors are too narrow, then the next step was performed without further purification.

General procedure for deprotection of the anilines

The appropriate nitrated protected isomers (1 equiv.) were dissolved in MeOH, followed by the addition of HCl 37%. The mixture was refluxed overnight, then concentrated under reduced pressure. Water was added to the mixture then extracted with DCM (3 x 30 mL), and the combined organic phases were dried with $MgSO_4$, filtered, and evaporated onto celite and purified by silica-gel flash chromatography.

Synthesis of 4-fluoro-2-nitroaniline

The mixture of nitrated isomers (270 mg) was dissolved in 16 mL of methanol, then 37% hydrochloric acid (1.9 mL) was added, and the mixture was refluxed according to the general procedure for 17 h to give the title compound (132 mg, 53% from the corresponding protected aniline) (R_f = 0.20 (pet. ether/EtOAc 9:1, v/v) as a dark orange solid, mp 92-95 ºC (lit.[7] 105-106 ºC).

IR (neat): ν_{max} 3479, 3354, 3190, 3102, 2922, 2852, 1647, 1578, 1513, 1245, 1191; ^{1}H NMR (400 MHz, DMSO-d_6): δ 7.73 (dd, *J* = 9.6 Hz, 3.0 Hz, 1H), 7.44-7.38 (m, 3H), 7.06 (dd, *J* = 9.4 Hz, 5.0 Hz, 1H); ^{13}C NMR (100 MHz, DMSO-d_6): δ 151.7 (d, *J* = 231.6 Hz), 143.5, 128.6 (d, *J* = 8.8 Hz), 125.0 (d, *J* = 24.6 Hz), 121.0 (d, *J* = 7.1 Hz), 109.8 (d, *J* = 26.4 Hz).

Synthesis of 2-fluoro-6-nitroaniline and 2-fluoro-4-nitroaniline

The mixture of nitrated isomers (290 mg) was dissolved in 20 mL of MeOH, then 37% hydrochloric acid (2.3 mL) was added and the mixture was refluxed according to the general procedure for 16 h to yield the products (2-fluoro-6-nitroaniline R_f = 0.50; 2-fluoro-4-nitroaniline R_f = 0.28 (pet ether/EtOAc 8:2 v/v)) as yellow solids (2-fluoro-6-nitroaniline: 180 mg, mp. 100-103 ºC (lit.[8] 77-78 ºC); 2-fluoro-4-nitroaniline: 75 mg, mp. 132-135 ºC (lit.[9] 133-133.5 ºC)).

2-fluoro-6-nitroaniline: IR (neat): ν_{max} 3447, 3363, 3090, 2922, 2851, 1615, 1585, 1500, 1347, 1217, 870; ^{1}H NMR (400 MHz, $CDCl_3$): δ 7.66 (dd, *J* = 7.6 Hz, 2.7 Hz, 1H), 7.60 (ddd, *J* = 8.8 Hz, 4.1 Hz, 2.8 Hz, 1H), 7.08

(dd, J = 10.0 Hz, 8.9 Hz, 1H), 4.05 (bs, 2H); ^{13}C NMR (100 MHz, $CDCl_3$): δ 154.9 (d, J = 250.9 Hz), 144.9, 135.6 (d, J = 14.5 Hz), 115.7 (d, J = 21.4 Hz), 114.3 (d, J = 8.1 Hz), 111.6 (d, J = 5.9 Hz); ^{19}F NMR (376 MHz, $CDCl_3$): δ -125.3.

2-fluoro-4-nitroaniline: IR (neat): ν_{max} 3447, 3398, 3080, 2475, 1614, 1522, 1479, 1315, 1212, 884, 740; ^{1}H NMR (400 MHz, CD_3OD): δ 7.89-7.84 (m, 2H), 6.83-6.79 (m, 1H); ^{13}C NMR (100 MHz, CD_3OD): δ 150.0 (d, J = 242.8 Hz), 145.3 (d, J = 13.0 Hz), 137.6 (d, J = 7.3 Hz), 123.0 (d, J_{CF} = 2.2 Hz), 114.8 (d, J = 5.0 Hz), 112.3 (d, J = 22.8 Hz); ^{19}F NMR (376 MHz, $CDCl_3$): δ -136.9. Proton from NH_2 could not be discerned.

Synthesis of 2-nitro-4-(trifluoromethoxy)aniline

The mixture of nitrated isomers (270 mg) was dissolved in 16 mL of MeOH, then 37% hydrochloric acid (1,9 mL) was added, and the reaction mixture was refluxed according to the general procedure for 17 h to give the title product (232 mg, 63% over two steps) (R_f = 0.36 (pet. ether/EtOAc 8:2, v/v with 0.5% triethylamine)) as a brown solid, mp. 60-63 ºC (lit.[10] 64-65 ºC).

IR (neat): ν_{max} 3483, 3355, 3186, 2922, 1513, 1187, 1144; ^{1}H NMR (400 MHz, $CDCl_3$): δ 8.02 (d, J = 2.5 Hz, 1H), 7.29-7.26 (m, 1H), 6.84 (d, J = 9.0 Hz, 1H), 6.14 (bs, 2H); ^{13}C NMR (100 MHz, $CDCl_3$): δ 143.5, 138.9, 131.3, 129.9, 120.6 (q, J = 257.4 Hz), 119.9, 119.0; ^{19}F NMR (376 MHz, $CDCl_3$): δ -58.8.

Growth inhibition assay

Compounds **4a-4g**, **6a-6r**, **8a**, and **8b** were tested against *Staphylococcus aureus* (ATCC 25923), *Escherichia coli* (ATCC 259233), *Enterococcus faecalis* (ATCC 29122), *Pseudomonas aeruginosa* (ATCC 27853), and *Streptococcus agalactiae* (ATCC 12386); all strains from LGC Standards (Teddington, UK). *S. aureus*, *E. coli*, and *P. aeruginosa* were grown in Muller Hinton broth (275730, Becton, Franklin Lakes, NJ, USA). *E. faecalis* and *S. agalactiae* were cultured in brain hearth infusion broth (53286, Sigma, St. Louis, MO, USA). Fresh bacterial colonies were transferred in the respective medium and incubated at 37 °C overnight. The bacterial cultures were diluted to a culture density representing the log phase and mL/well were pipetted into a 96-well microtiter plate (734-2097, NunclonTM, Thermo Scientific, Waltham, MA, USA). The final cell density was 1500-15.000 colony forming units/well. The compound was diluted in 2% (v/v) DMSO in D_2O, providing a final assay concentration of 50% of the prepared sample, since 50 mL of sample in DMSO/water were added to 50 mL bacterial culture. After adding the samples to the plates, they were incubated overnight at 37 °C and the growth was determined by measuring the optical density at λ = 600 nm (OD600) with a 1420 Multilabel Counter

VICTOR3TM (Perkin Elmer, Waltham, MA, USA). A water sample was used as a reference control, growth medium without bacteria was used as a negative control and dilution series of Gentamycin (A2712, Merck, Darmstadt, DE) from 32 to 0.01 mg/mL was used as positive control and visually inspected for bacterial growth. The positive control was used as a system suitability test and the results of the antimicrobial assay were only considered valid when positive control was passed. The final concentration of DMSO in the assays was ≤ 2% (v/v) and was known to have no effect in the tested bacteria. The data was processed using GraphPad Prism 8.

Cytotoxicity assay

The antiproliferative activities of compounds **4a**-**4g**, **6a**-**6r**, **8a**, and **8b** were evaluated against the melanoma cell line, the hepatocellular carcinoma cell line HepG2 (ATCC, HB-8065™), and the non-malignant lung fibroblast cell line MRC5 (ATCC, CCL-171™) in an MTS *in vitro* cell proliferation assay. The compounds were tested in concentrations from 1.56-100 µM against both cell lines. HepG2 was cultured and assayed in MEM Earle's (F0325, Biochrom) supplemented with 5 mL non-essential amino acids (K0293, Biochrom) and 1 mM sodium pyruvate (L0473, Biochrom). MRC5 was cultured and assayed in MEM Eagle (M7278, Sigma-Aldrich) supplemented with 5 mL non-essential amino acids, 1 mM sodium pyruvate and 0.15% (w/v) sodium bicarbonate (L1713, Biochrom). In addition, all media were supplemented with 10% fetal bovine serum (FBS, S1810, Biowest), 10 µg/mL gentamicin (A2712, Biochrom) and 5 mL glutamine stable (200 mM per 500 mL medium, X0551, Biowest). Briefly, the cells were seeded in 96-well microtiter plates (Nunclon Delta Surface, VWR) at 4000 cells/well for MRC5 and 20,000 cells/well for HepG2. After incubation for 24 h in 5% CO_2 at 37 °C, the media was replaced, and compound added generating a total volume of 100 µL/well. MRC5 were incubated for 72 h before assaying, and HepG2 for 24 h. Subsequently, 10 µL of CellTiter 96® AQueous One Solution Reagent (G358B, Promega) was added to each well and the plates were incubated for 1 h at 37 °C. Following this, the absorbance was measured at 490 nm with a Tecan Spark Multimode Microplate reader. Negative controls were cells assayed with their respective cell media, and positive controls were cells treated with 10% DMSO (D4540, Sigma-Aldrich). Percent cell survival was calculated using the equation below. The data was visualized using GraphPad Prism 8.4.2 and IC50 was calculated. The built-in ROUT method was used to detect and remove outliers from the dataset (Q = 1%).

Percent (%) cell survival: ((absorbance treated wells-absorbance positive control))/((absorbance negative control-absorbance positive control)) x 100.

References

1. G. R. Fulmer; A. J. M. Miller; N. H. Sherden; H. E. Gottlieb; A. Nudelman; B. M. Stoltz; J. E. Bercaw; K. I. Goldberg, *Organometallics*, 2010, **29**, 2176.
2. M. O. Akram; P. S. Mali and N. T. Patil, *Org. Lett*., 2017, **19**, 3075.
3. V. Eikemo; L. K. Sydnes, M. O. Sydnes, *RSC Adv*. 2021, **11**, 32339.
4. V. Eikemo; B. Holmelid, L. K. Sydnes, M. O. Sydnes, *J. Org. Chem*. 2022, **87**, 8034.
5. M. S. Hemantkumar, S. M. Shruti, P. Pavankumar, M. S. Suraj, G. K. Rajesh, K. L. Kenneth, *Tetrahedron Lett*., 2019, **60**, 151159.
6. S. Rasheed, D. N. Rao, A. Siva Reddy, R. Shankar, P. Das, *RSC Adv*., 2015, **5**, 10567.
7. Z. Huang, W. Li, B. He, N. Zhang, Z.-Y. Guo, X.-H. Li, X.-J. Yang, *Heterocycles*, 2022, **104**, 1415.
8. B. Liedholm, *Acta Chem. Scand., Ser. B*, 1976, **30**, 146.
9. A. R. Katritzky, K. S. Laurenzo, *J. Org. Chem*., 1986, **51**, 5039.
10. L. M. Yagupolskii, I. I. Maletina, K. I. Petko, A. A. Moibenko, R. B. Strutinsky, S. N. Pivovar, Y. V. Tarasova, *J. Org. Pharmac. Chem*. 2008, **6**, 37.

^{1}H-NMR, ^{13}C-NMR, and ^{19}F-NMR of 1-((2-fluoro-4-nitrophenyl)amino)-3-phenylpropan-2-ol (4a)

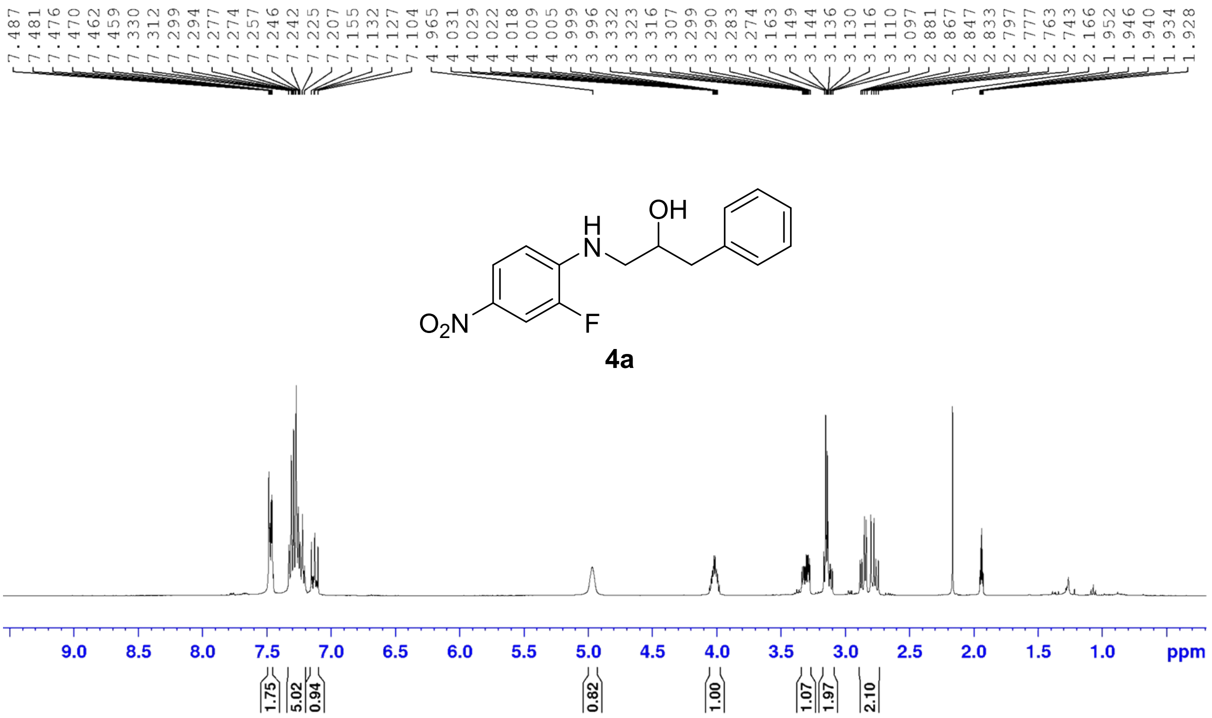

^{1}H NMR (400 MHz, CD_3CN) spectrum of compound **4a**.

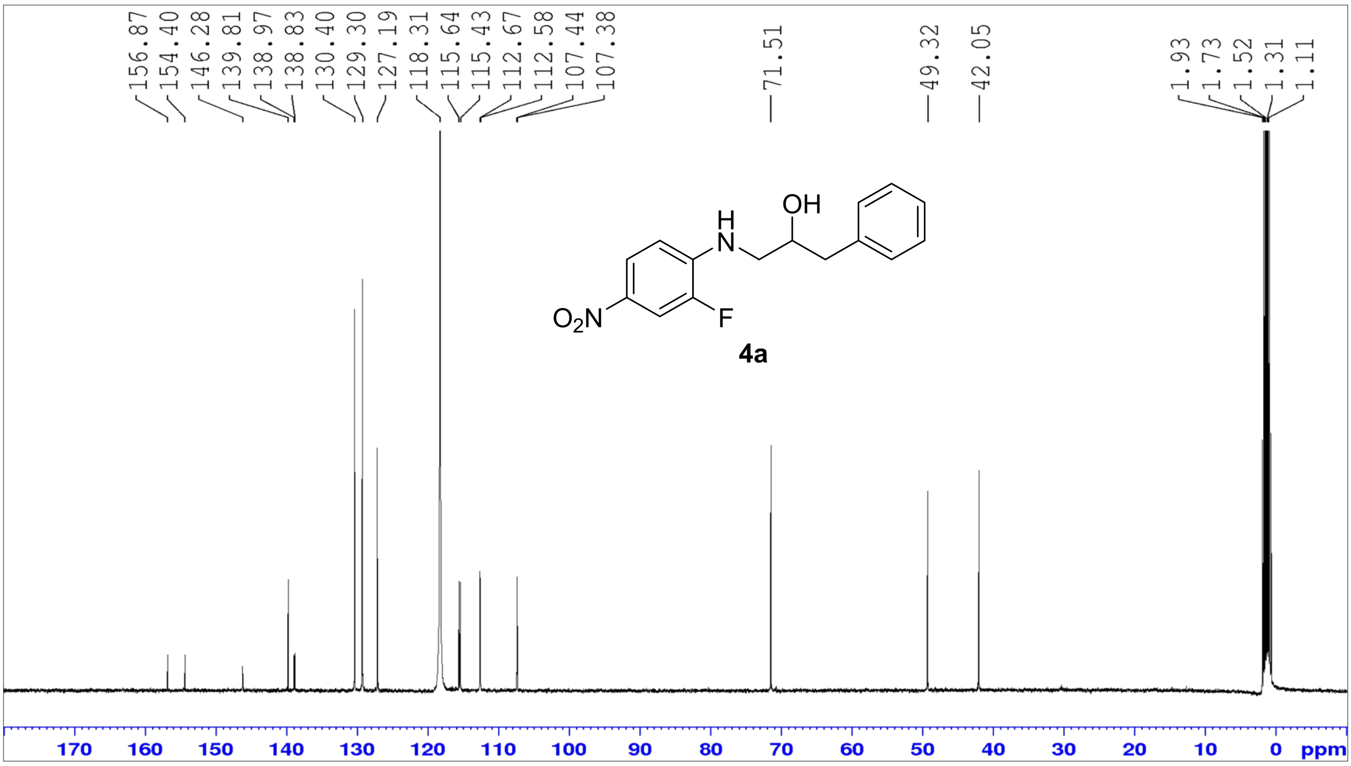

^{13}C NMR (100 MHz, CD_3CN) spectrum of compound **4a**.

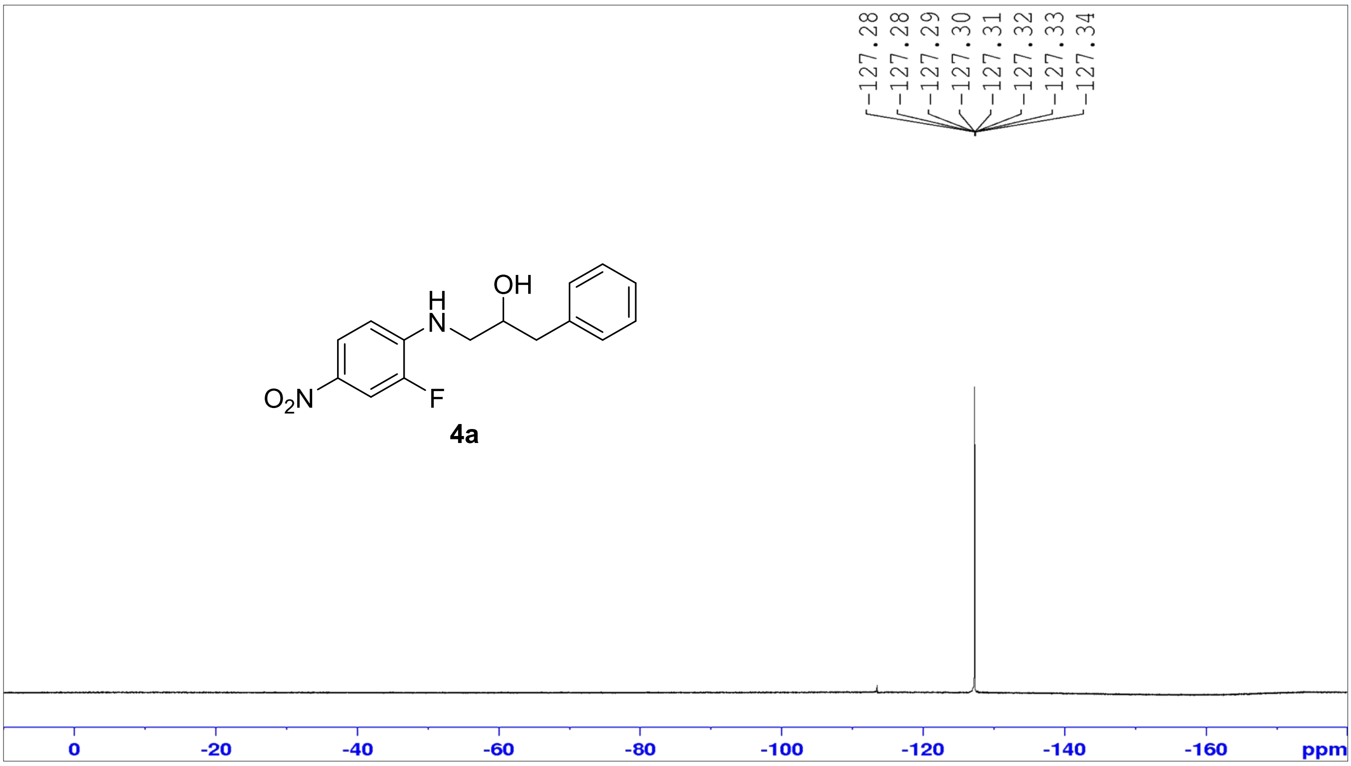

^{19}F NMR (376 MHz, CD_3CN) spectrum of compound **4a**.

^{1}H NMR and ^{13}C NMR of 1-((2-chloro-4-nitrophenyl)amino)-3-phenylpropan-2-ol (4b)

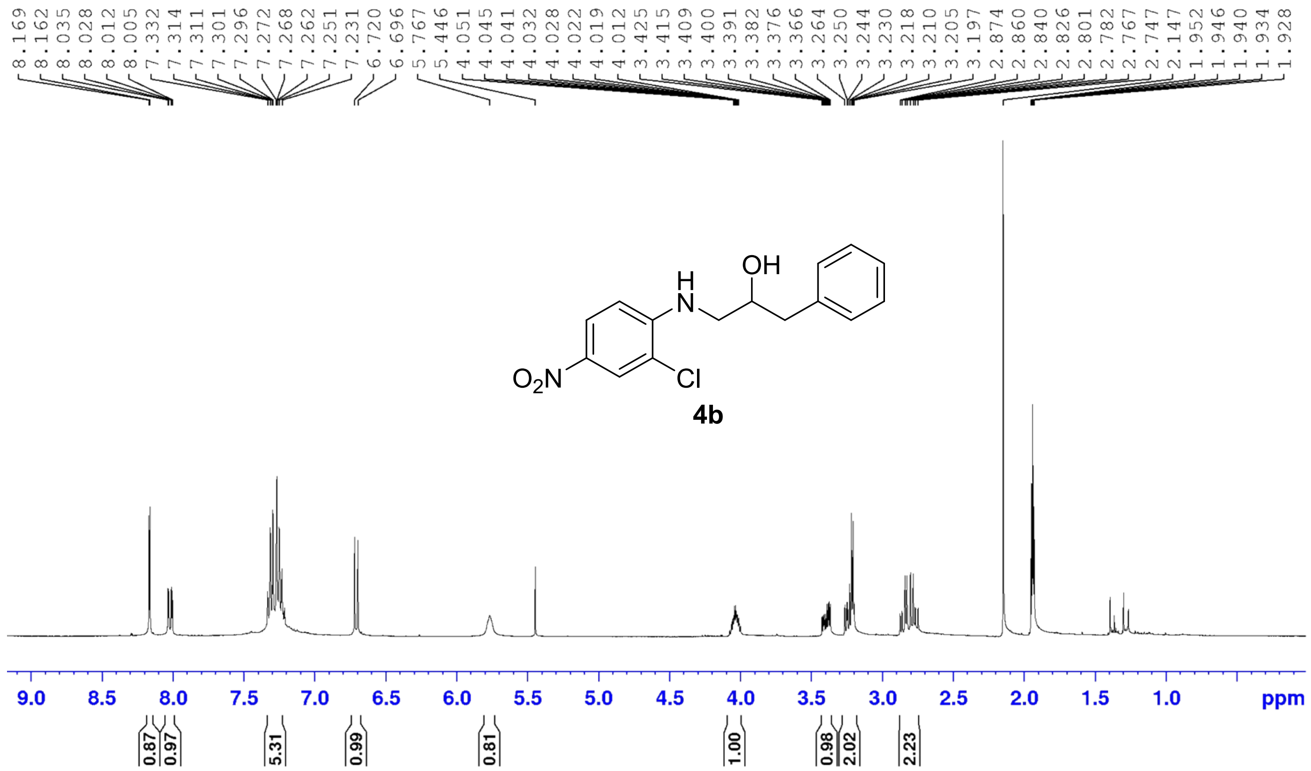

^{1}H NMR (400 MHz, CD_3CN) spectrum of compound **4b.**

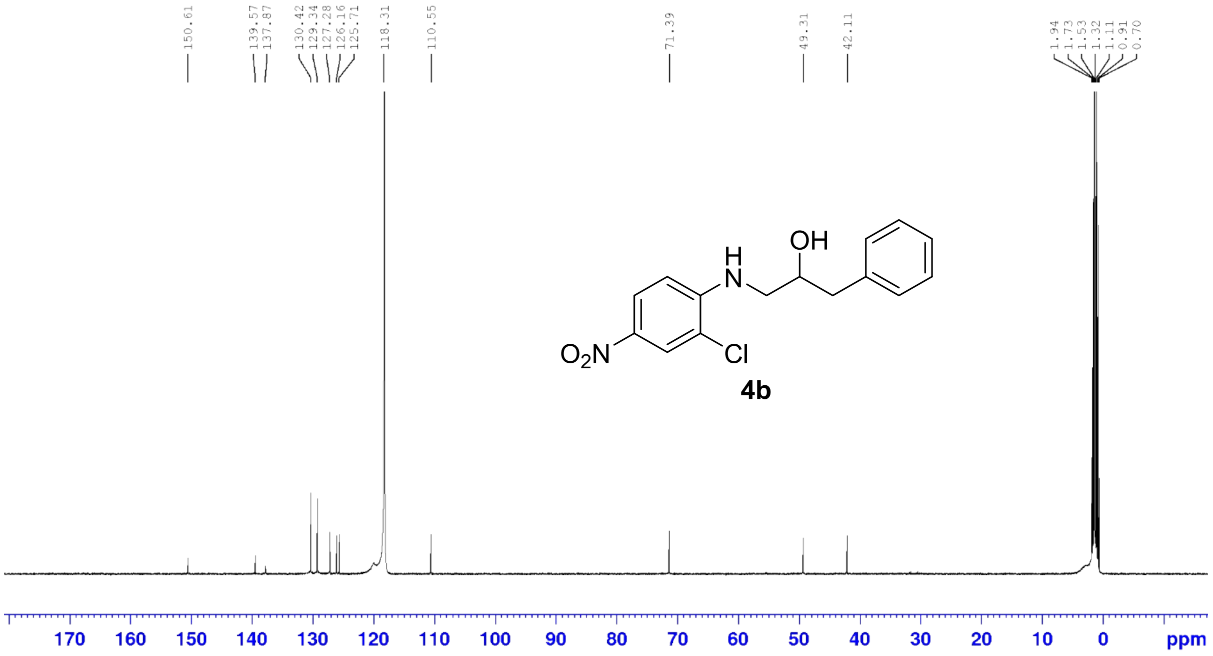

^{13}C NMR (100 MHz, CD_3CN) spectrum of compound **4b**.

^{1}H-

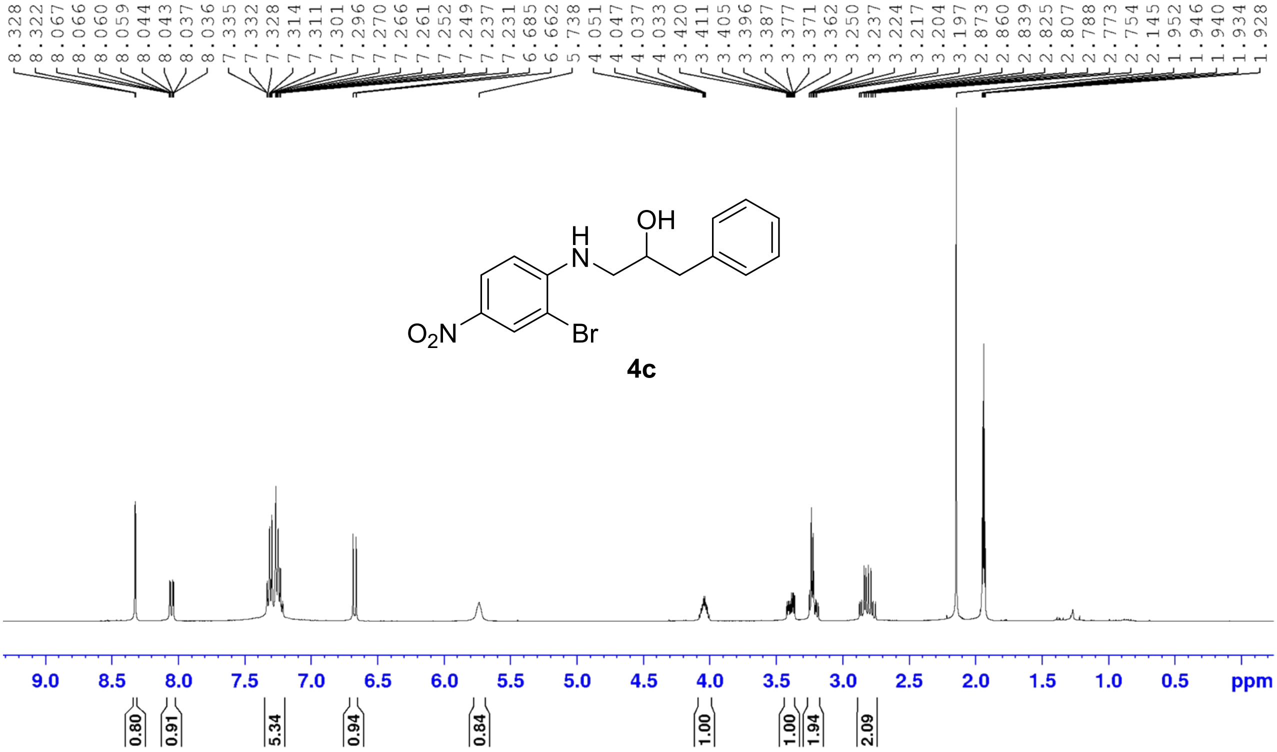

NMR, and ^{13}C-NMR of the 1-((2-chloro-4-nitrophenyl)amino)-3-phenylpropan-2-ol (4c)

^{1}H NMR (400 MHz, CD_3CN) spectrum of compound **4c**.

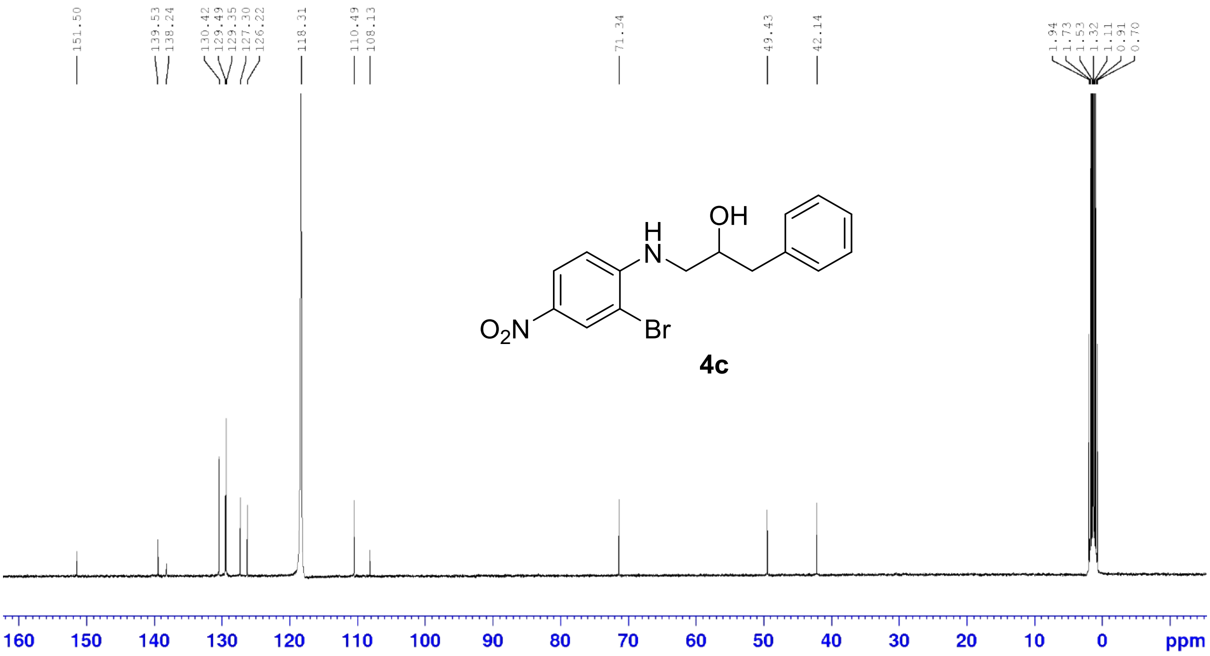

^{13}C NMR (100 MHz, CD_3CN) spectrum of compound **4c**.

^{1}H-NMR, ^{13}C-NMR and ^{19}F-NMR of 1-((4-fluoro-2-nitrophenyl)amino)-3-phenylpropan-2-ol (4d)

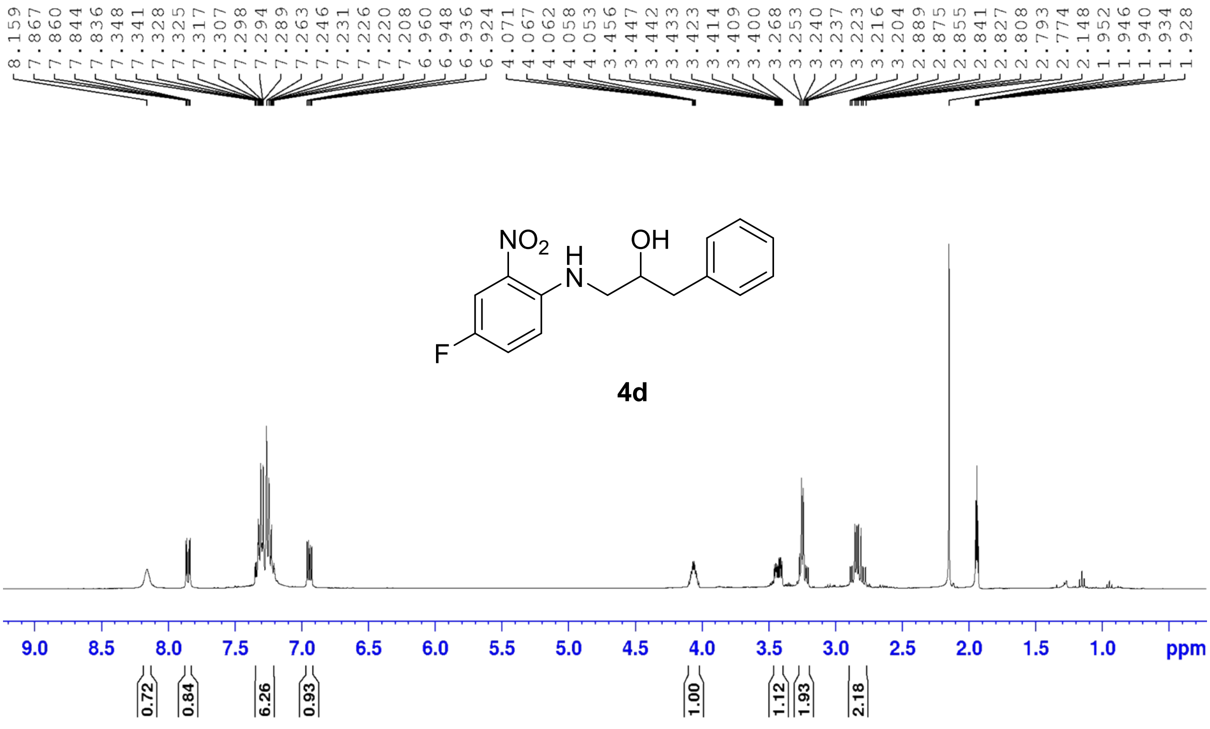

^{1}H NMR (400 MHz, CD_3CN) spectrum of compound **4d**.

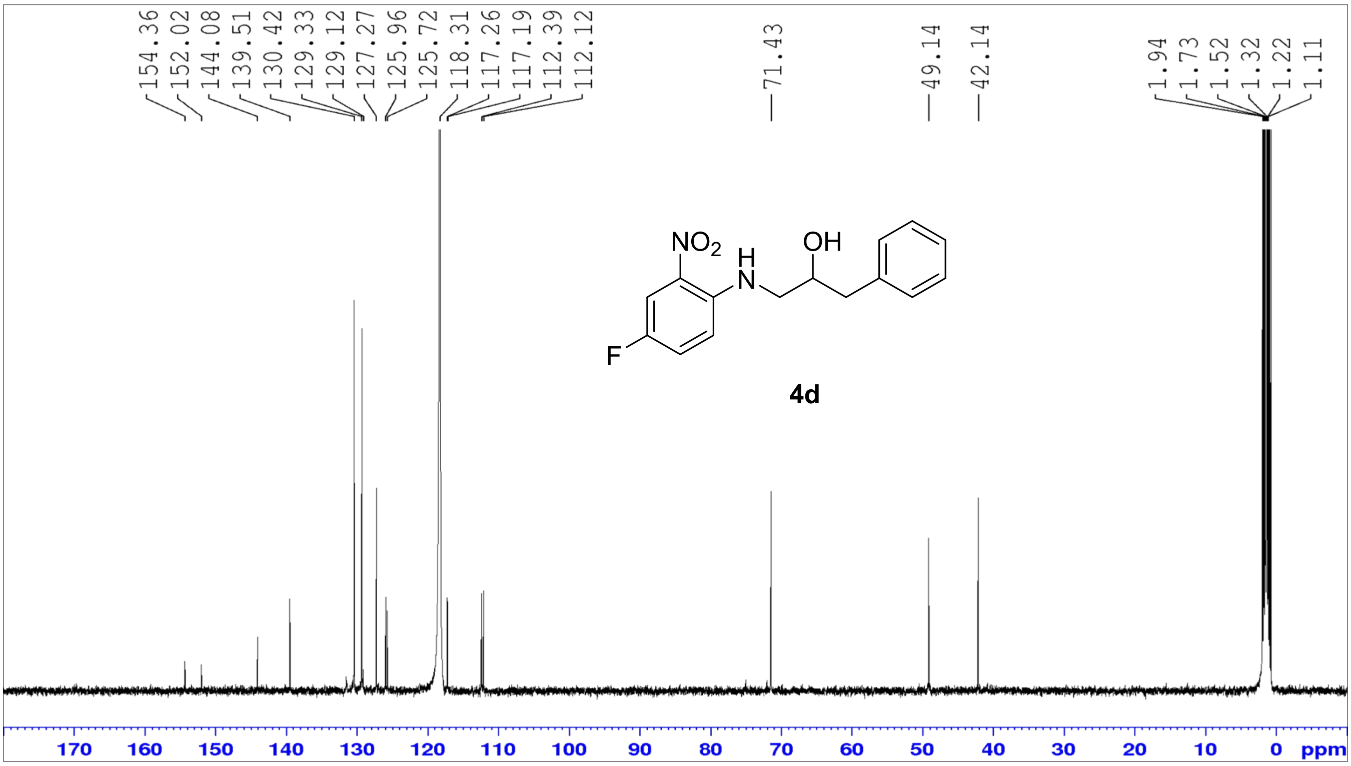

^{13}C NMR (100 MHz, CD_3CN) spectrum of compound **4d**.

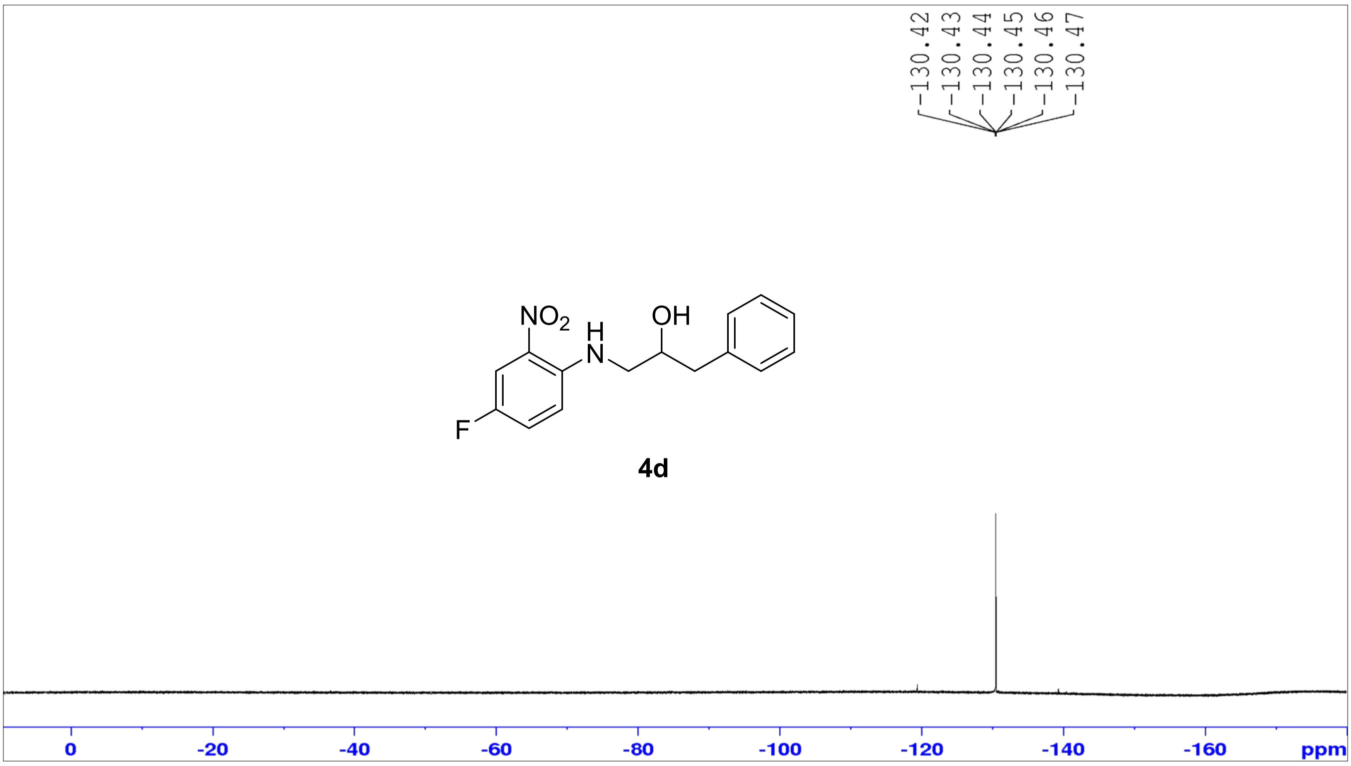

^{19}F NMR (376 MHz, CD_3CN) spectrum of compound **4d**.

^{1}H NMR, ^{19}F NMR, and ^{13}C NMR of 1-((5-Fluoro-2-nitrophenyl)amino)-3-phenylpropan-2-ol (4e)

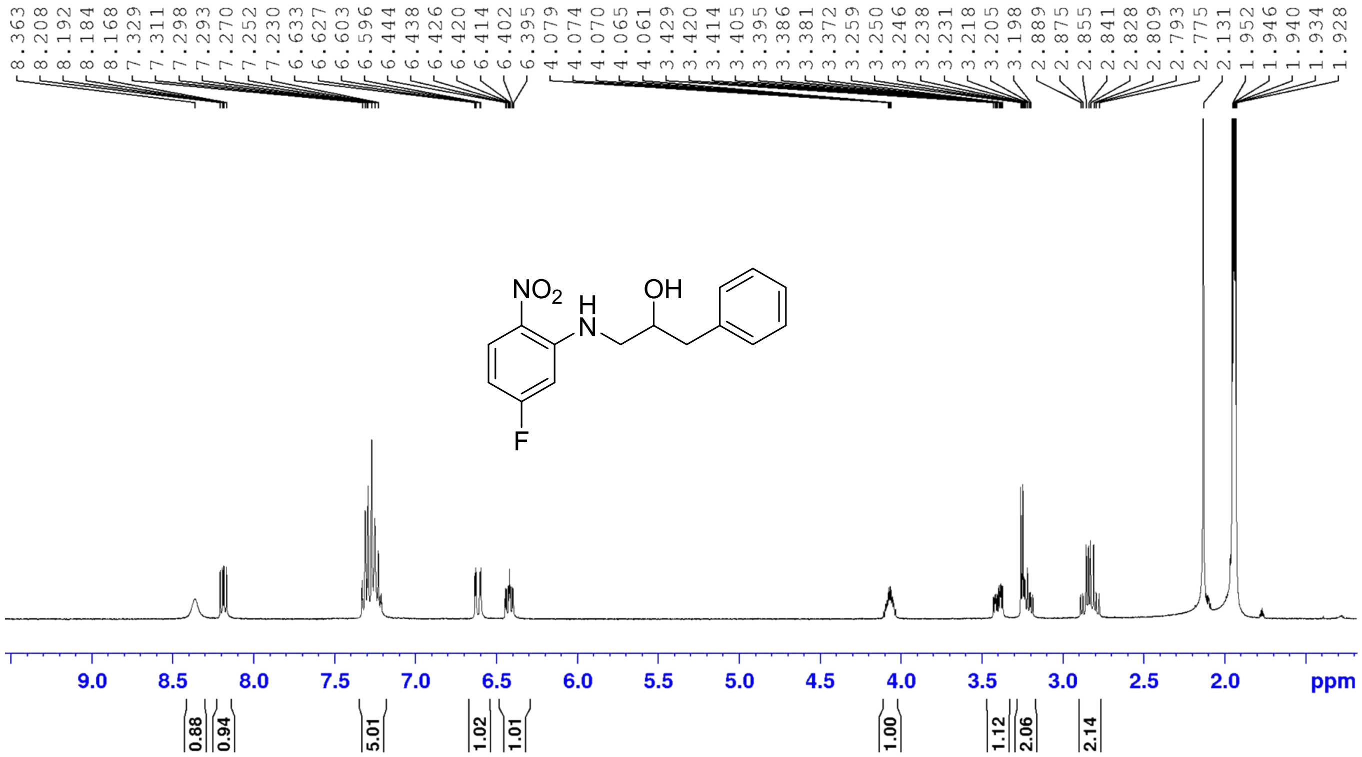

^{1}H NMR (400 MHz, CD_3CN) spectrum of compound **4e**.

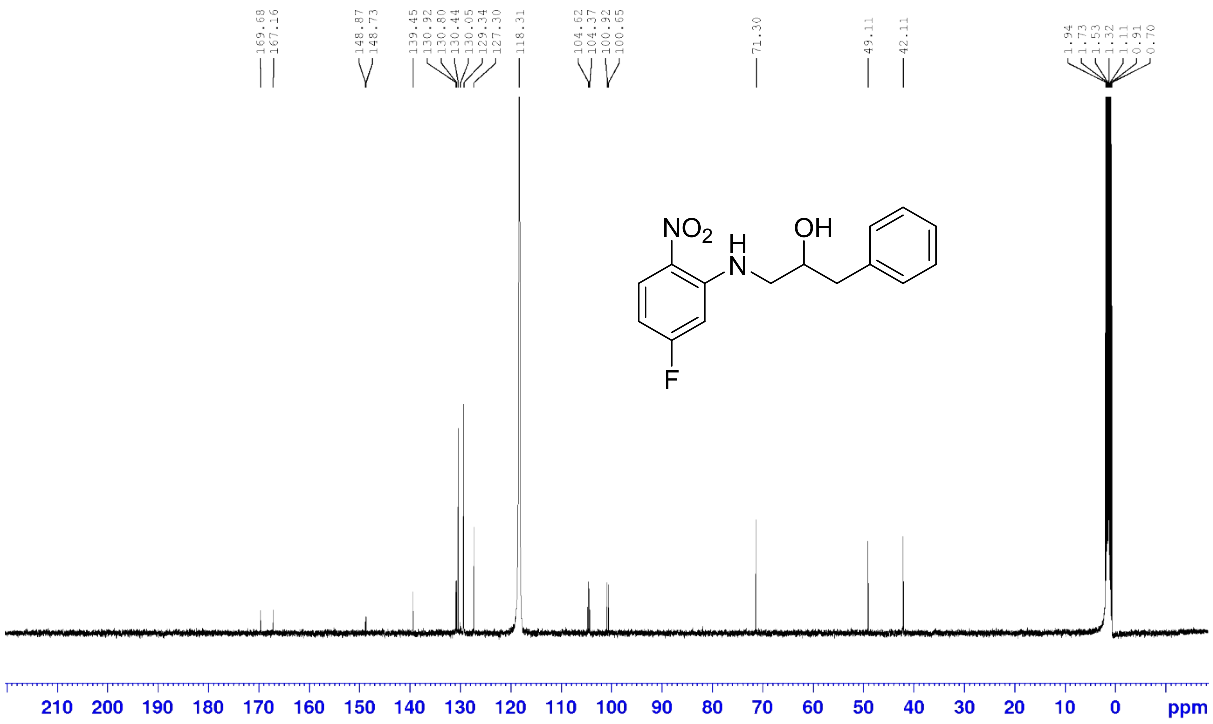

^{13}C NMR (100 MHz, CD_3CN) spectrum of compound **4e**.

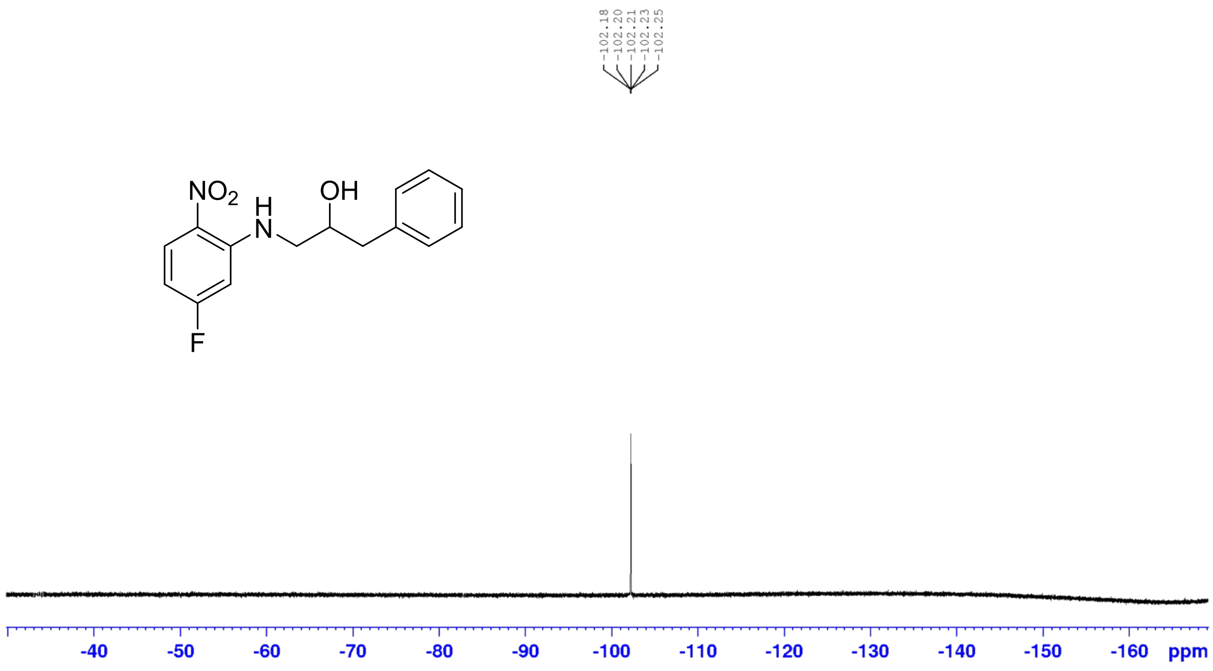

^{19}F NMR (376 MHz, CD_3CN) spectrum of compound **4e**.

^{1}H-NMR, ^{13}C-NMR, and ^{19}F-NMR of 1-((2-fluoro-6-nitrophenyl)amino)-3-phenylpropan-2-ol (4f)

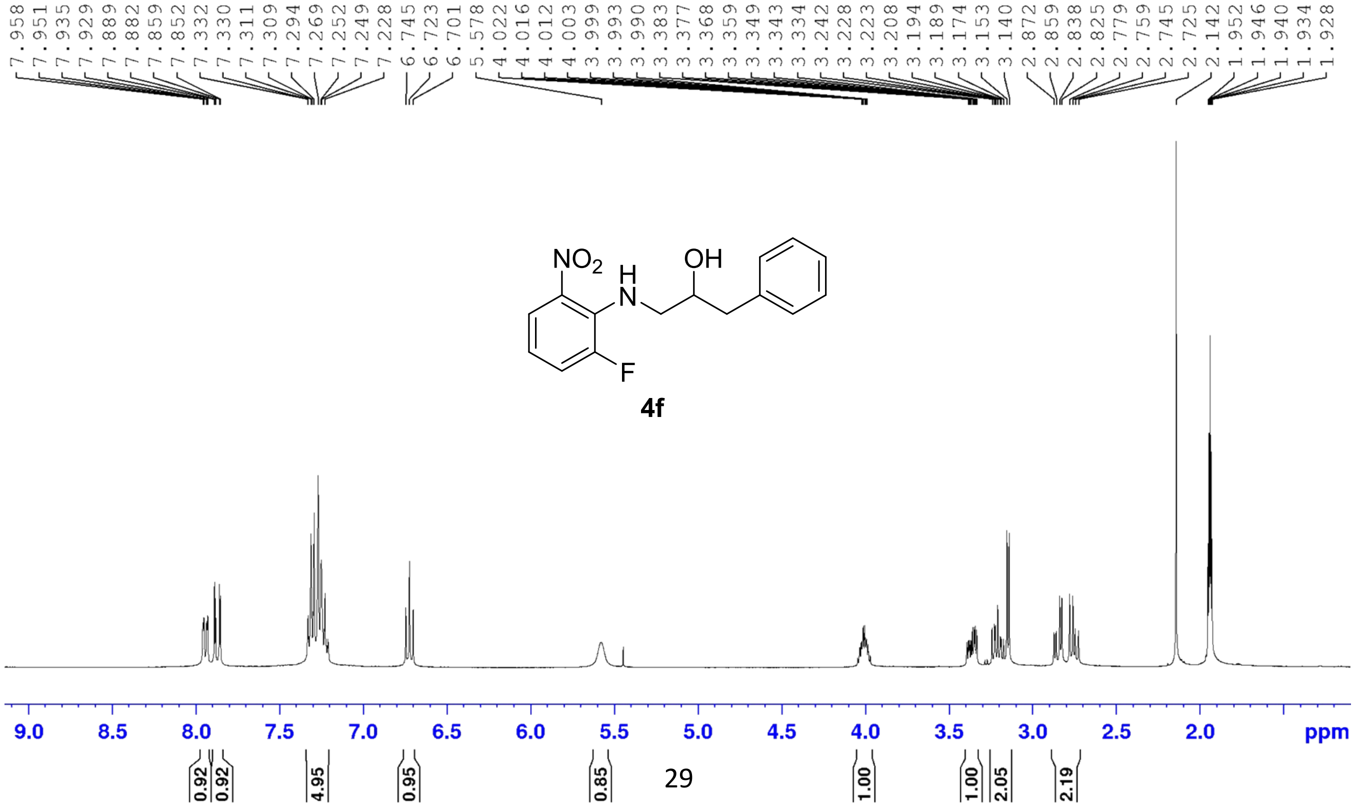

^{1}H NMR (400 MHz, CD_3CN) spectrum of compound (**4f**).

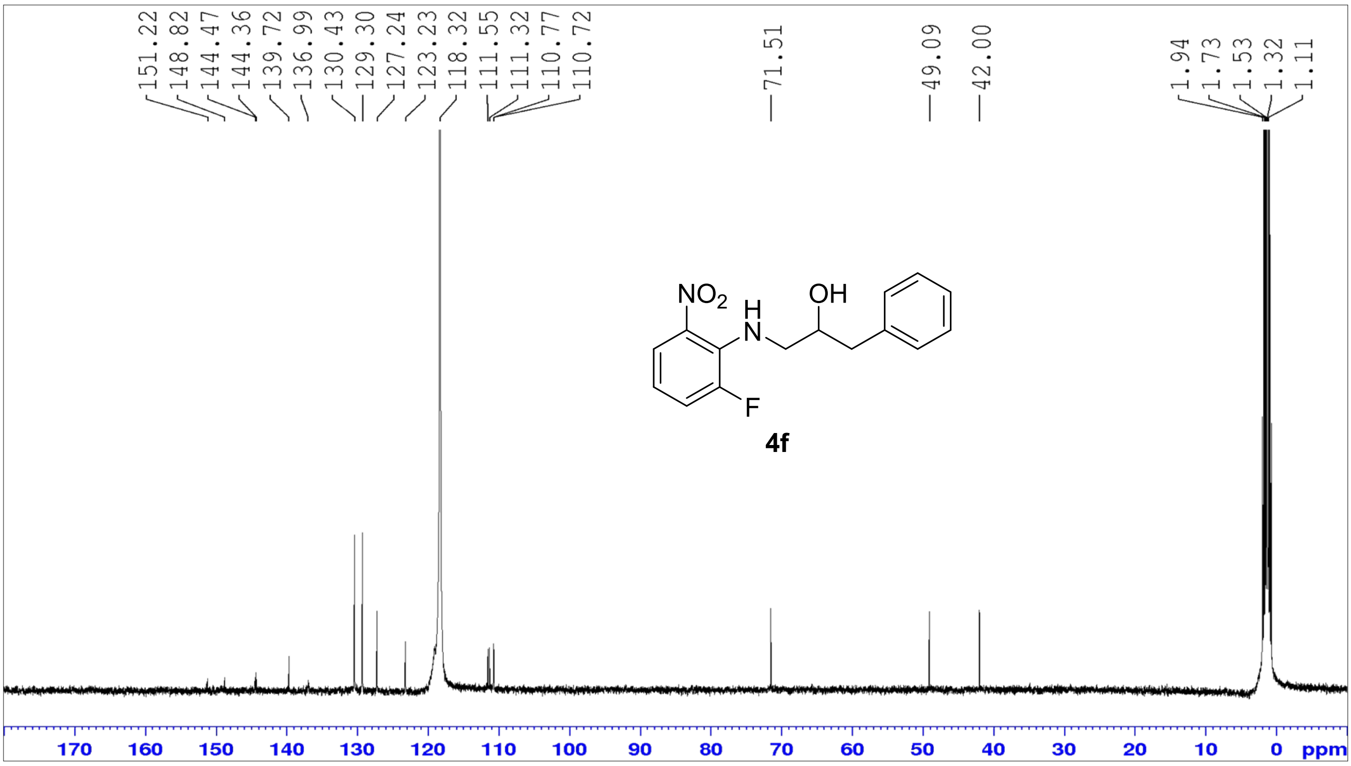

^{13}C NMR (100 MHz, CD_3CN) spectrum of compound **4f**.

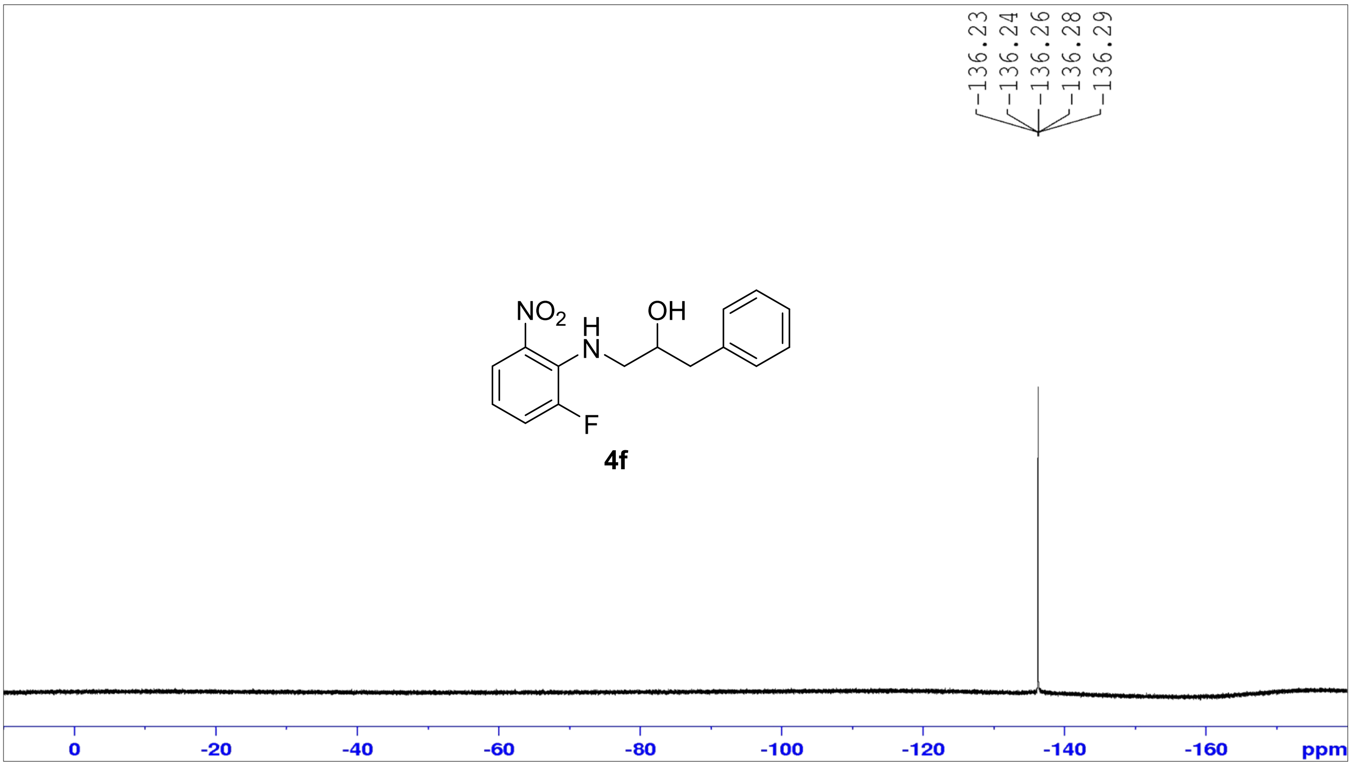

^{19}F NMR (376 MHz, CD_3CN) spectrum of compound **4f**.

^{1}H-NMR, ^{13}C-NMR, and ^{19}F-NMR of 1-((2-nitro-4-(trifluoromethoxy)phenyl)amino)-3-phenylpropan-2-ol (4g)

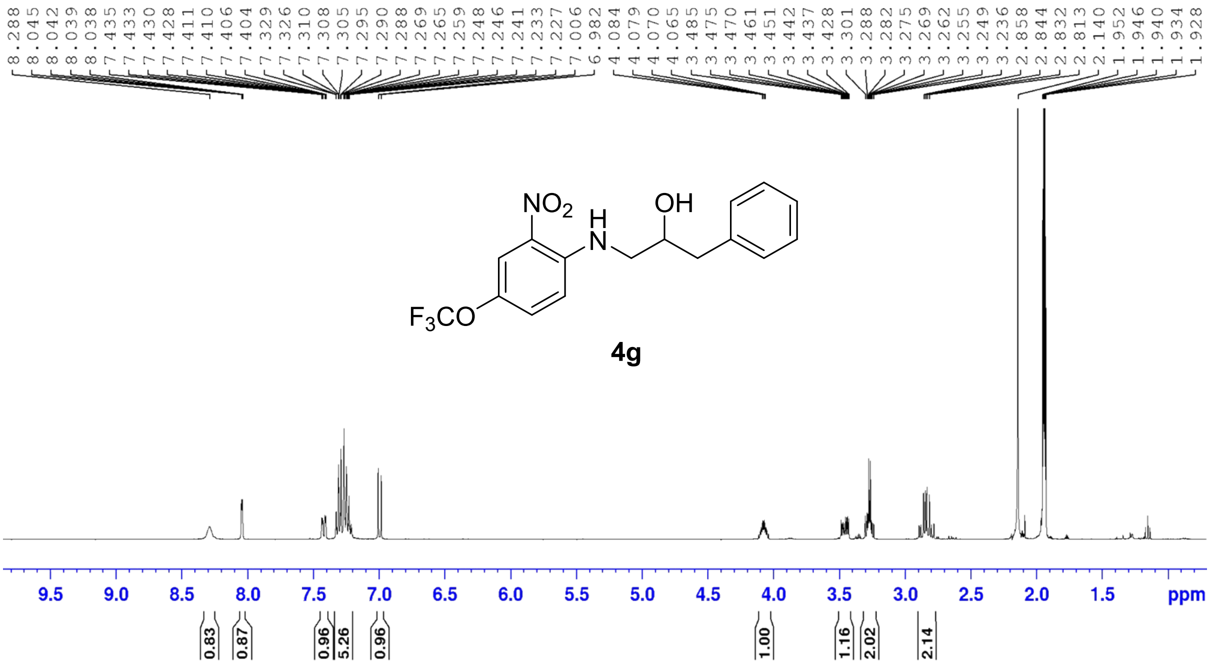

^{1}H NMR (400 MHz, CD_3CN) spectrum of compound **4g**.

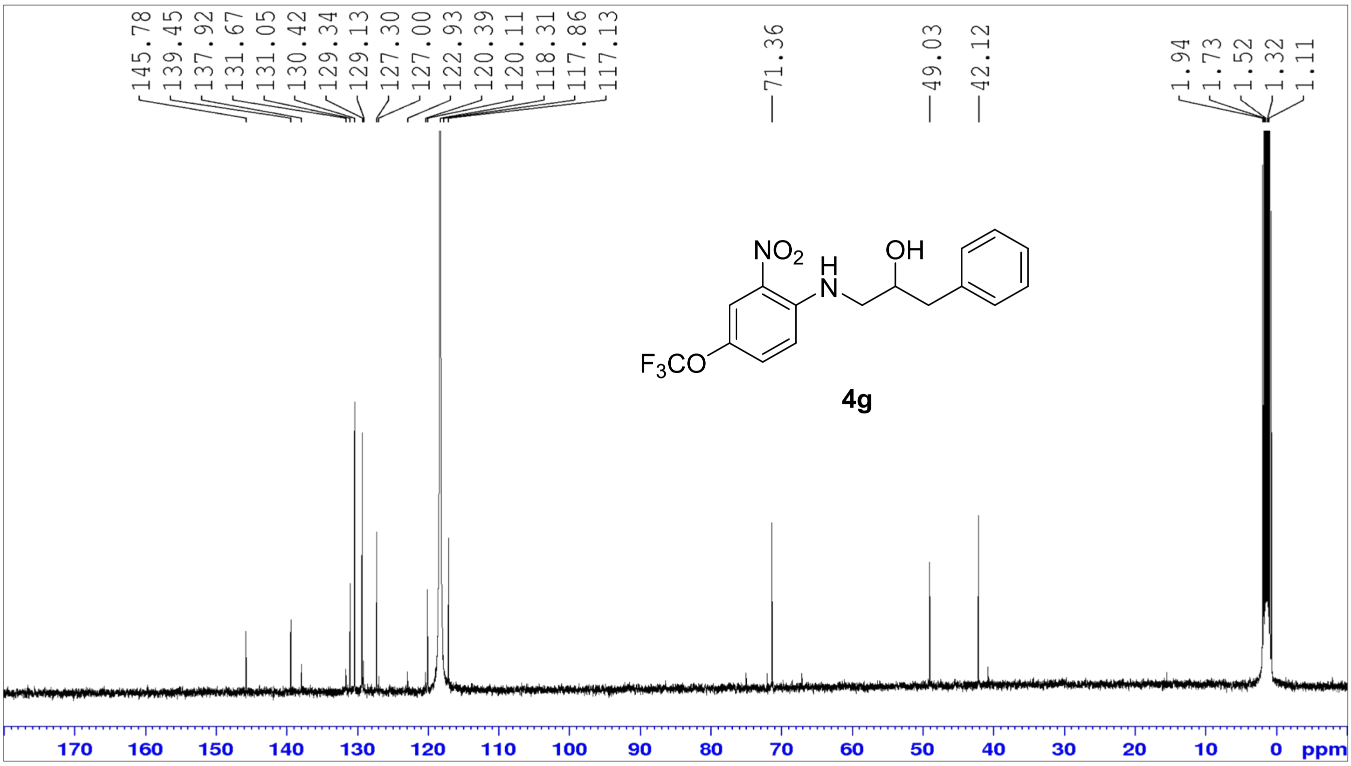

^{13}C NMR (100 MHz, CD_3CN) spectrum of compound **4g**.

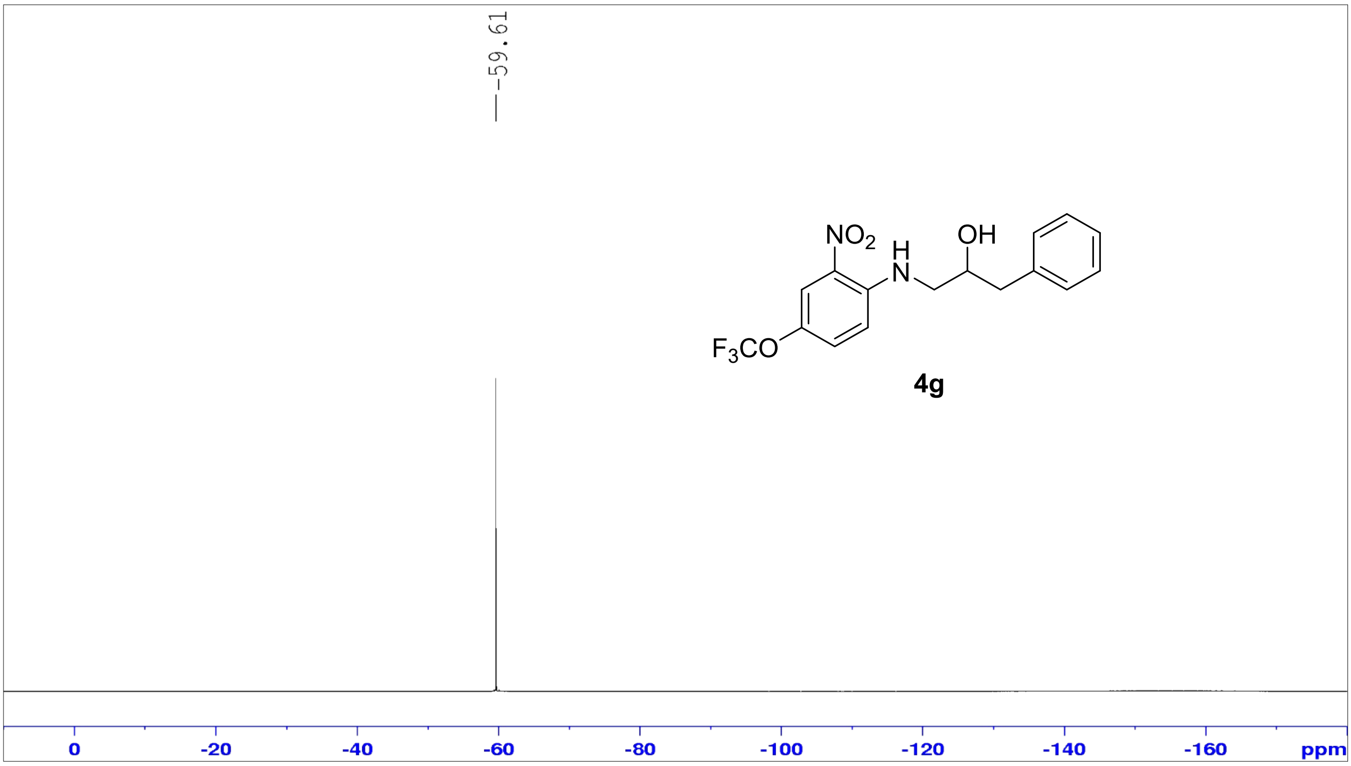

^{19}F NMR (376 MHz, CD_3CN) spectrum of compound **4g**.

^{1}H-NMR, ^{13}C-NMR, and ^{19}F-NMR spectra of 1-((4-fluorophenyl)amino)-3-(4-nitrophenyl)propan-

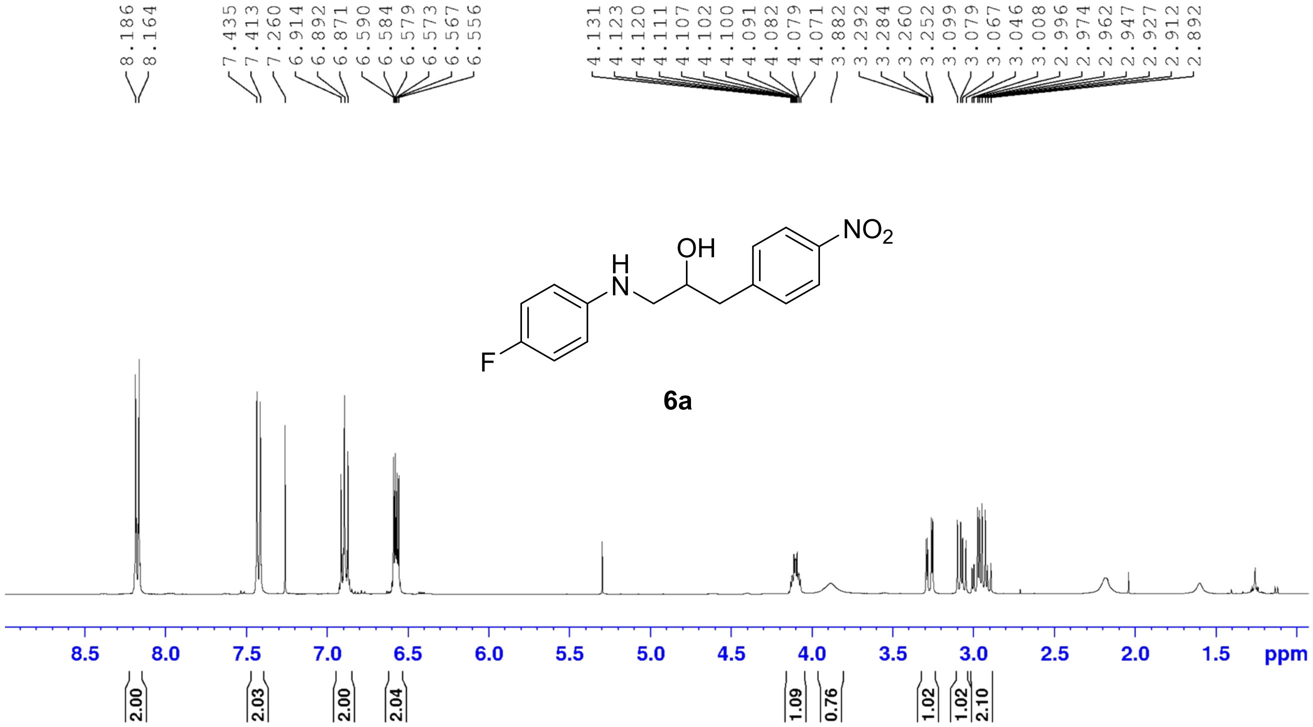

2-ol (6a).

^{1}H NMR (400 MHz, $CDCl_3$) spectrum of compound **6a**.

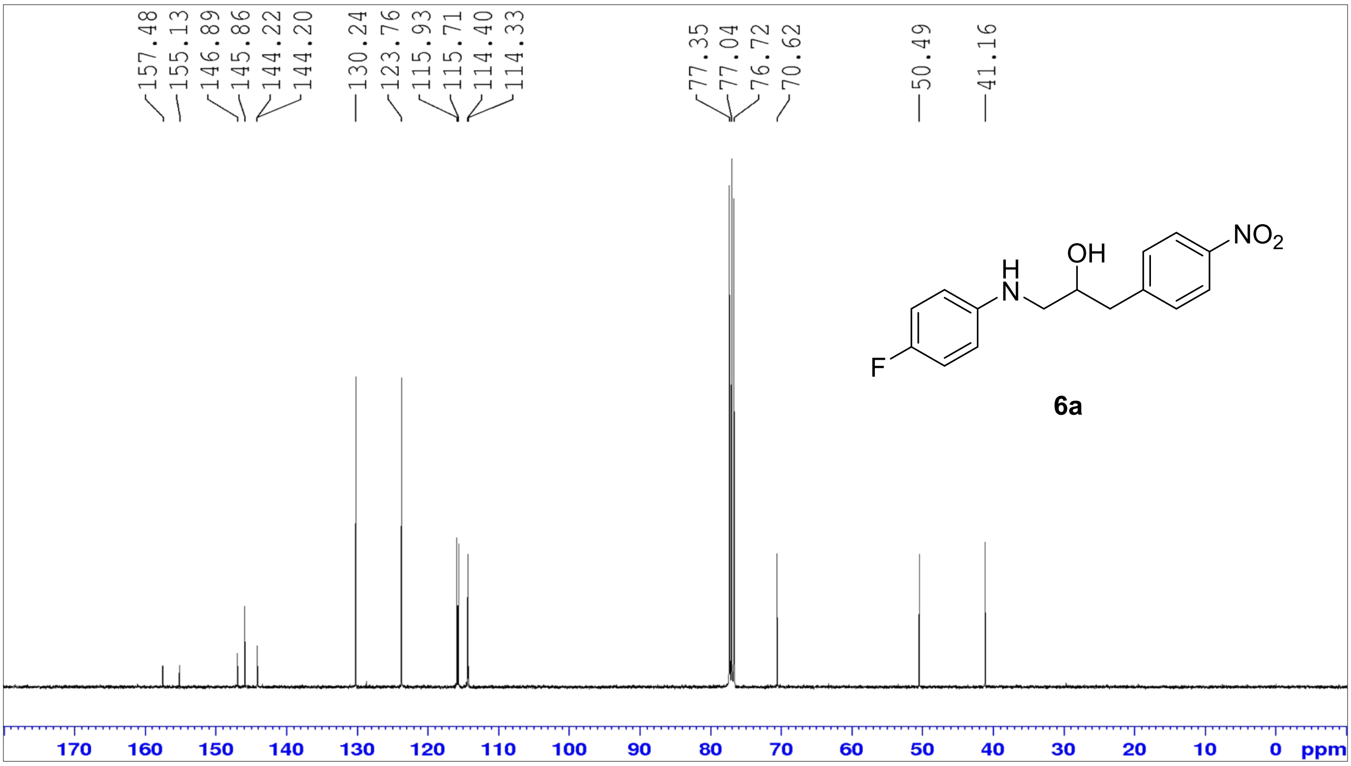

^{13}C NMR (100 MHz, $CDCl_3$) spectrum of compound **6a**.

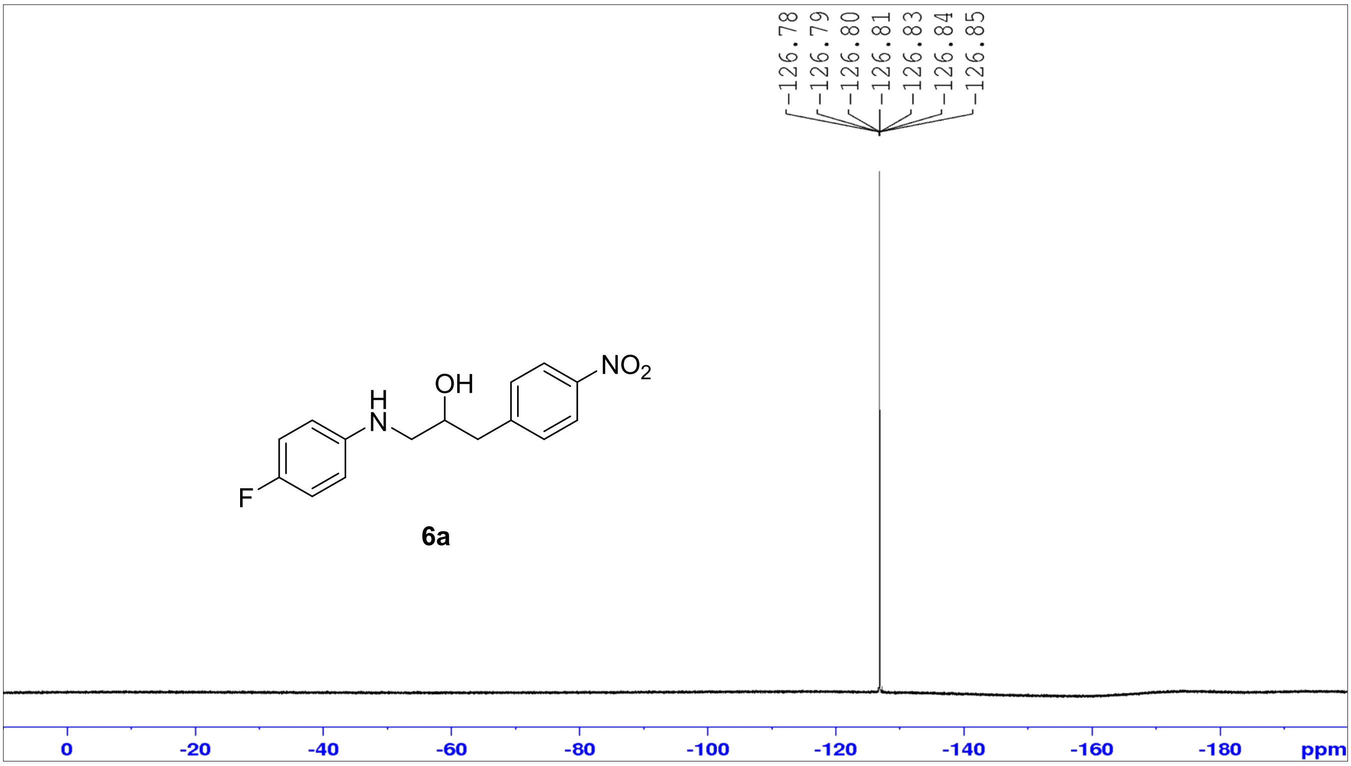

^{19}F NMR (376 MHz, $CDCl_3$) spectrum of compound **6a**.

^{1}H NMR, ^{19}F NMR, and ^{13}C NMR of 1-((3-fluorophenyl)amino)-3-(4-nitrophenyl)propan-2-ol (6b)

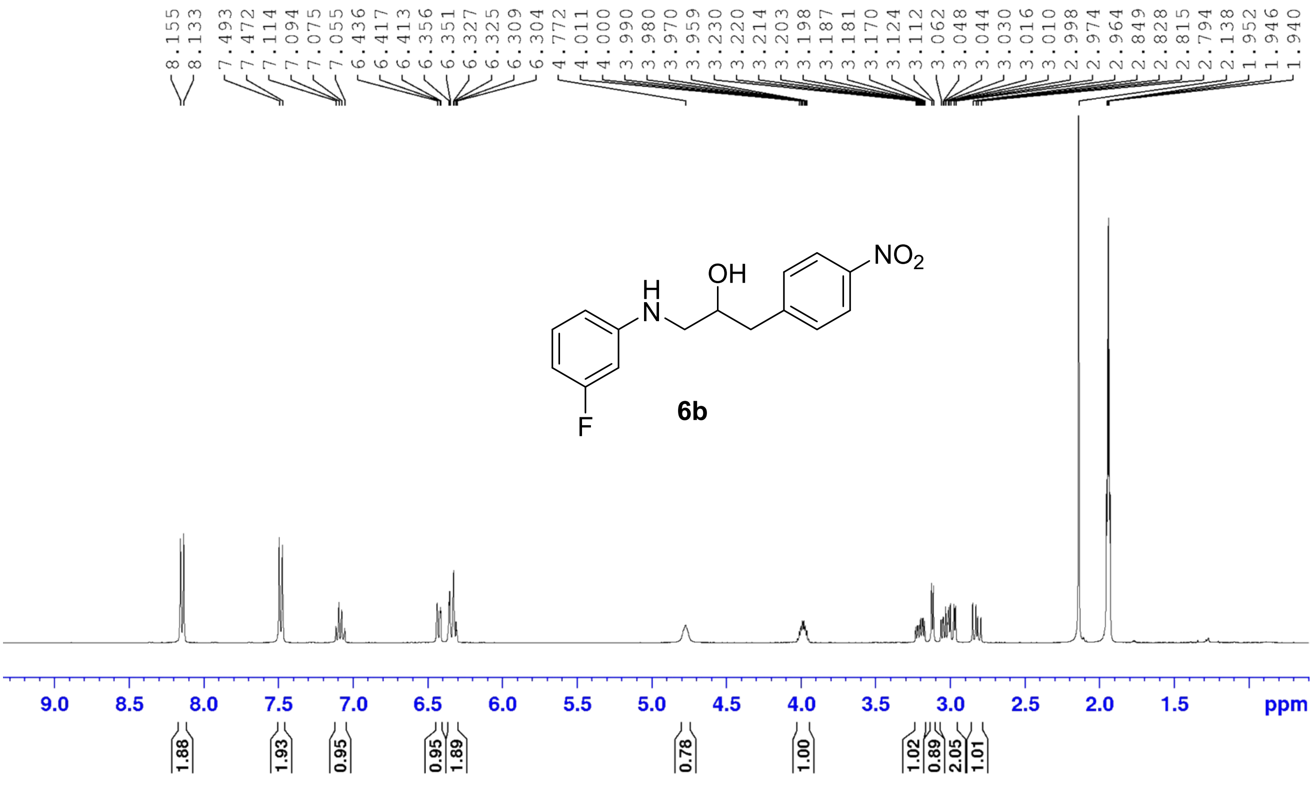

^{1}H NMR (400 MHz, CD_3CN) spectrum of compound **6b**.

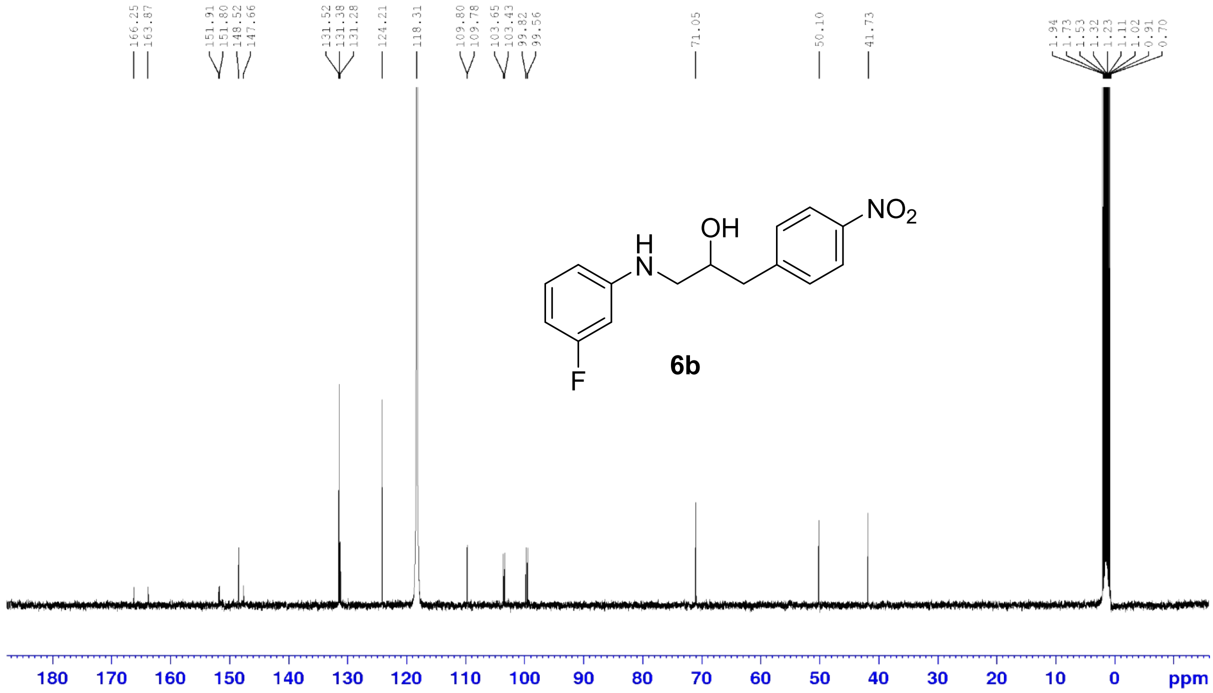

^{13}C NMR (100 MHz, CD_3CN) spectrum of compound **6b**.

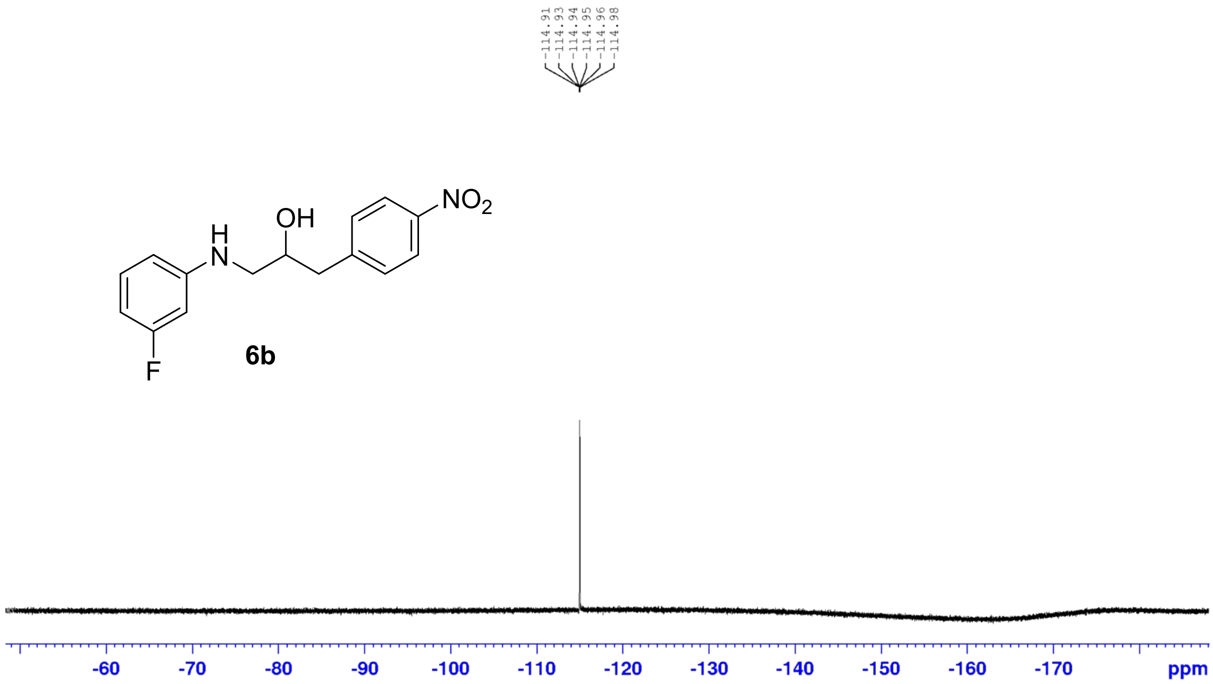

^{19}F NMR (376 MHz, CD_3CN) spectrum of compound **6b**.

^{1}H-NMR, ^{13}C-NMR and ^{19}F-NMR of 1-((4-fluoro-2-nitrophenyl)amino)-3-phenylpropan-2-ol (6c).

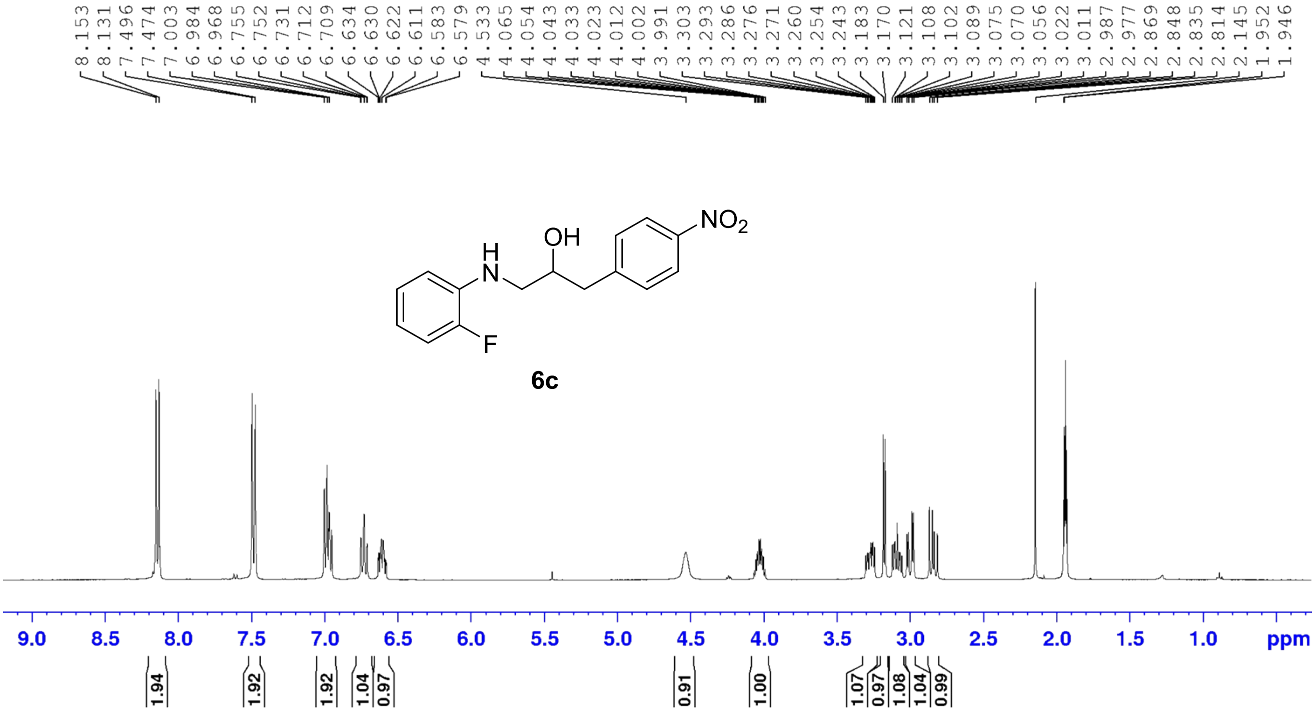

^{1}H NMR (400 MHz, CD_3CN) spectrum of compound **6c**.

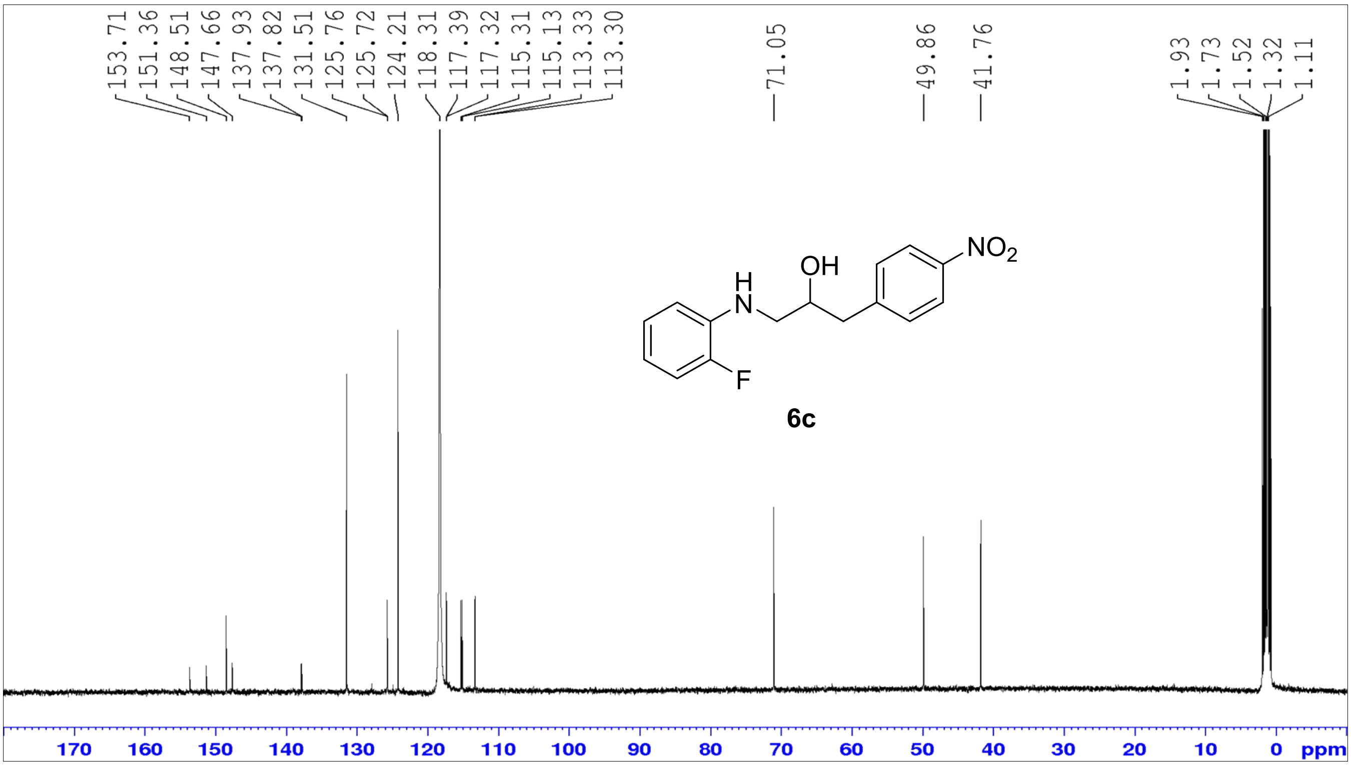

^{13}C NMR (100 MHz, CD_3CN) spectrum of compound **6c**.

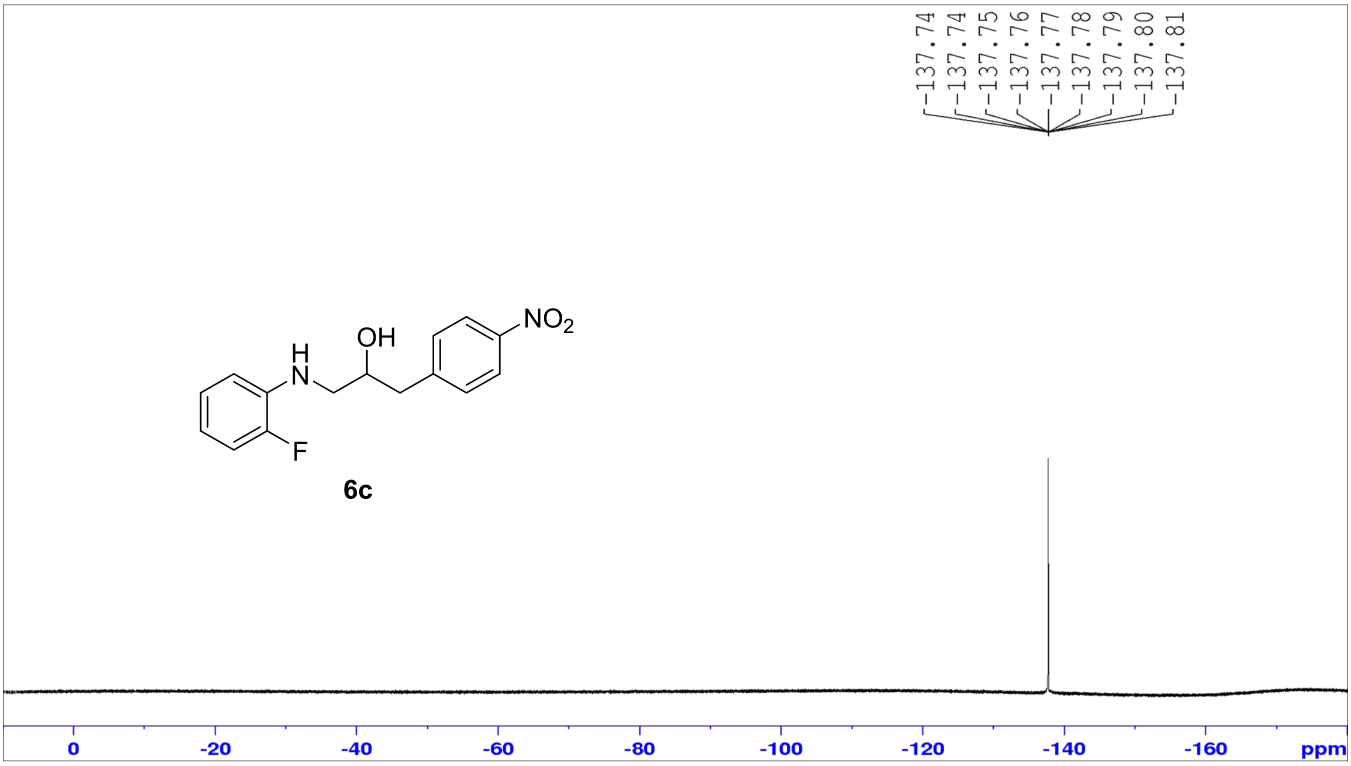

^{19}F NMR (376 MHz, CD_3CN) spectrum of compound **6c**.

^{1}H-NMR, and ^{13}C-NMR of 1-((4-bromophenyl)amino)-3-(4-nitrophenyl)propan-2-ol (6d)

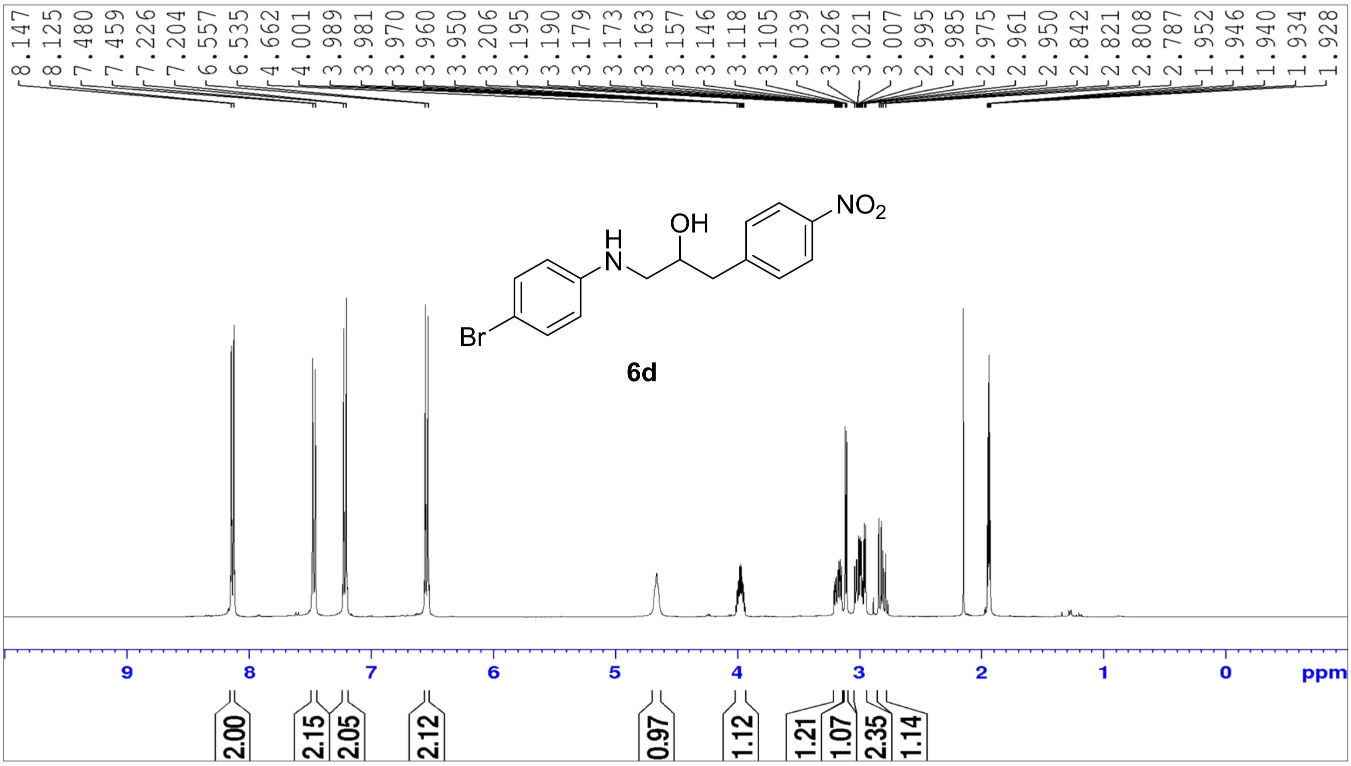

^{1}H NMR (400 MHz, CD_3CN) spectrum of compound **6d**.

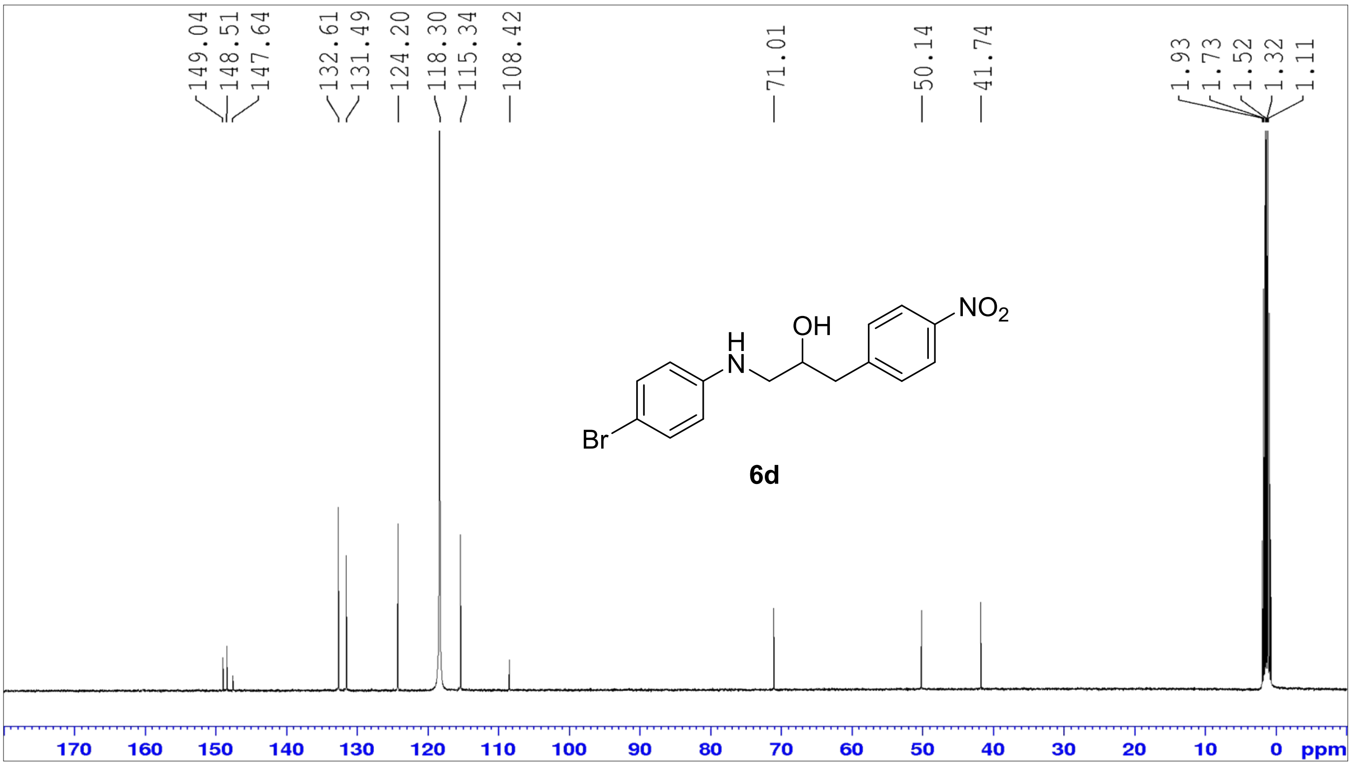

^{13}C NMR (100 MHz, CD_3CN) spectrum of compound **6d**.

^{1}H-NMR, and ^{13}C-NMR of 1-((3-bromophenyl)amino)-3-(4-nitrophenyl)propan-2-ol (6e)

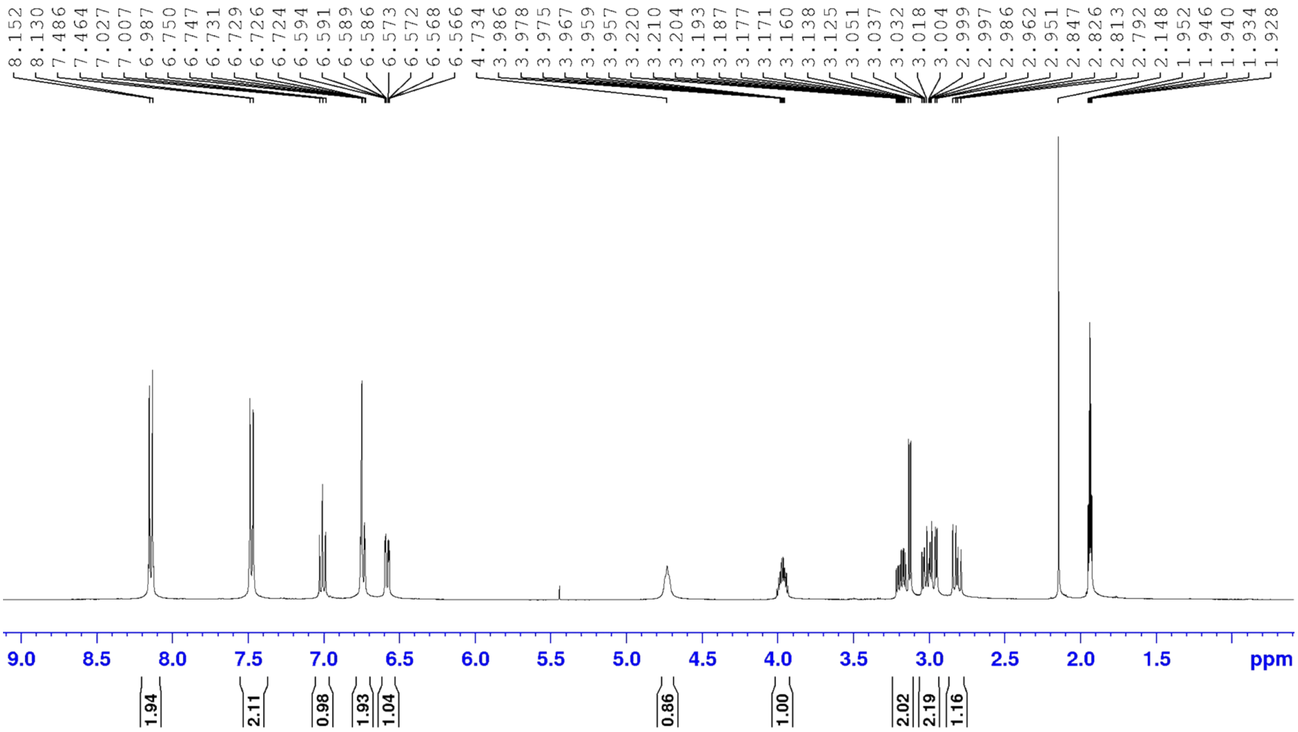

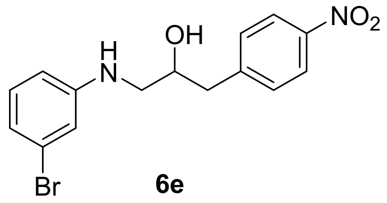

^{1}H NMR (400 MHz, CD_3CN) spectrum of compound **6e**.

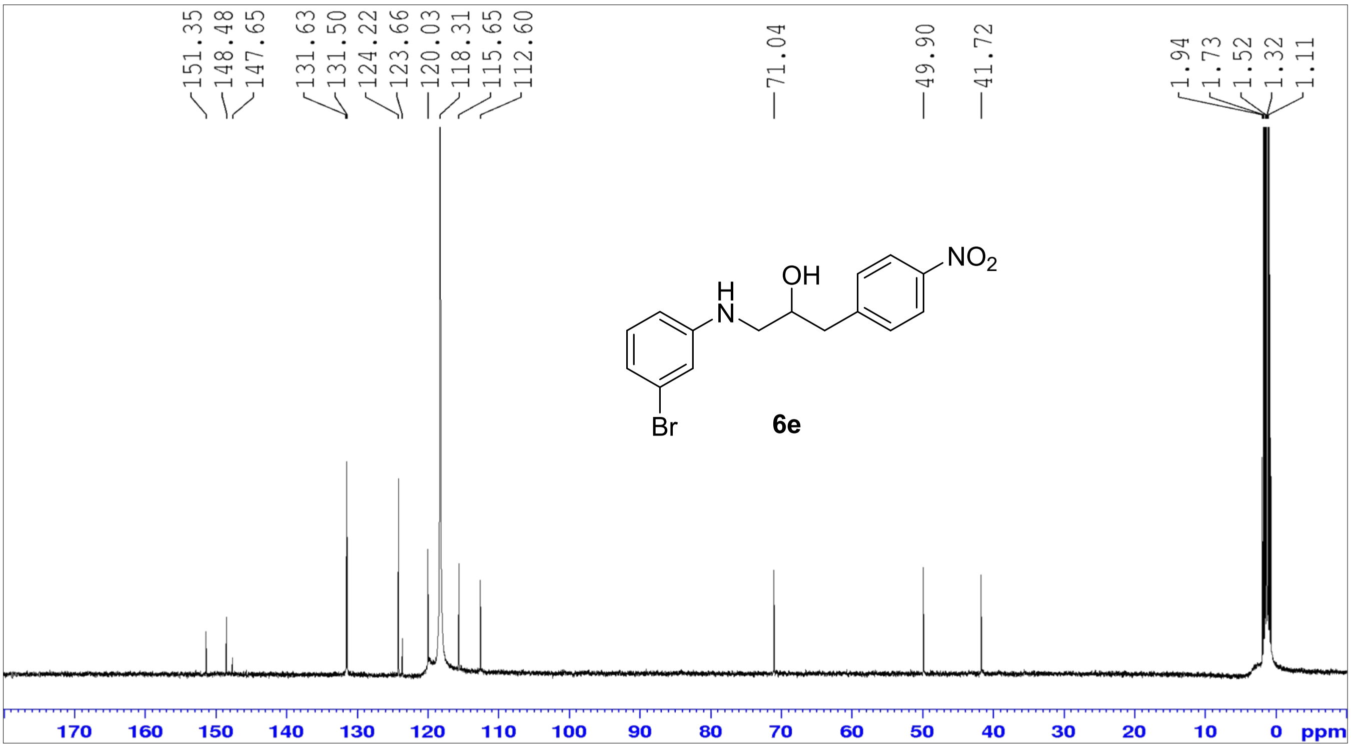

^{13}C NMR (100 MHz, CD_3CN) spectrum of compound **6e**.

^{1}H-NMR, and ^{13}C-NMR of 1-((2-bromophenyl)amino)-3-(4-nitrophenyl)propan-2-ol (6f)

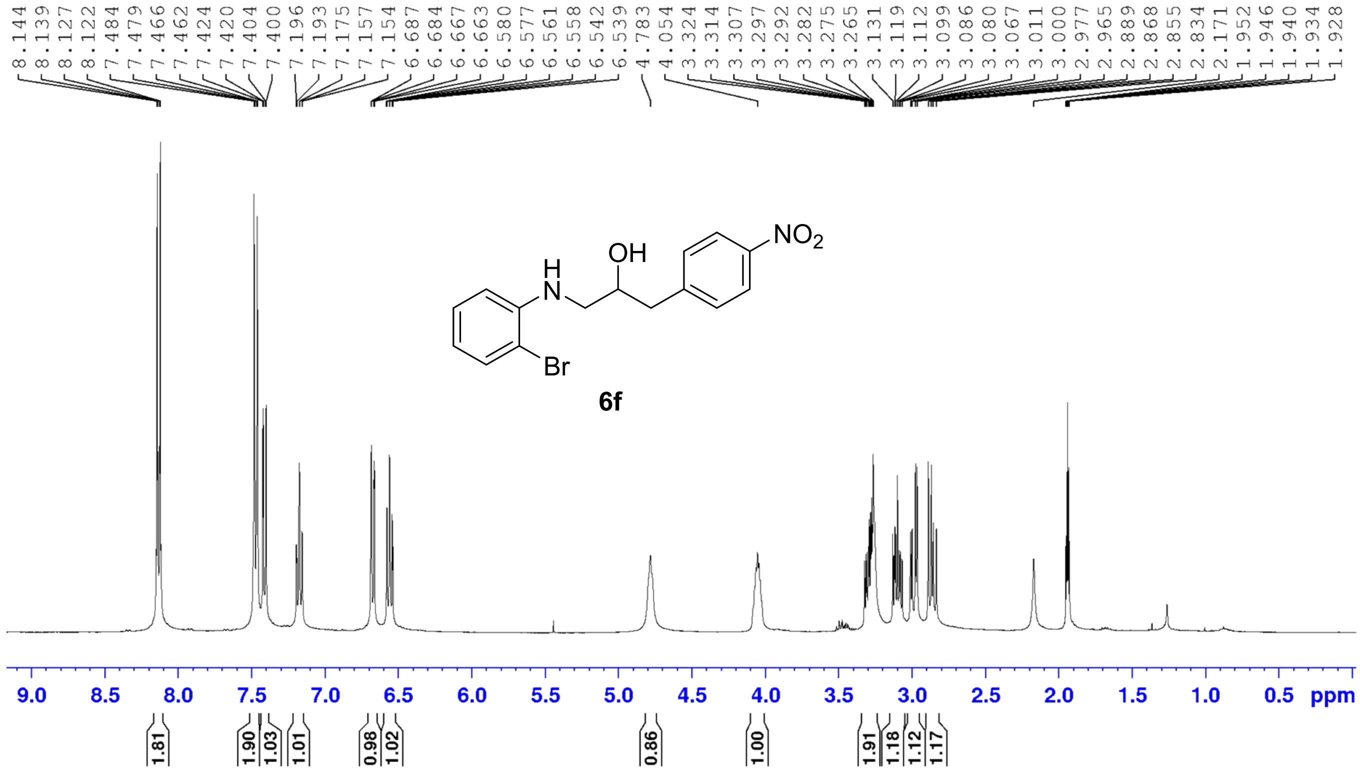

^{1}H NMR (400 MHz, CD_3CN) spectrum of compound **6f**.

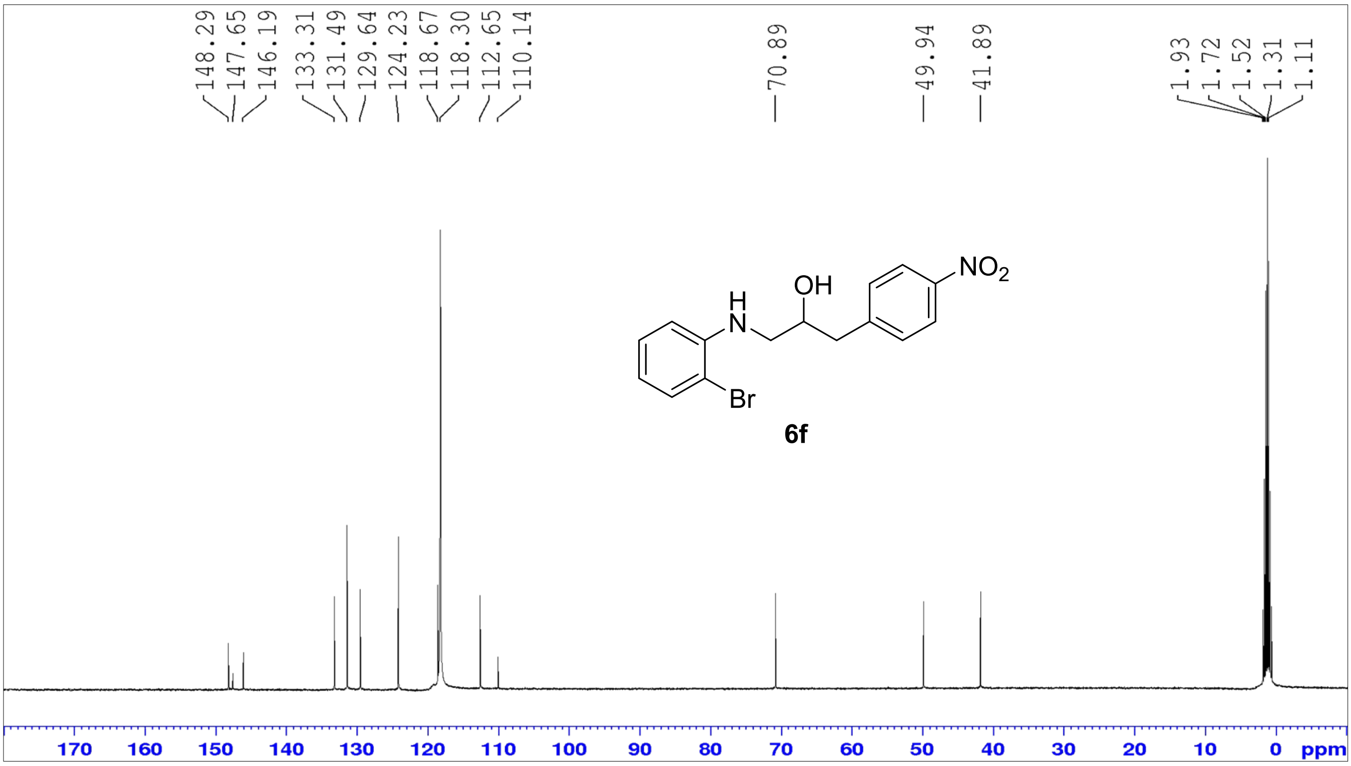

^{13}C NMR (100 MHz, CD_3CN) spectrum of compound **6f**.

^{1}H-NMR, and ^{13}C-NMR of 1-((2-iodophenyl)amino)-3-(4-nitrophenyl)propan-2-ol (6g)

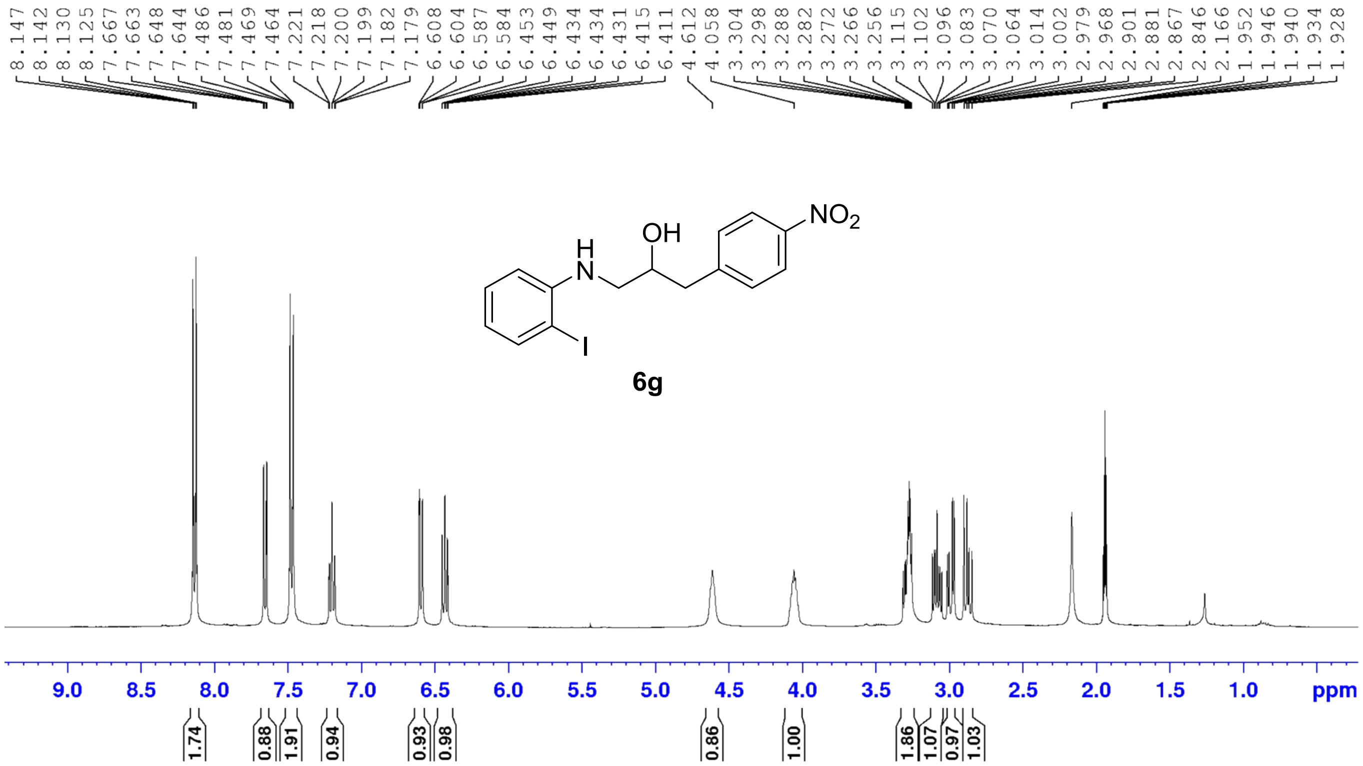

^{1}H NMR (400 MHz, CD_3CN) spectrum of compound **6g**.

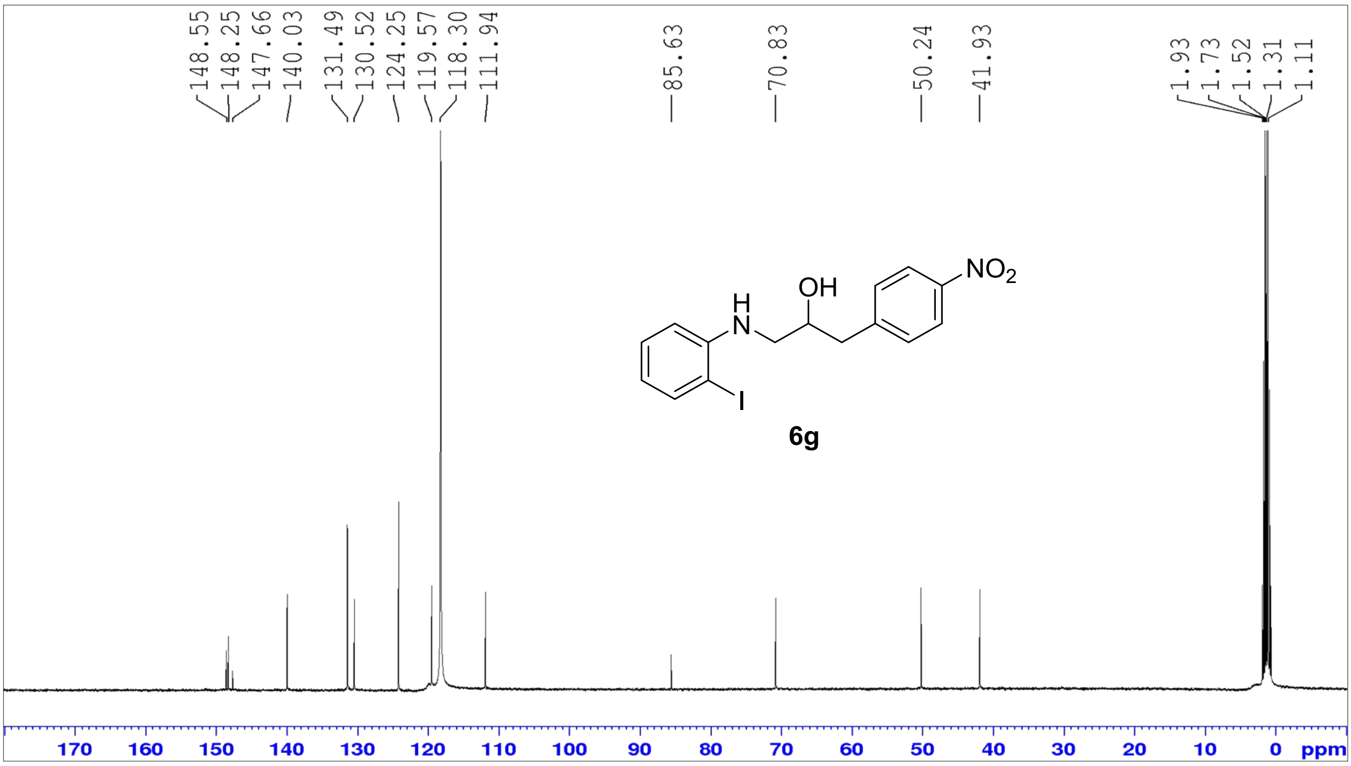

^{13}C NMR (100 MHz, CD_3CN) spectrum of compound **6g**.

^{1}H NMR, ^{13}C NMR, and ^{19}F NMR of 1-(4-nitrophenyl)-3-((4-(trifluoromethoxy)phenyl)amino)propan-2-ol (6h)

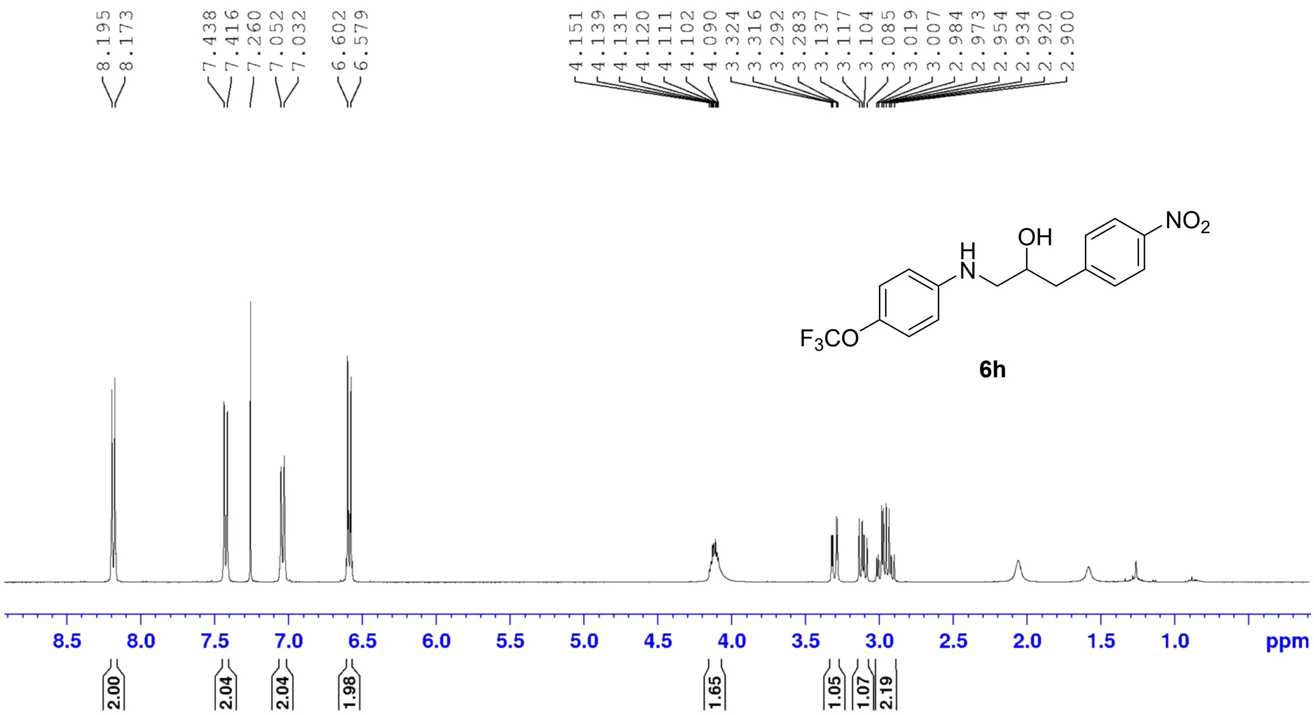

^{1}H NMR (400 MHz, $CDCl_3$) spectrum of compound **6h**.

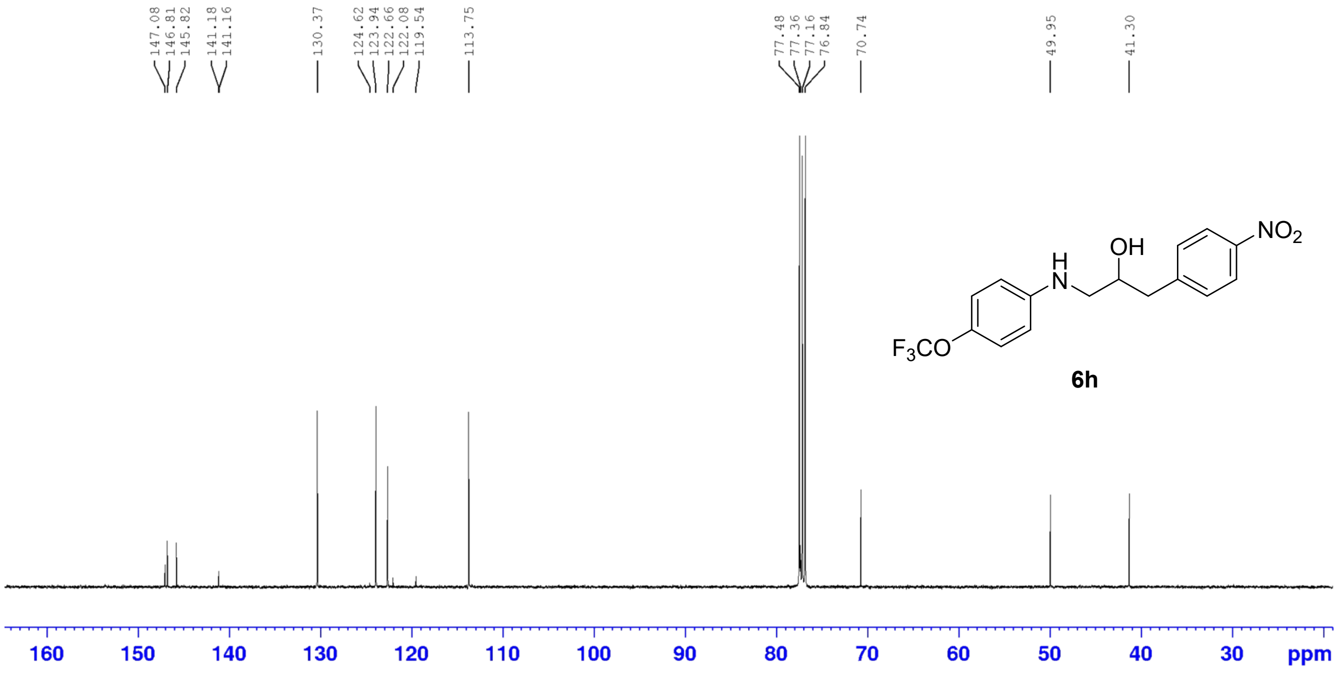

^{13}C NMR (100 MHz, $CDCl_3$) spectrum of compound **6h**.

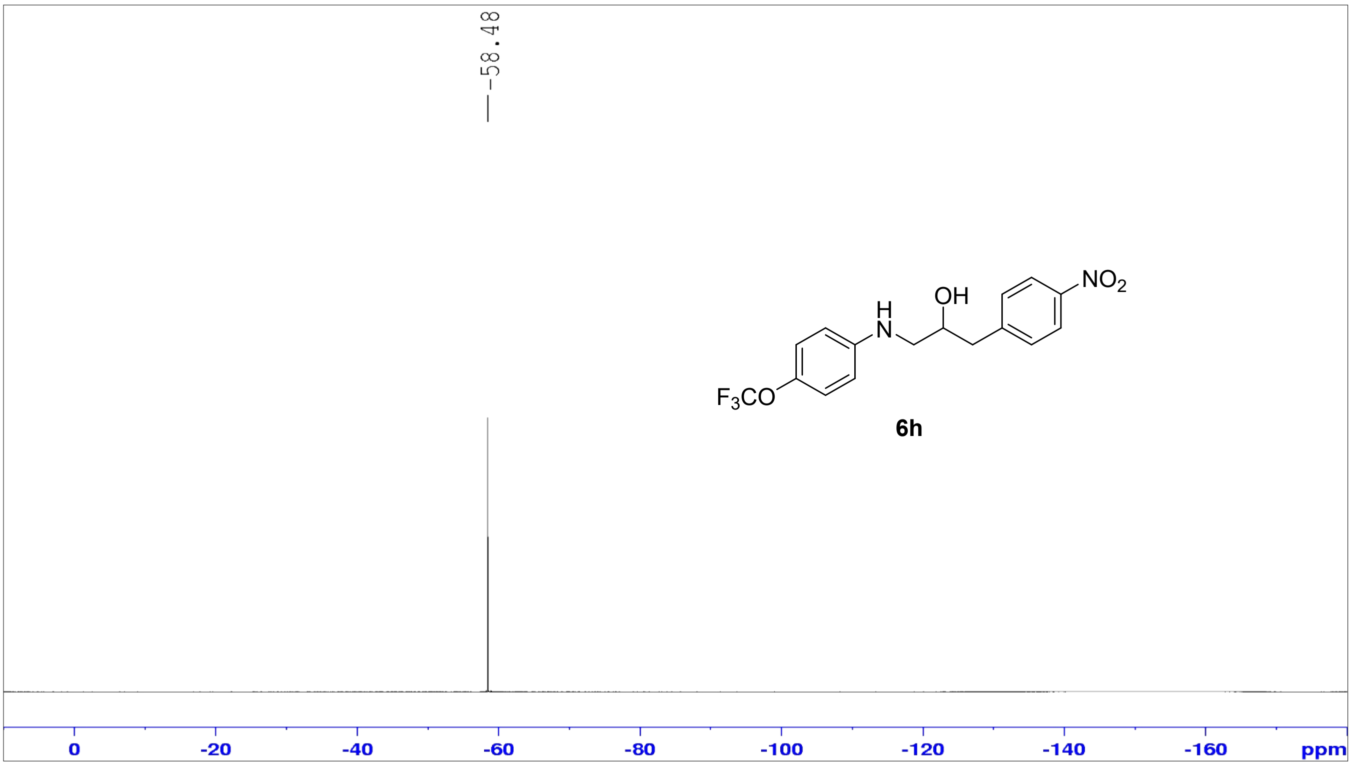

^{19}F NMR (376 MHz, $CDCl_3$) spectrum of compound **6h**.

^{1}H NMR, ^{19}F NMR, and ^{13}C NMR of 1-((4-difluoromethoxyphenyl)amino)-3-(4-nitrophenyl)propan-2-ol

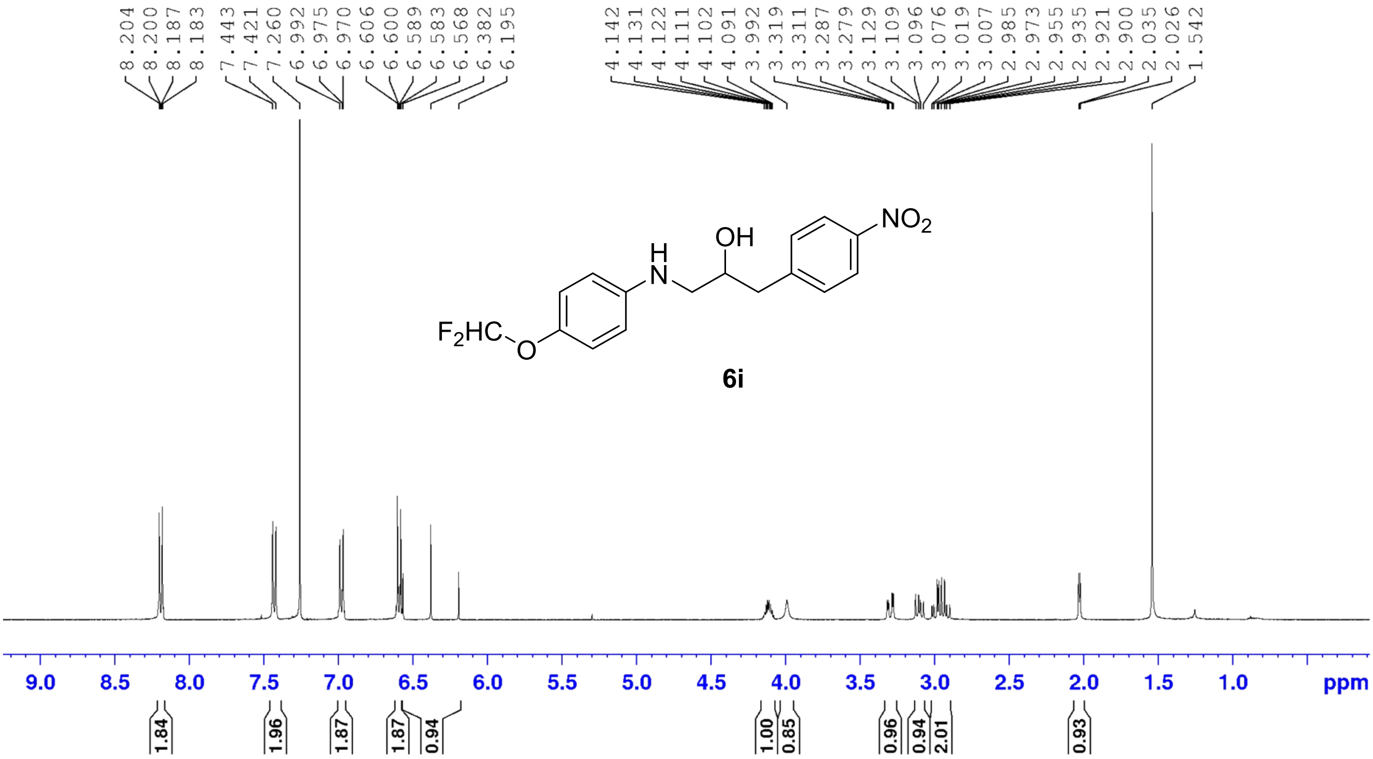

(6i)

^{1}H NMR (400 MHz, $CDCl_3$) spectrum of compound **6i**.

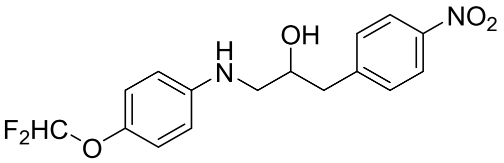

6i

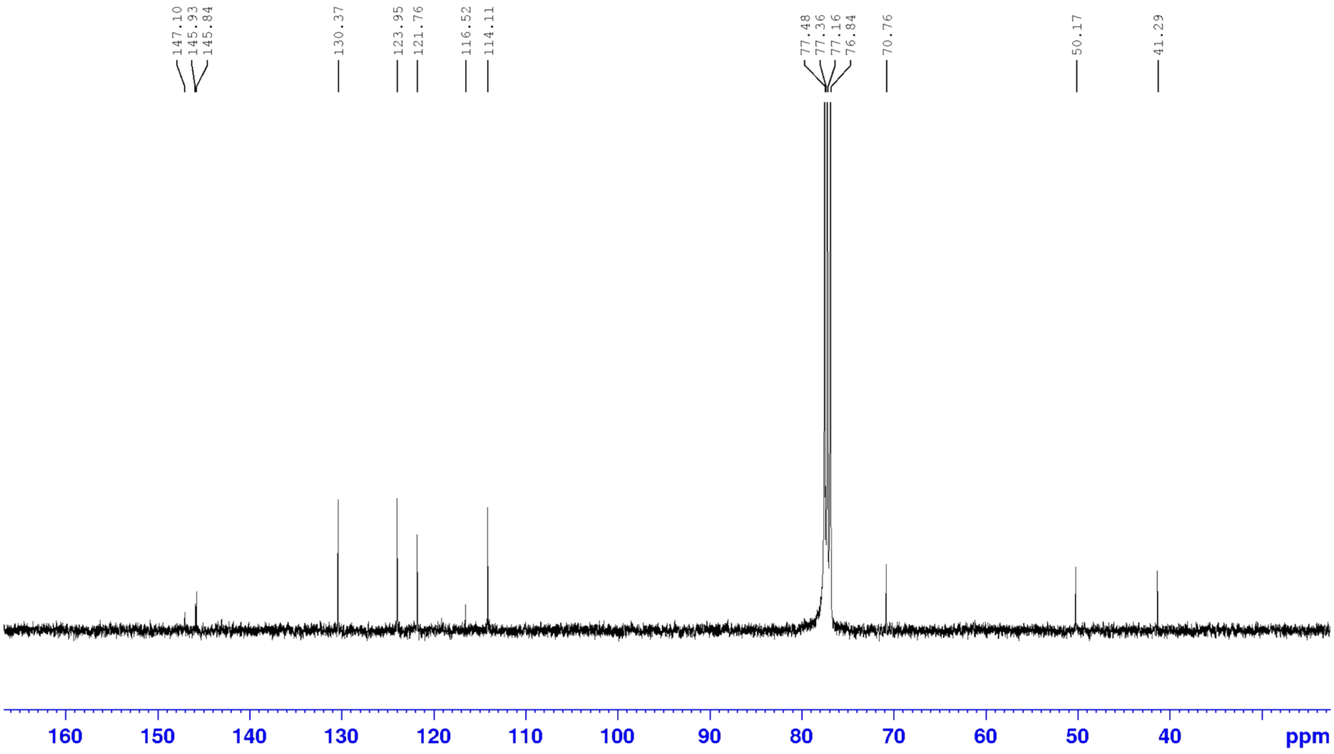

^{13}C NMR (100 MHz, $CDCl_3$) spectrum of compound **6i**.

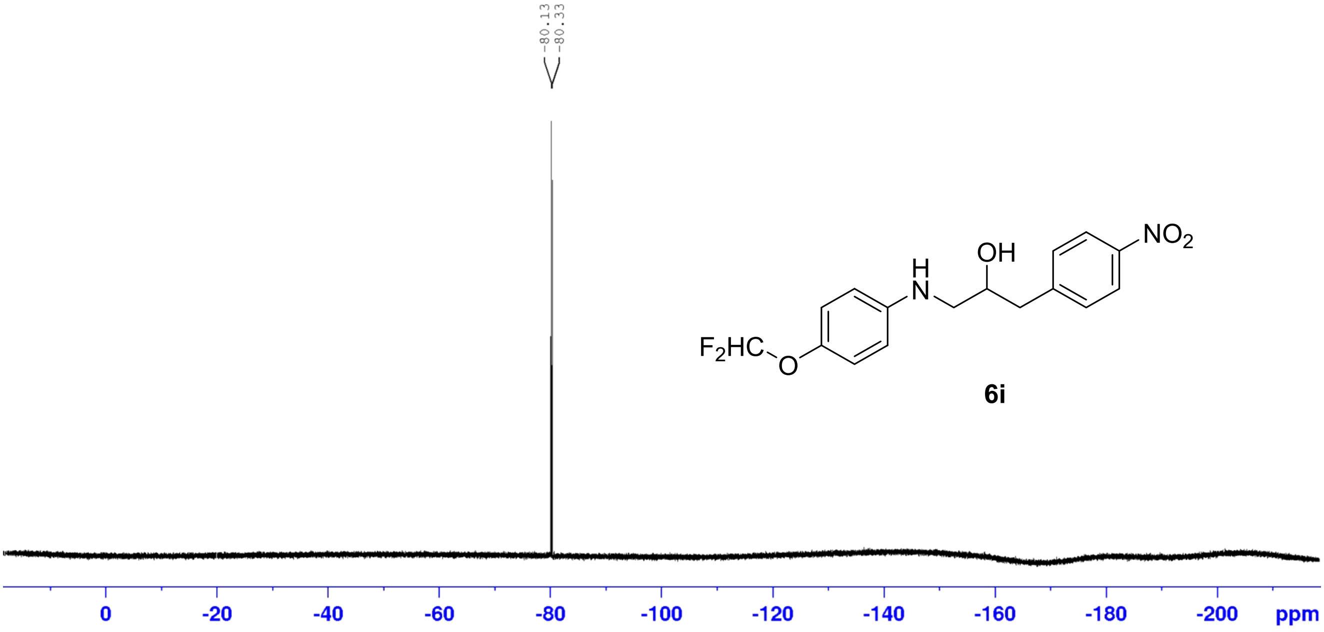

^{19}F NMR (376 MHz, $CDCl_3$) spectrum of compound **6i**.

^{1}H NMR, ^{19}F NMR, and ^{13}C NMR of 1-((2-bromo-4-trifluoromethoxyphenyl)amino)-3-(4-nitorphenyl)propan-2-ol (6j)

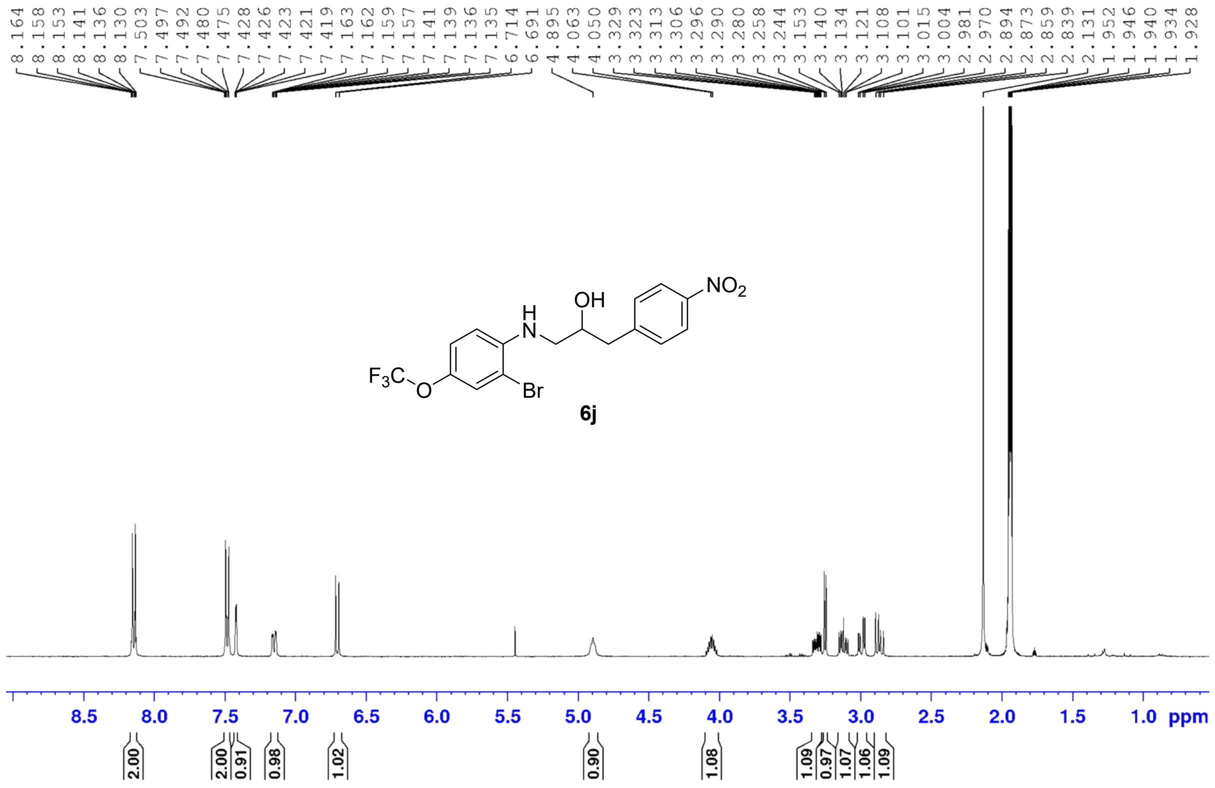

^{1}H NMR (400 MHz, CD_3CN) spectrum of compound **6j**.

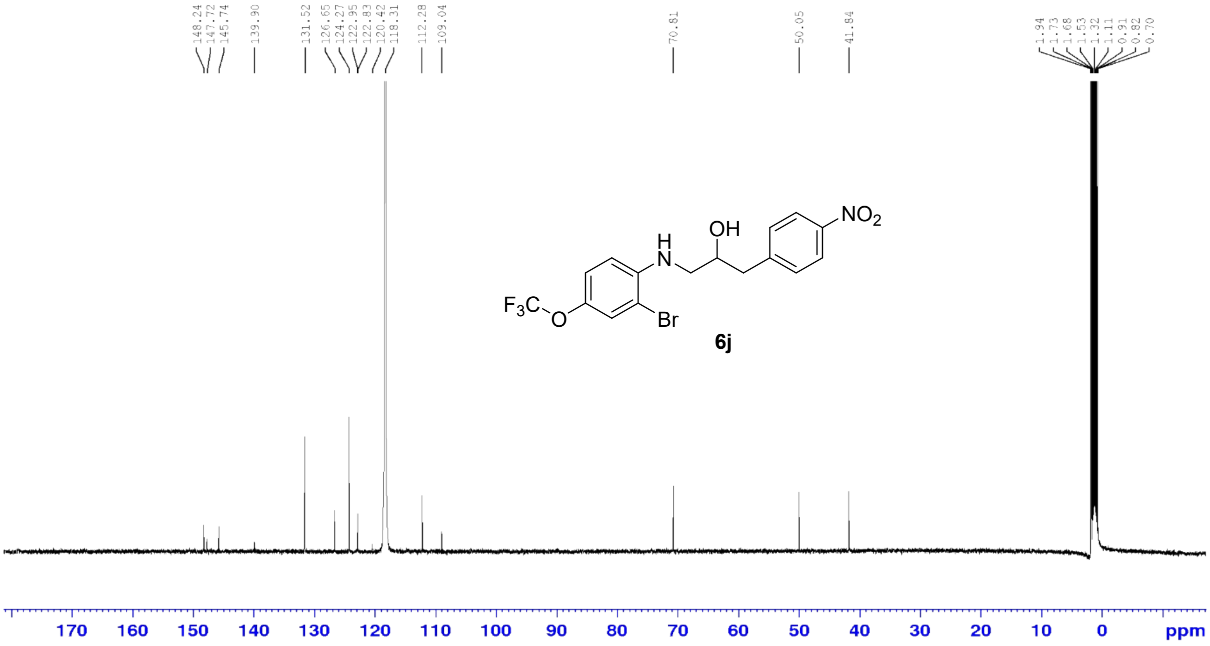

^{13}C NMR (100 MHz, CD_3CN) spectrum of compound **6j**.

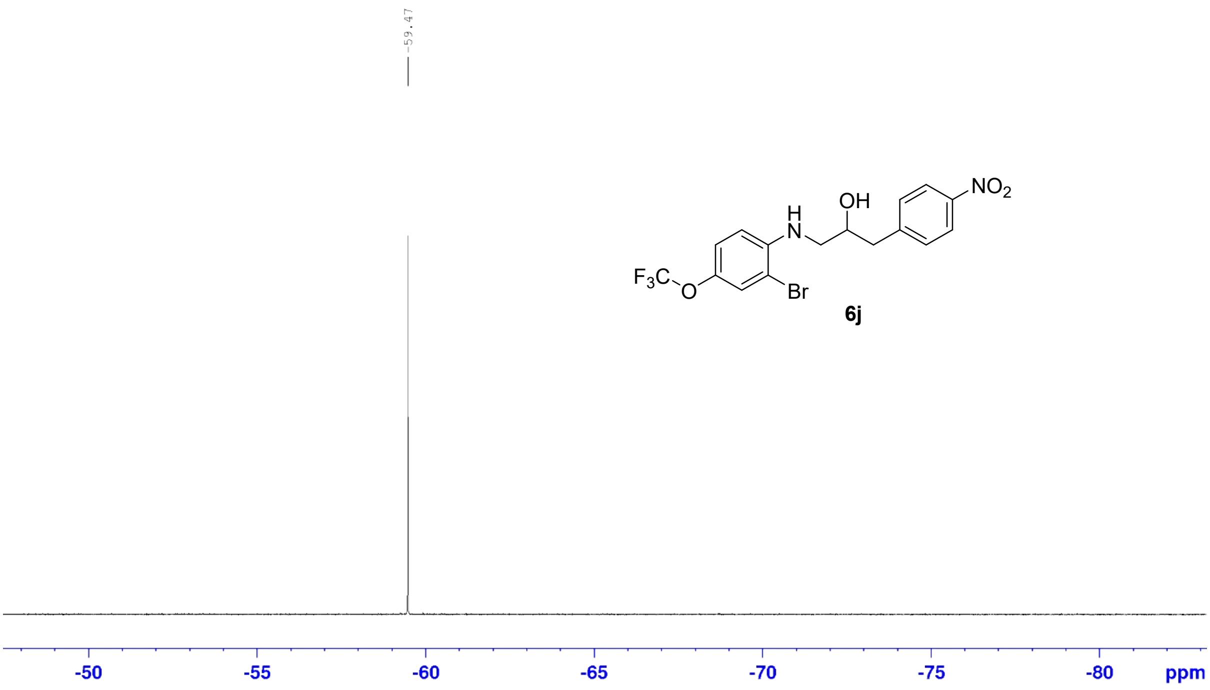

^{19}F NMR (376 MHz, CD_3CN) spectrum of compound **6j**.

^{1}H NMR, ^{19}F NMR, and ^{13}C NMR of 1-((4-methyl-3-trifluoromethylphenyl)amino)-3-(4-nitorphenyl)propan-2-ol (6k)

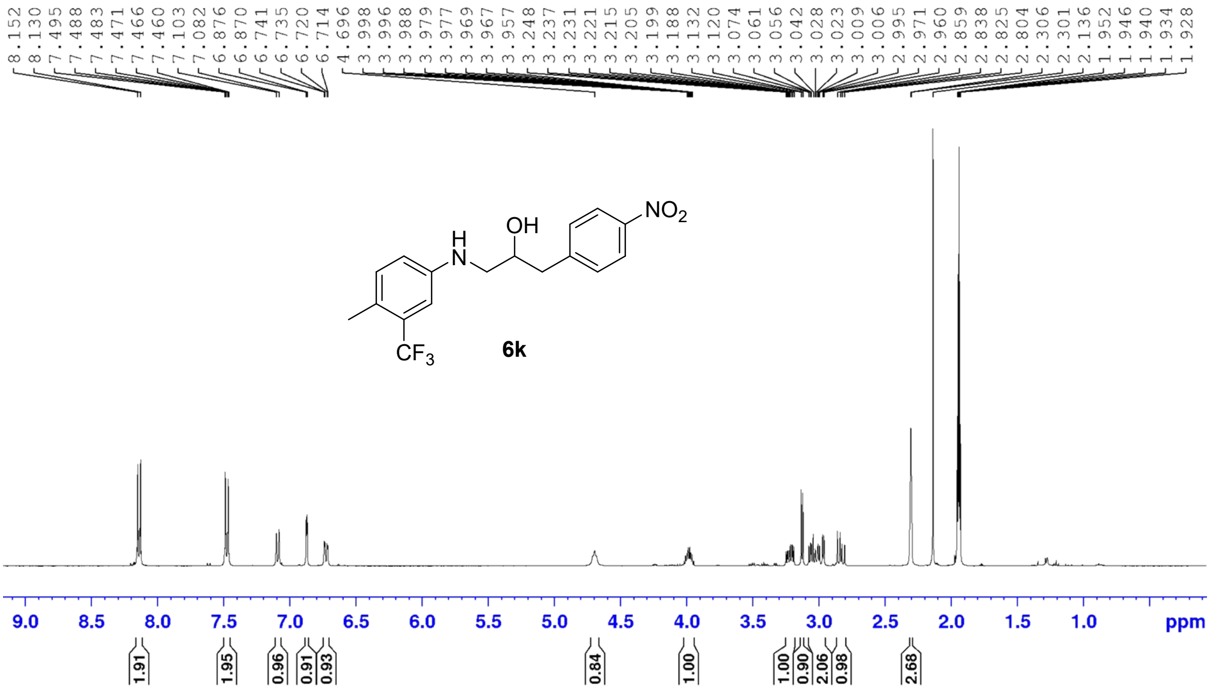

^{1}H NMR (400 MHz, CD_3CN) spectrum of compound **6k**.

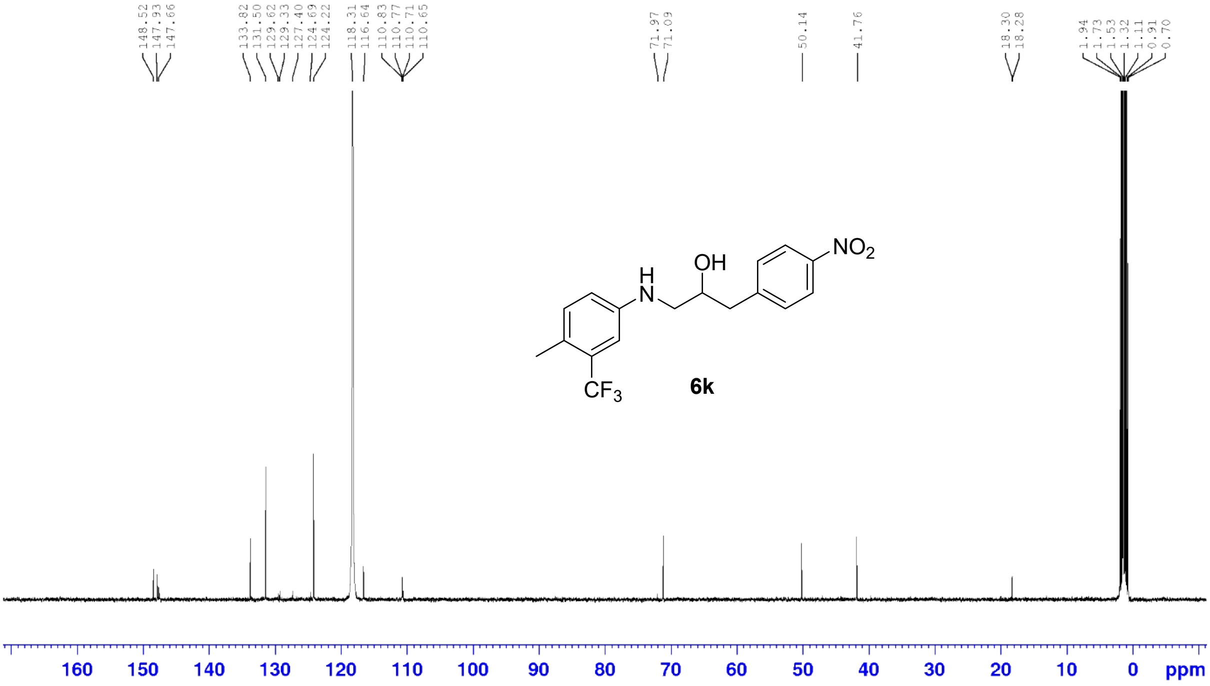

^{13}C NMR (100 MHz, CD_3CN) spectrum of compound **6k**.

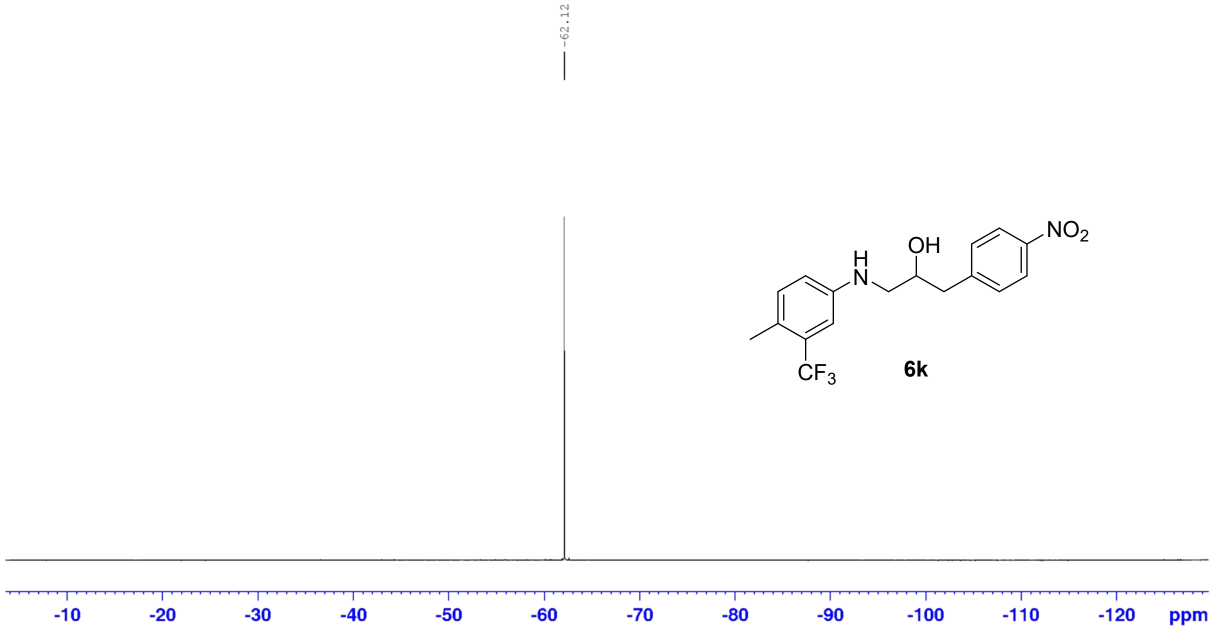

^{19}F NMR (376 MHz, CD_3CN) spectrum of compound **6k**.

^{1}H NMR, ^{19}F NMR, and ^{13}C NMR of 1-((6-bromo-2,3,4-trifluorophenyl)amino)-3-(4-nitrophenyl)propan-2-ol (6l)

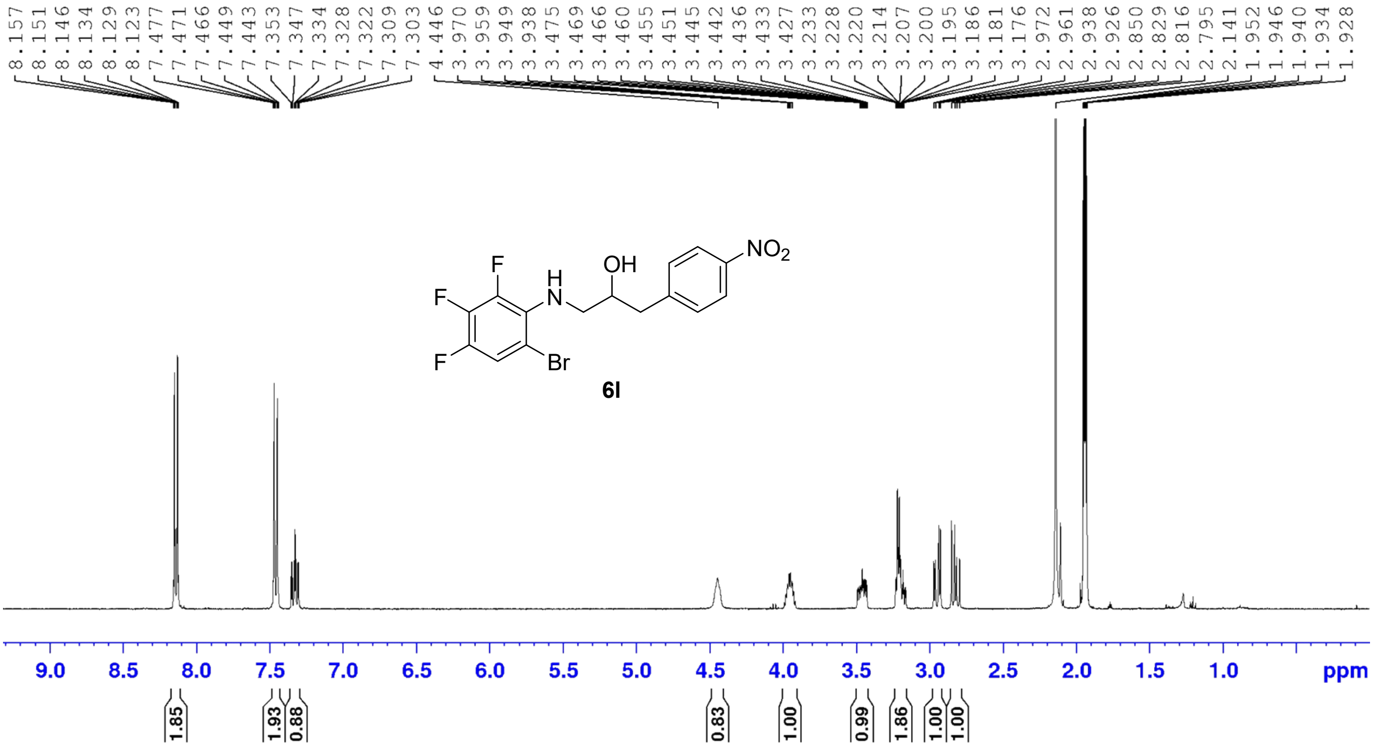

^{1}H NMR (400 MHz, CD_3CN) spectrum of compound **6l**.

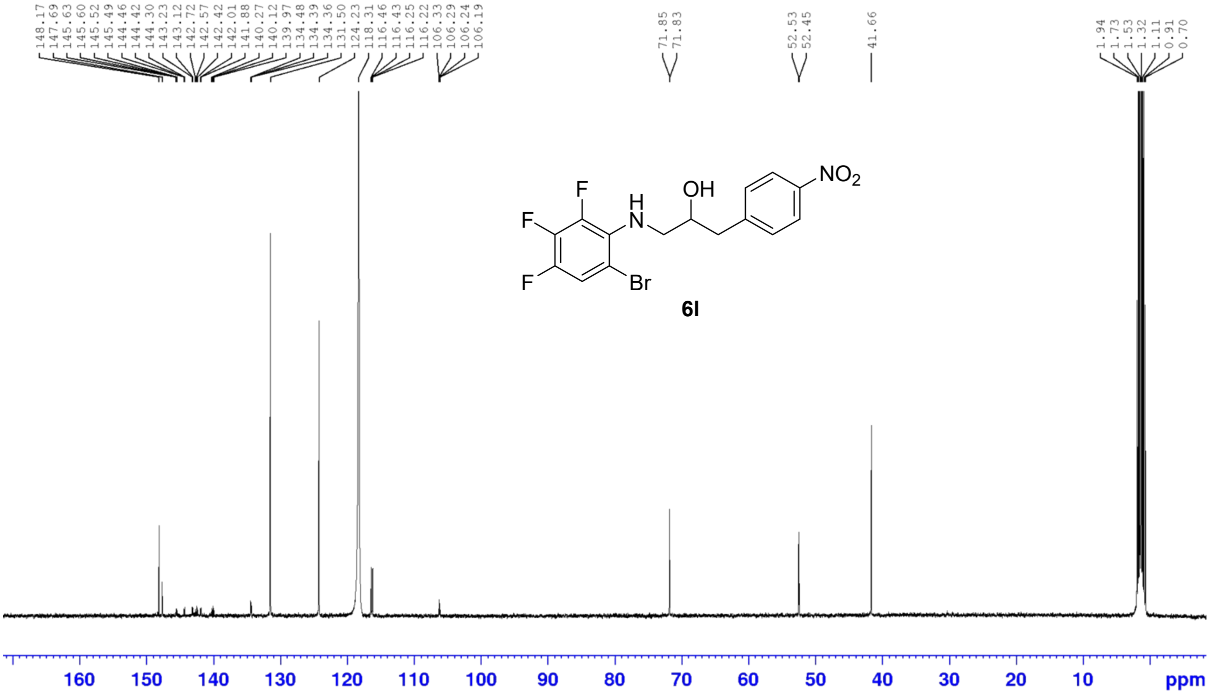

^{13}C NMR (100 MHz, CD_3CN) spectrum of compound **6l**.

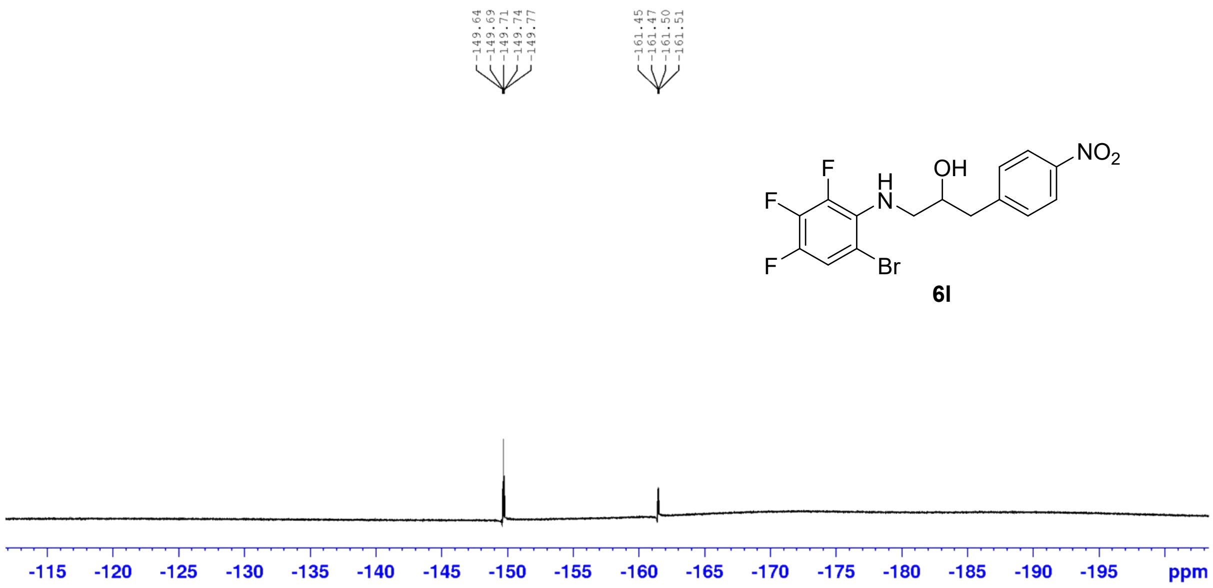

^{19}F NMR (376 MHz, CD_3CN) spectrum of compound **6l**.

^{1}H NMR and ^{13}C NMR of 1-((4-nitrophenyl)amino)-3-(4-nitrophenyl)propan-2-ol (6m)

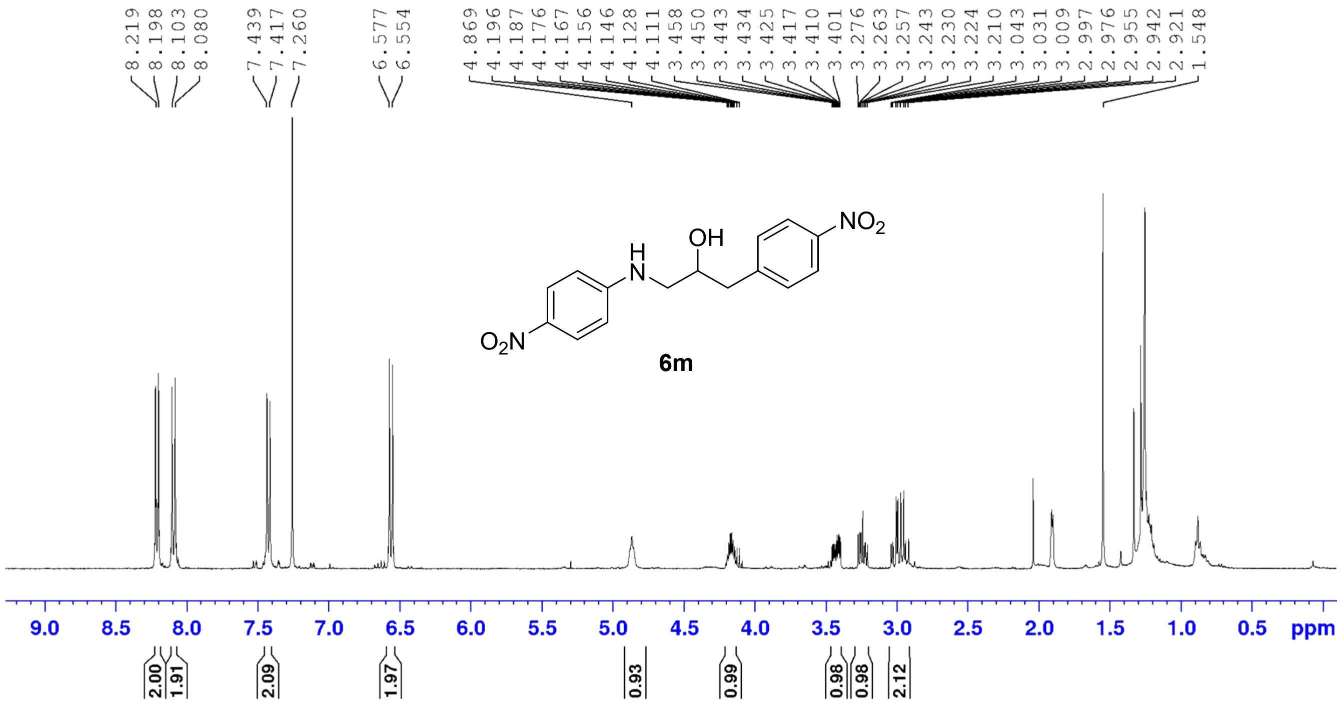

^{1}H NMR (400 MHz, $CDCl_3$) spectrum of compound **6m**.

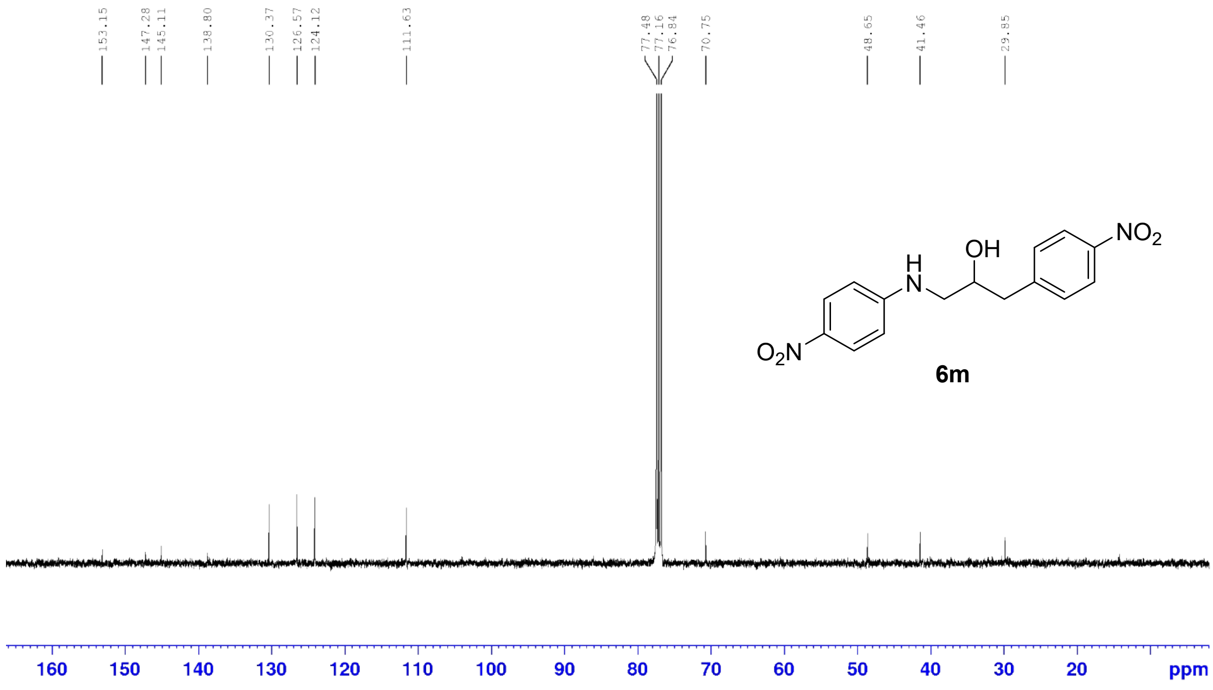

^{13}C NMR (100 MHz, $CDCl_3$) spectrum of compound **6m**.

^{1}H NMR, ^{13}C NMR, and ^{19}F NMR of 1-((2-fluoro-4-nitrophenyl)amino)-3-(4-nitrophenyl)propan-2-ol (6n)

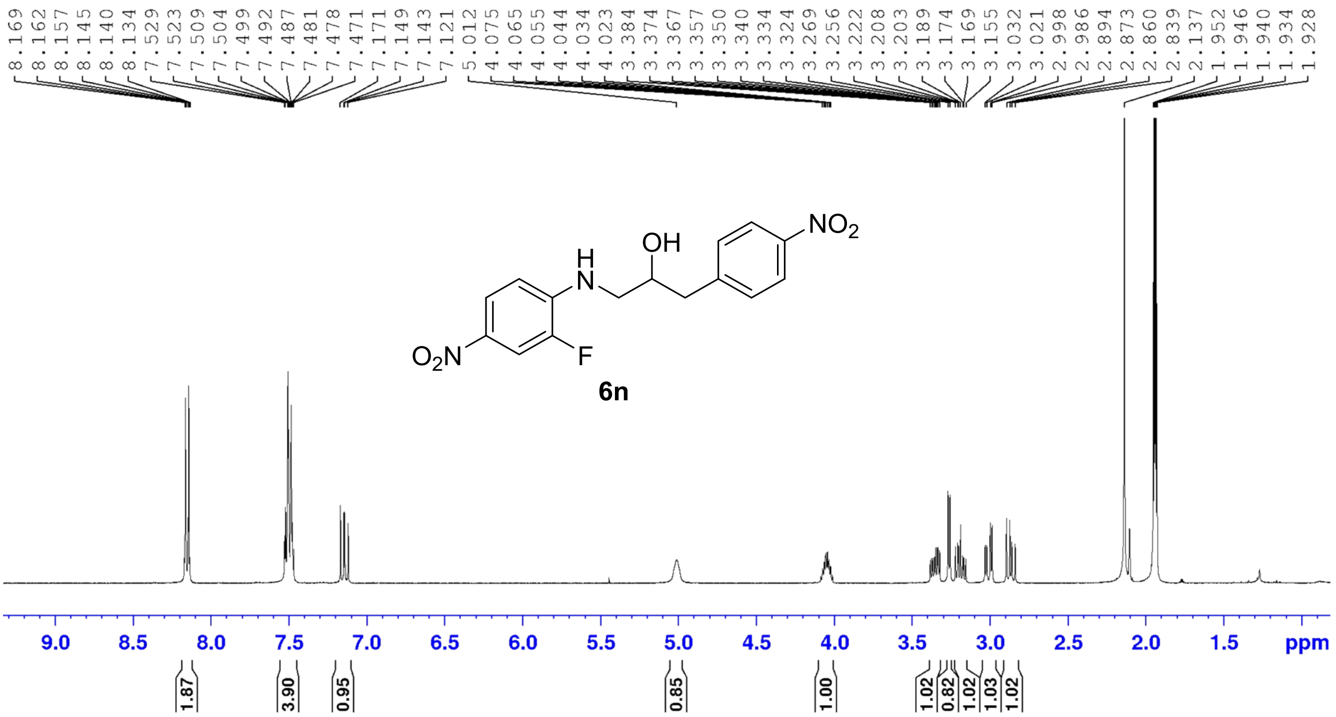

^{1}H NMR (400 MHz, CD_3CN) spectrum of compound **6n**.

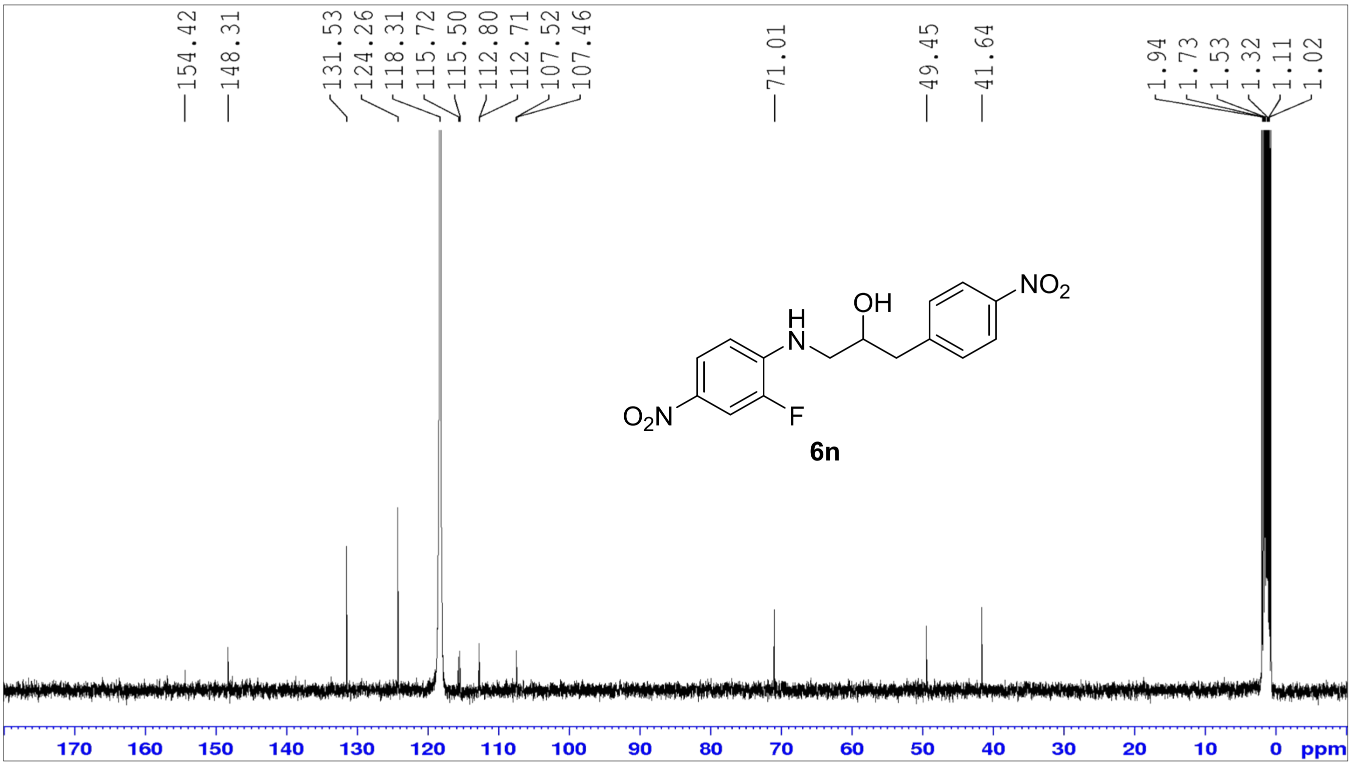

^{13}C NMR (100 MHz, CD_3CN) spectrum of compound **6n**.

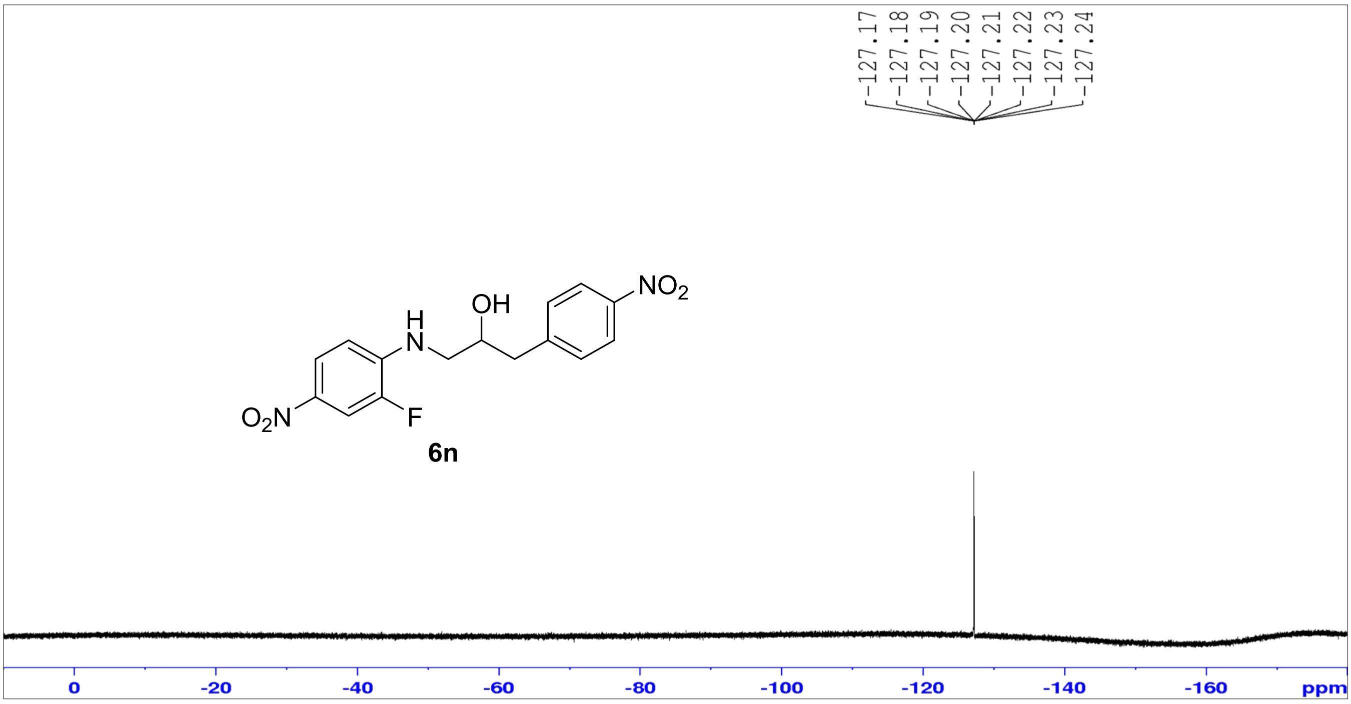

^{19}F NMR (376 MHz, CD_3CN) spectrum of compound **6n**.

^{1}H-NMR, ^{13}C-NMR, and ^{19}F-NMR of 1-((4-fluoro-2-nitrophenyl)amino)-3-(4-nitrophenyl)propan-2-ol (6o).

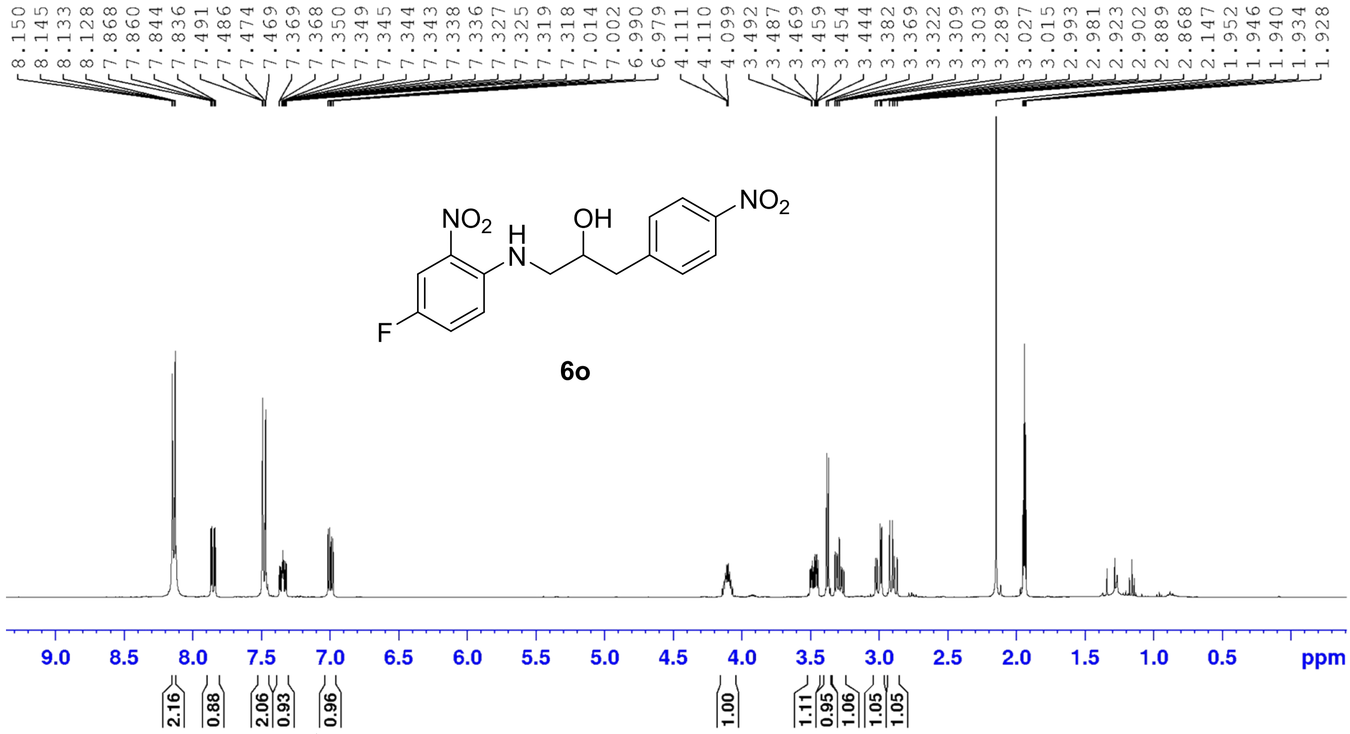

^{1}H NMR (400 MHz, CD_3CN) spectrum of compound **6o**.

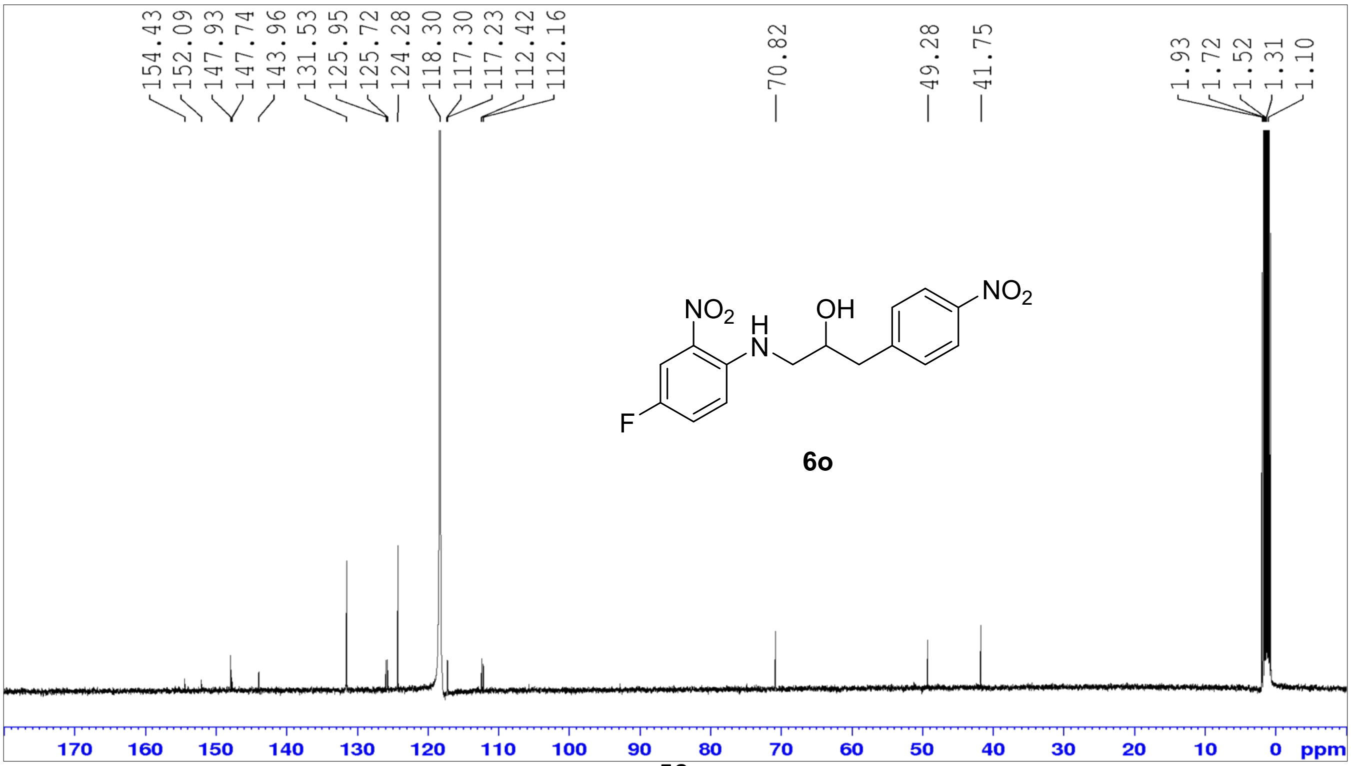

^{13}C NMR (100 MHz, CD_3CN) spectrum of compound **6o**.

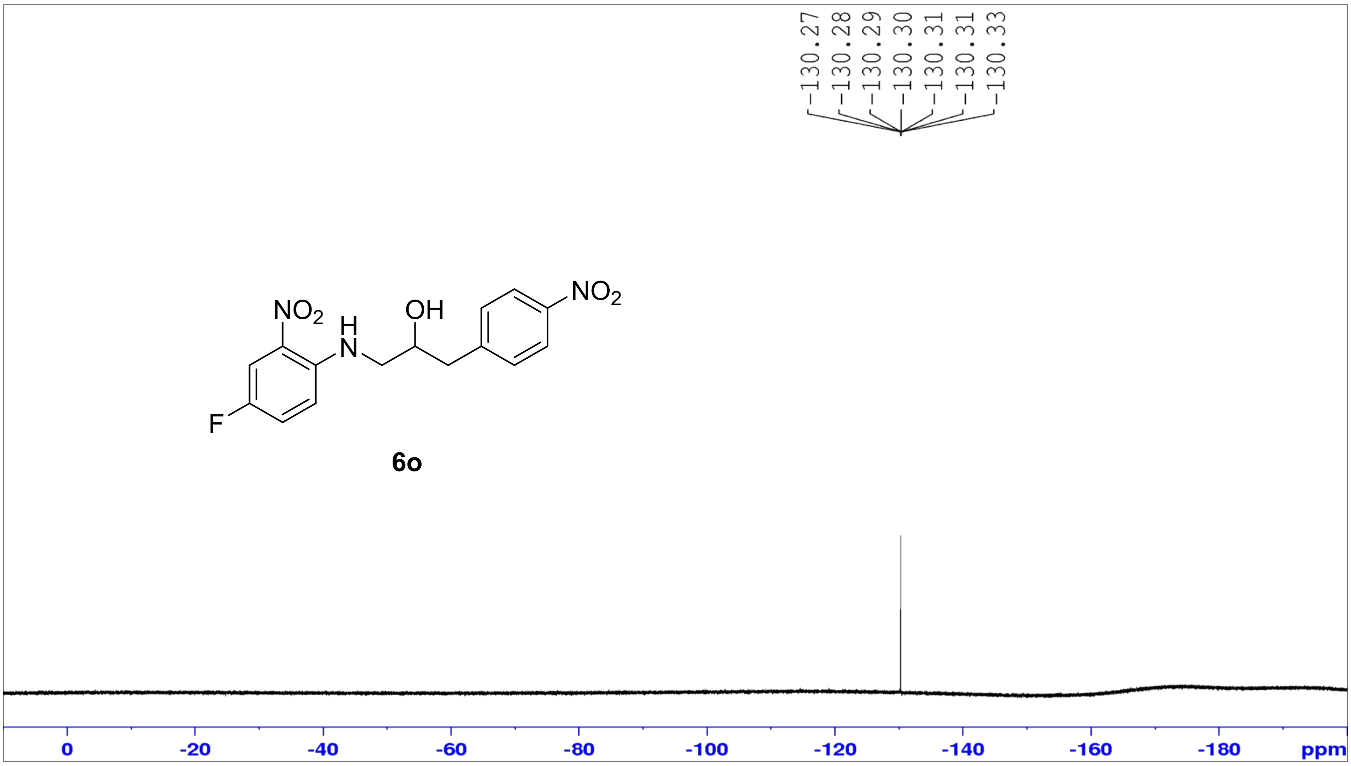

^{19}F NMR (376 MHz, CD_3CN) spectrum of compound **6o**.

^{1}H NMR, ^{19}F NMR, and ^{13}C NMR of 1-((5-Fluoro-2-nitrophenyl)amino)-3-(4-nitrophenyl)propan-2-ol (6p)

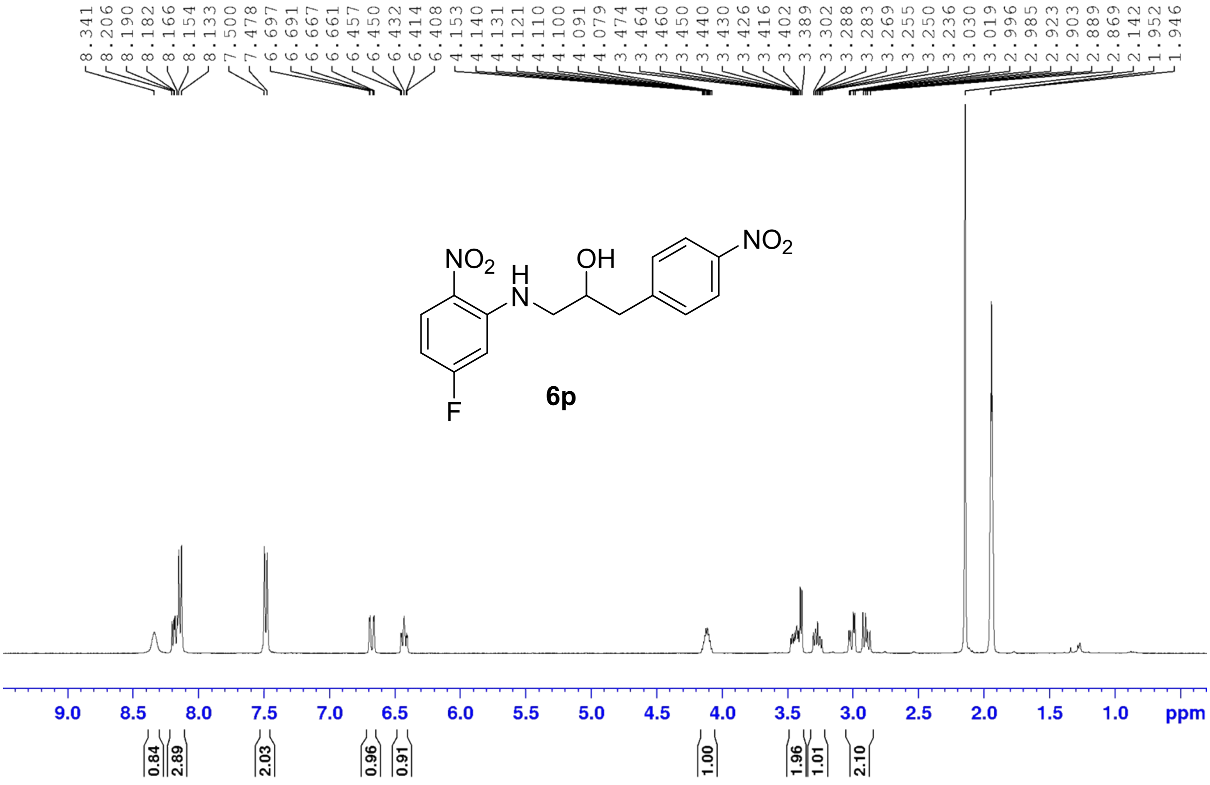

^{1}H NMR (400 MHz, CD_3CN) spectrum of compound **6p**.

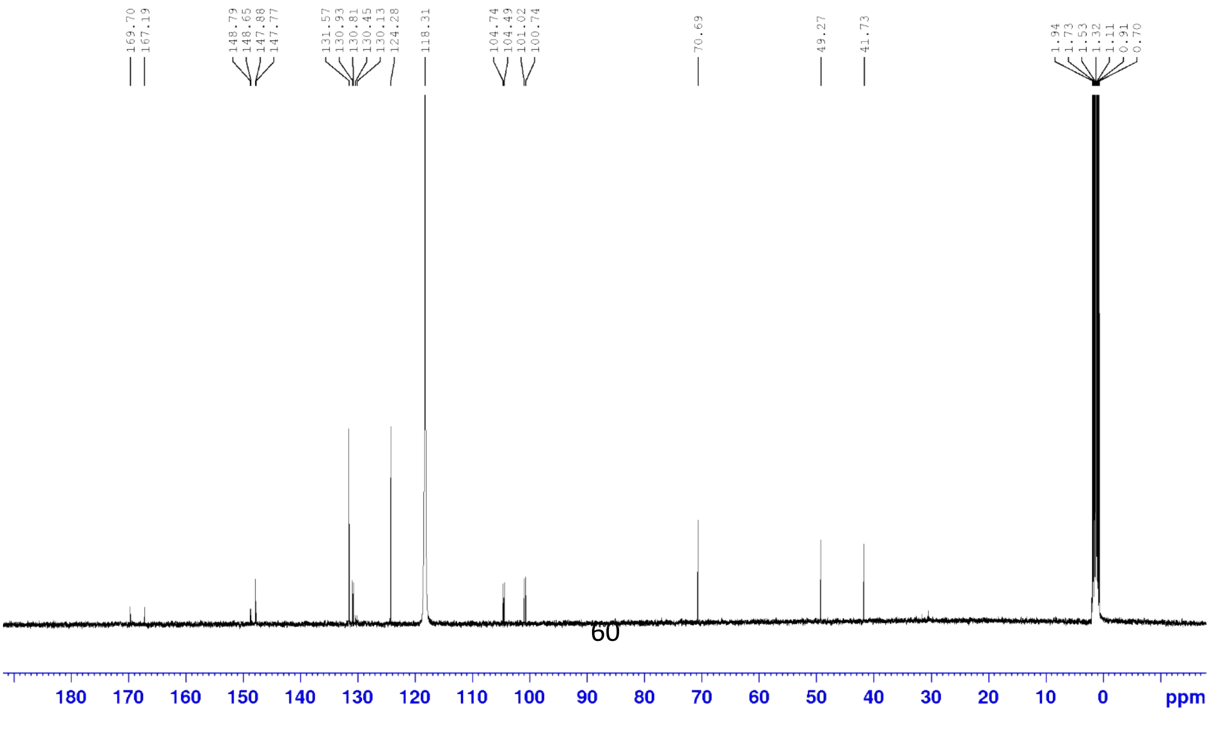

^{13}C NMR (100 MHz, CD_3CN) spectrum of compound **6p**.

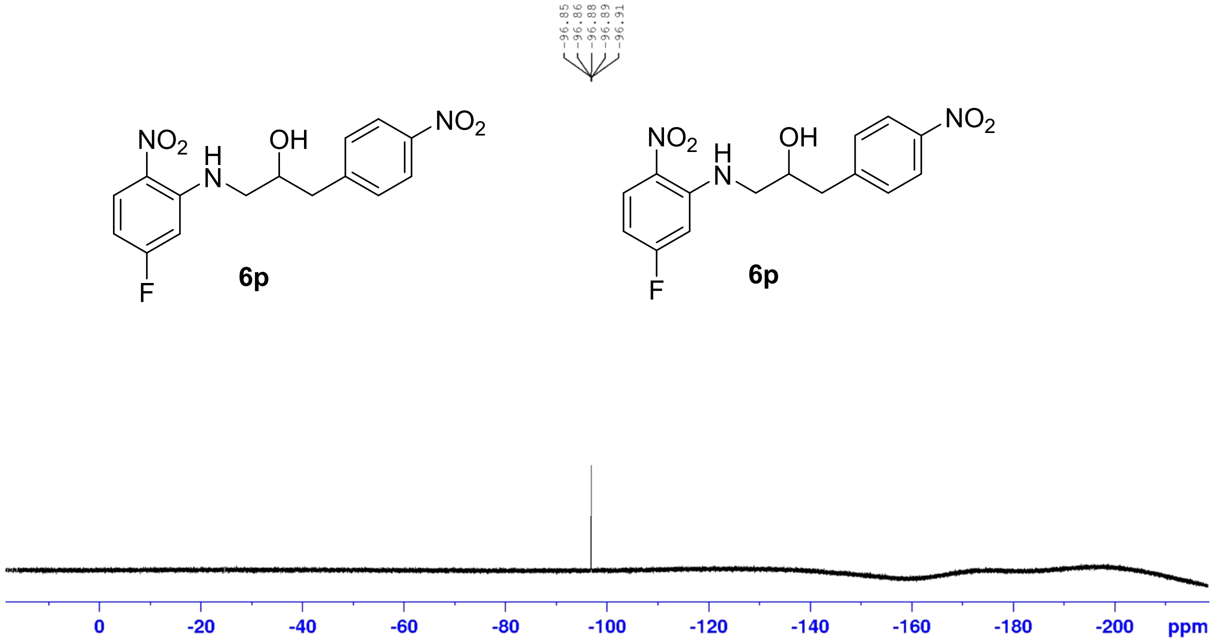

^{19}F NMR (376 MHz, CD_3CN) spectrum of compound **6p**.

^{1}H-NMR, ^{13}C-NMR, and ^{19}F-NMR of 1-((2-fluoro-6-nitrophenyl)amino)-3-(4-nitrophenyl)propan-2-ol (6q)

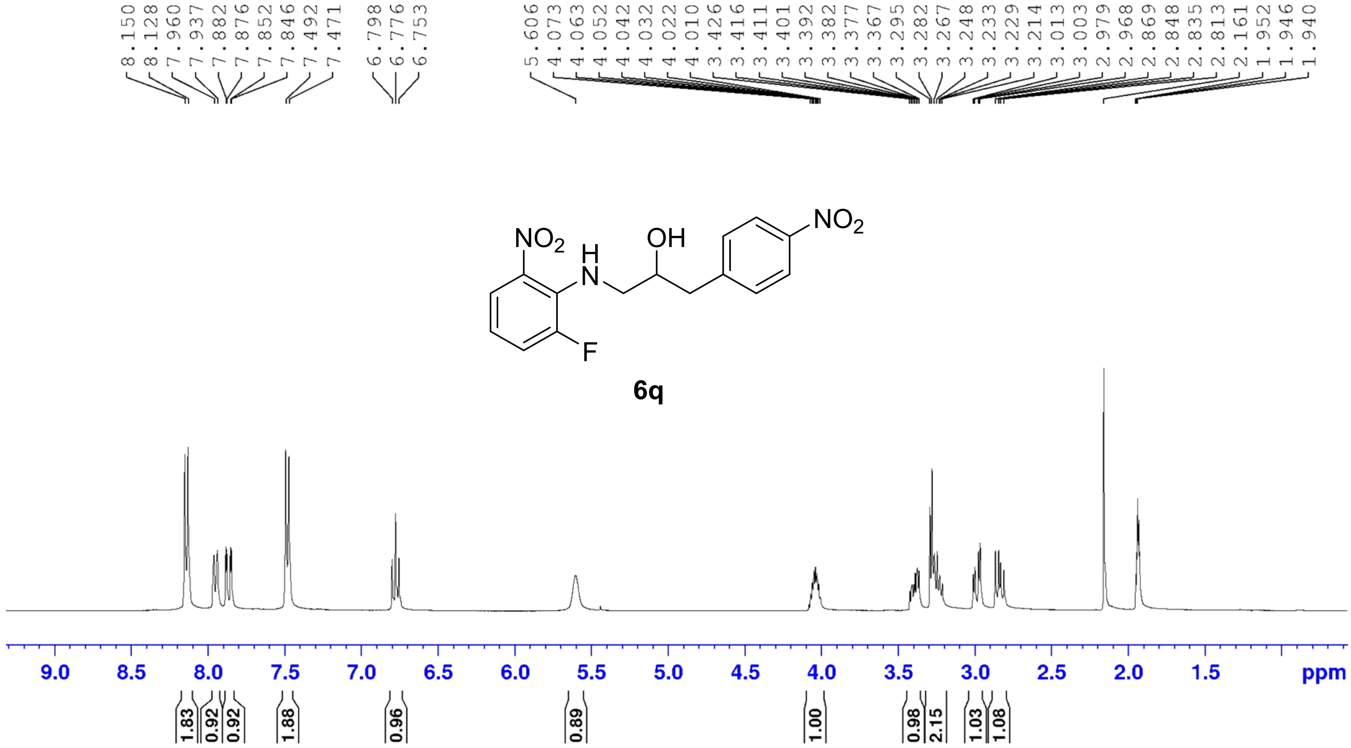

^{1}H NMR (400 MHz, CD_3CN) spectrum of compound **6q**.

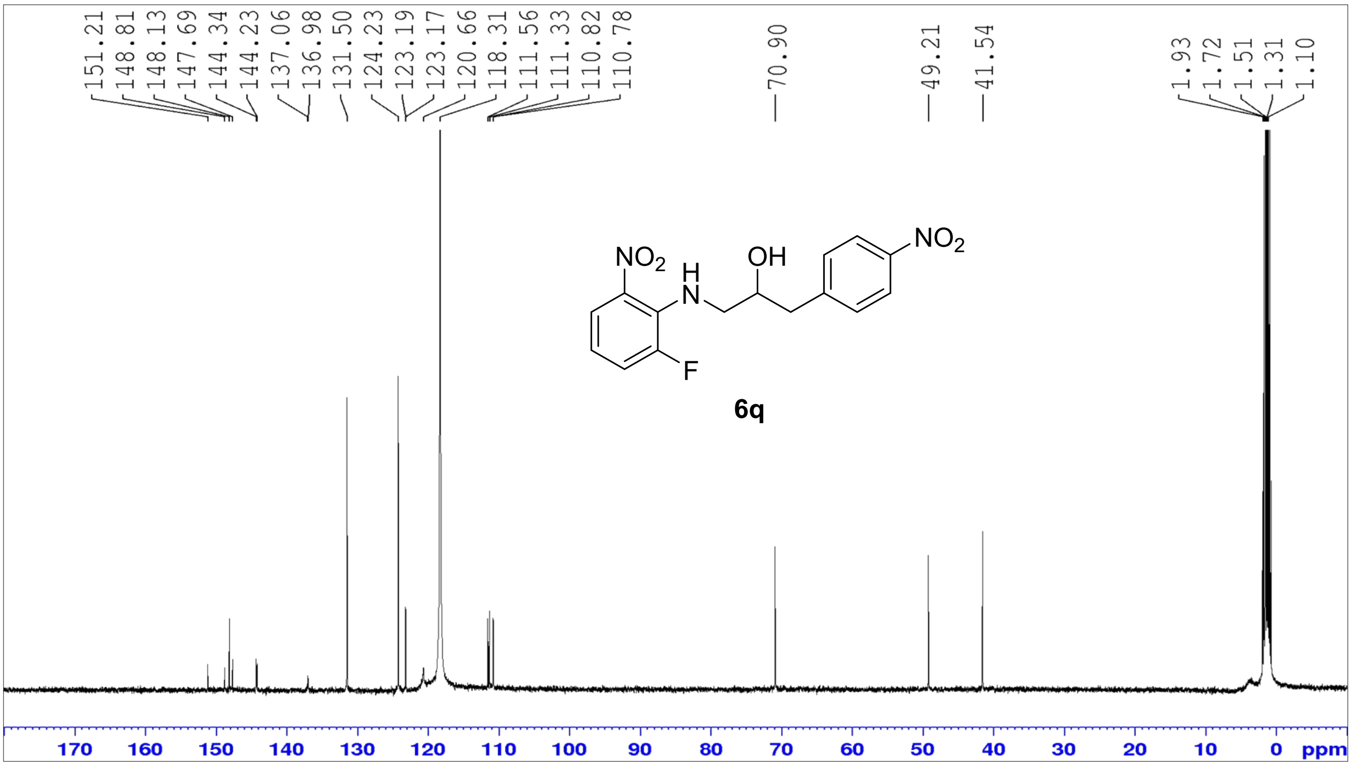

^{13}C NMR (100 MHz, CD_3CN) spectrum of compound **6q**.

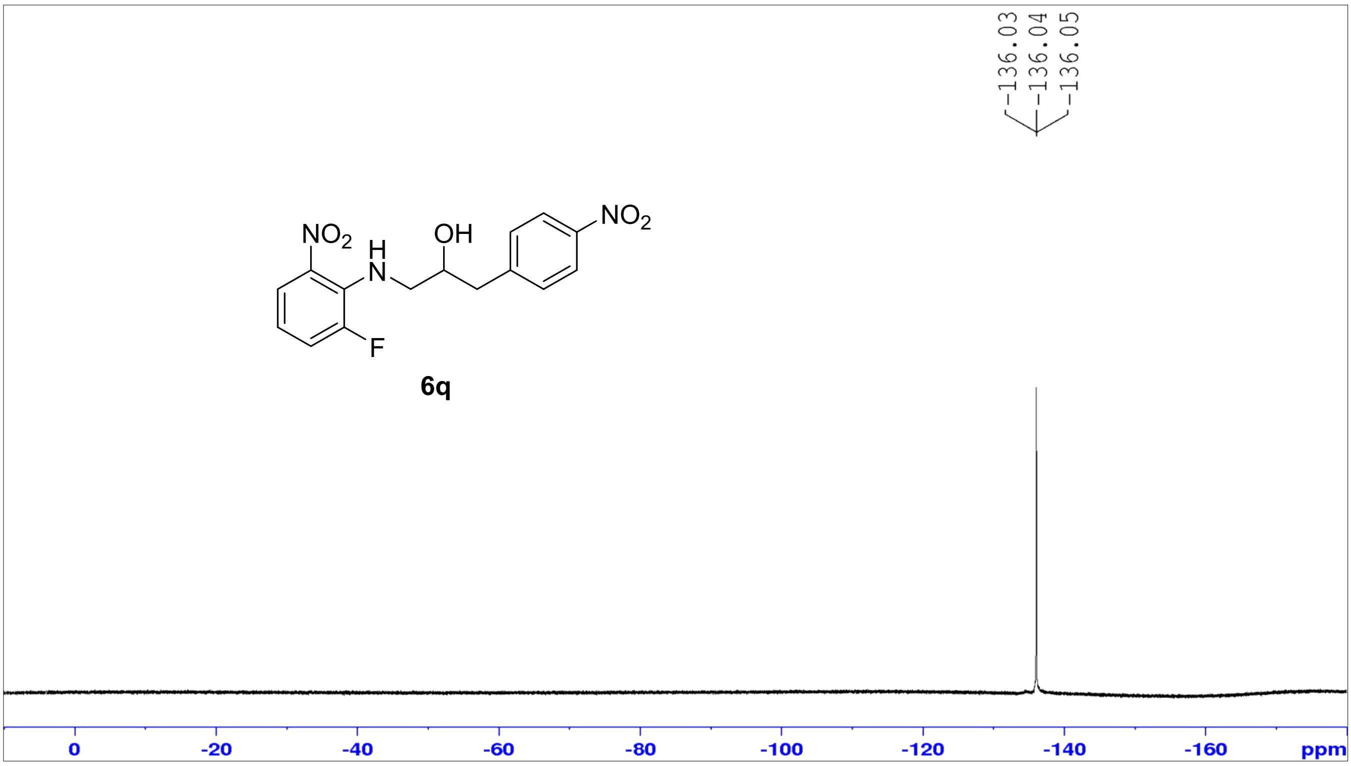

^{19}F NMR (376 MHz, CD_3CN) spectrum of compound **6q**.

^{1}H NMR, ^{13}C NMR, and ^{19}F NMR of 1-((2-nitro-4-(trifluoromethoxy)phenyl)amino)-3-(4-nitrophenyl)propan-2-ol (6r)

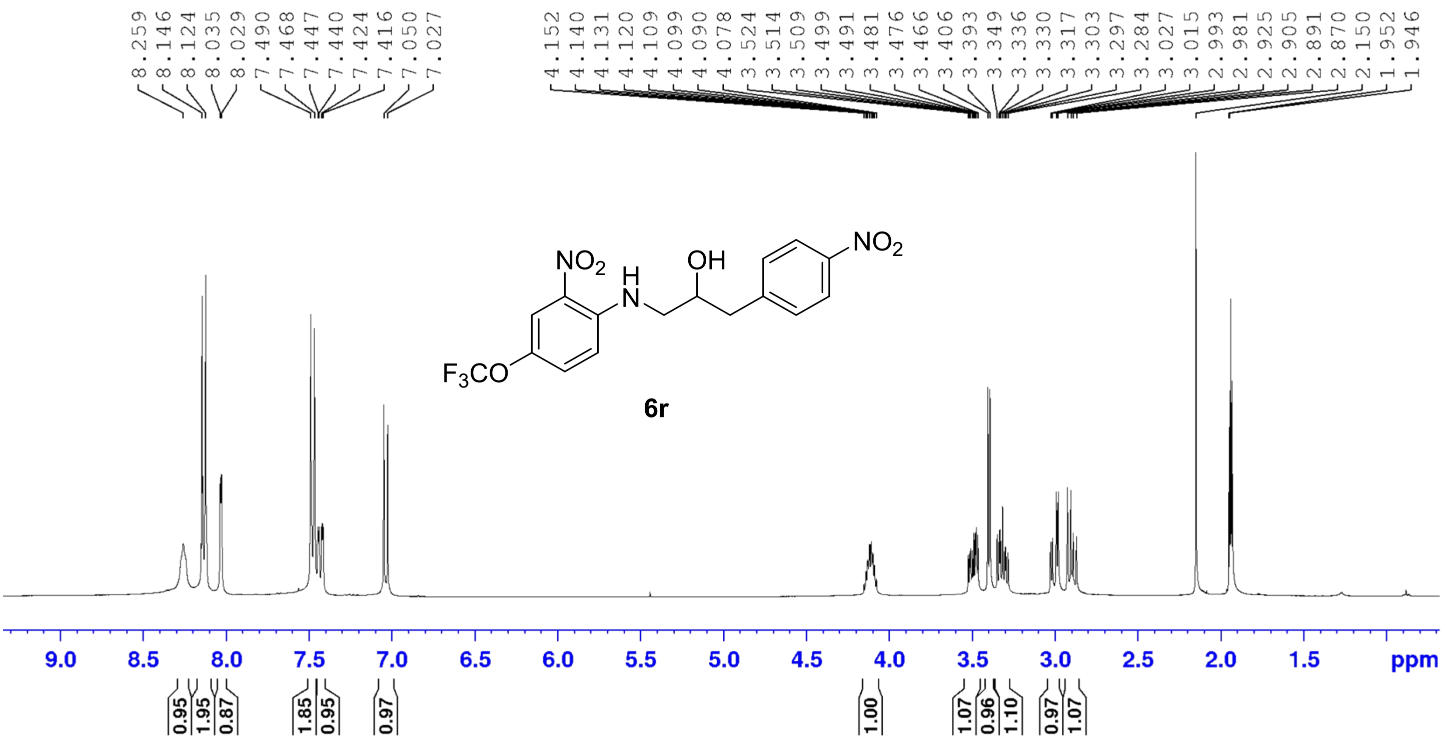

^{1}H NMR (400 MHz, CD_3CN) spectrum of compound (**6r**).

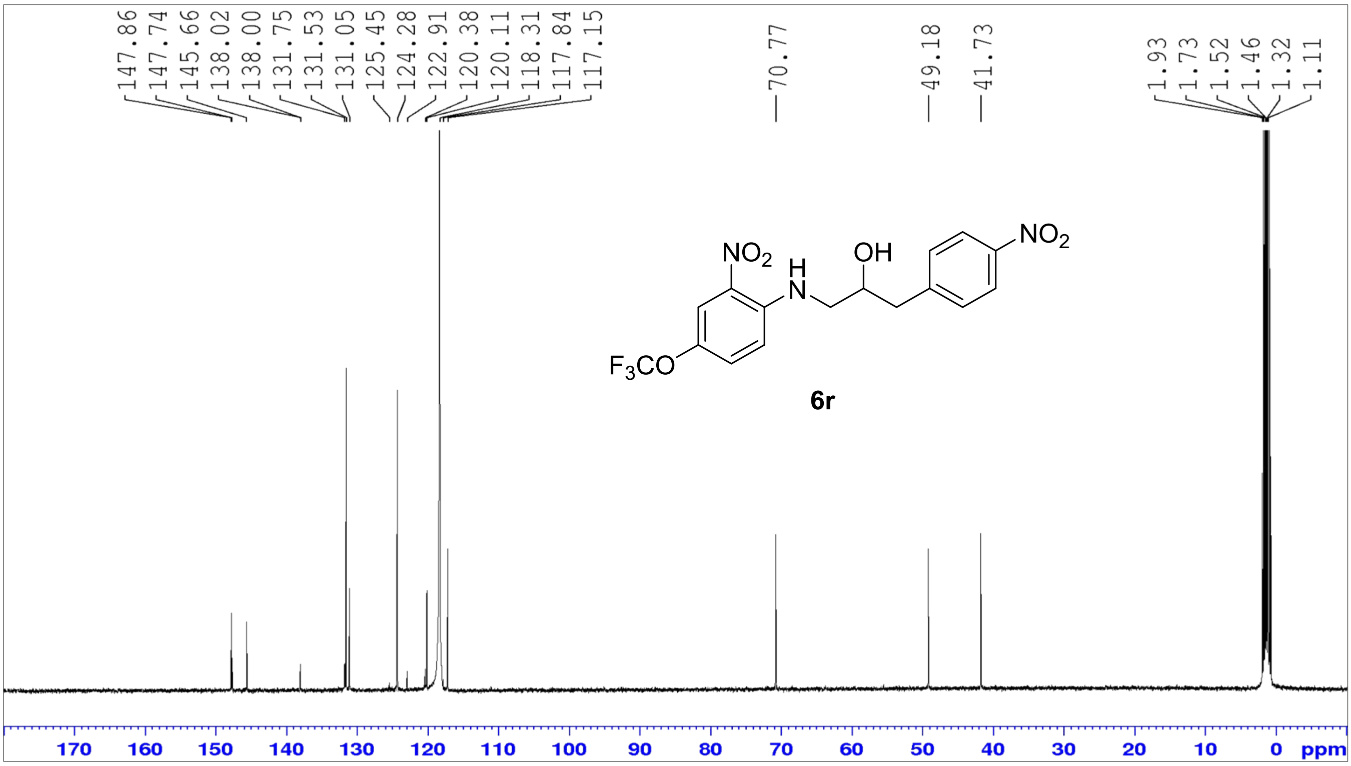

^{13}C NMR (100 MHz, CD_3CN) spectrum of compound **6r**.

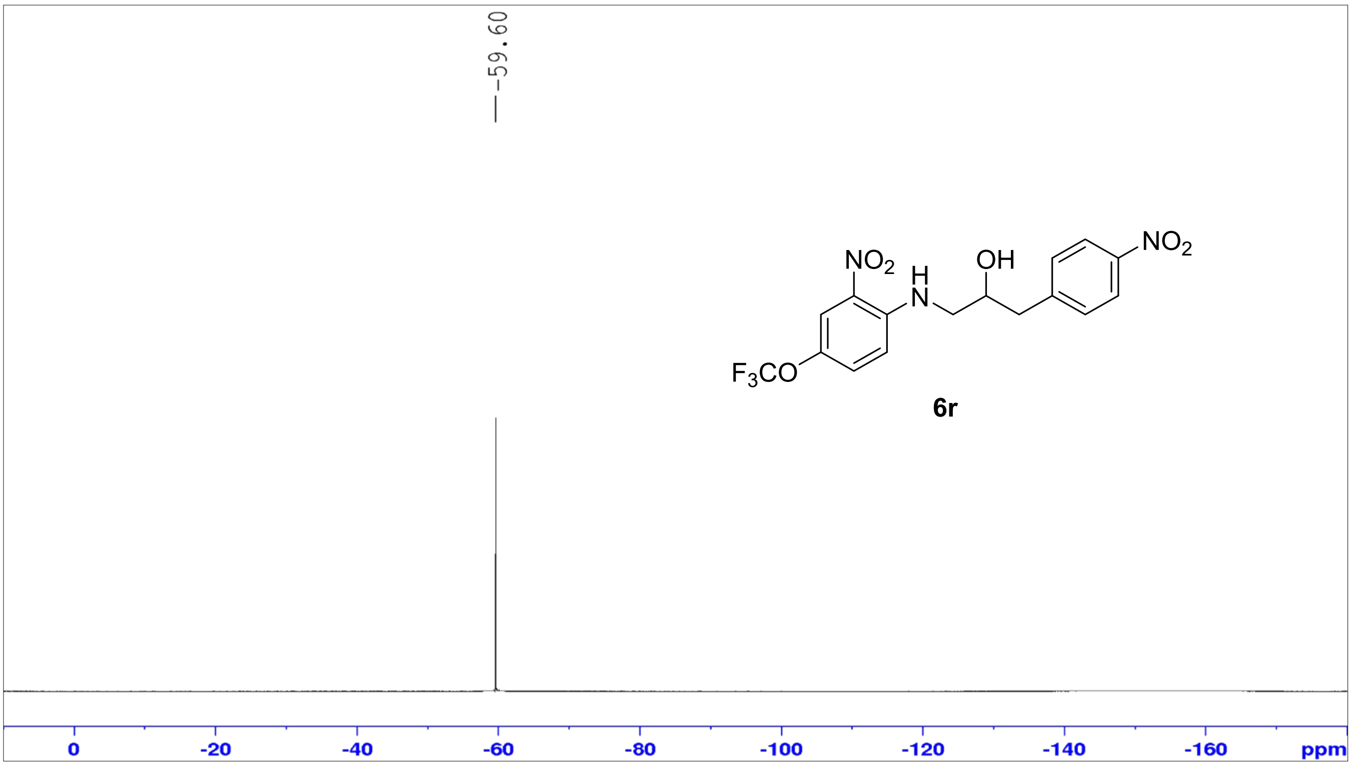

^{19}F NMR (376 MHz, CD_3CN) spectrum of compound **6r**.

^{1}H NMR and ^{13}C NMR of 1-(4-chlorophenyl)-3-((4-nitrophenyl)amino)propan-2-ol (8a)

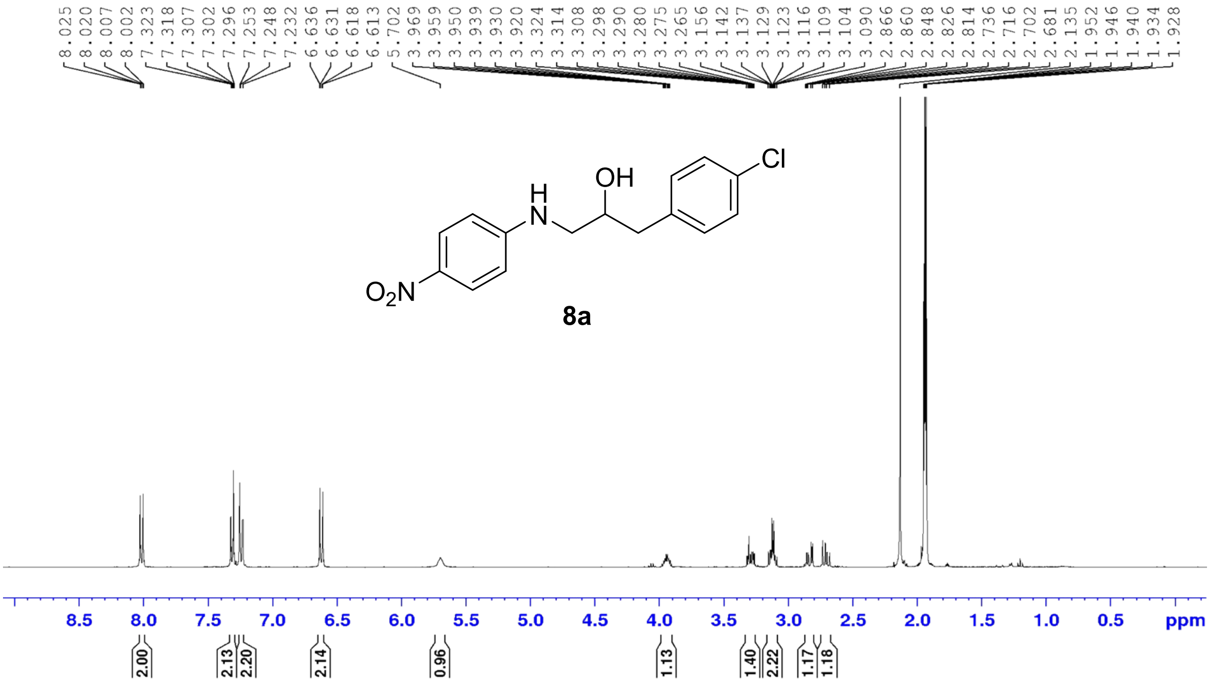

^{1}H NMR (400 MHz, CD_3CN) spectrum of compound **8a**.

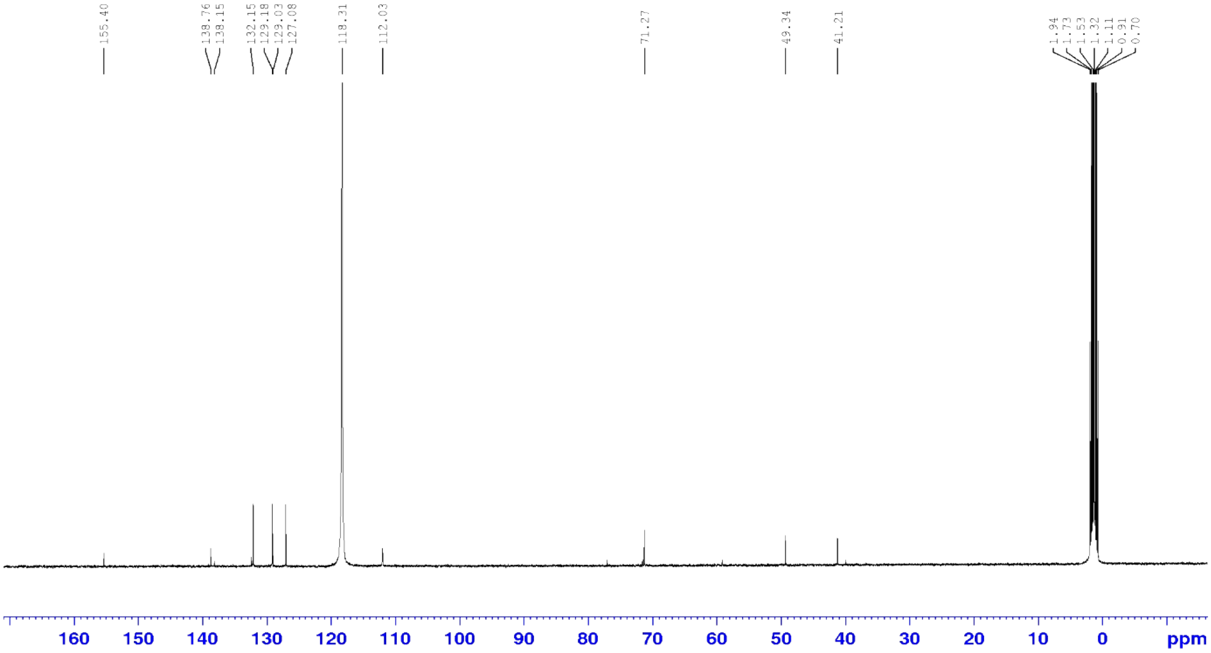

^{13}C NMR (100 MHz, CD_3CN) spectrum of compound **8a**.

^1H NMR and ^{13}C NMR of 1-(4-chlorophenyl)-3-((3-methyl-4-nitrophenyl)amino)propan-2-ol (8b)

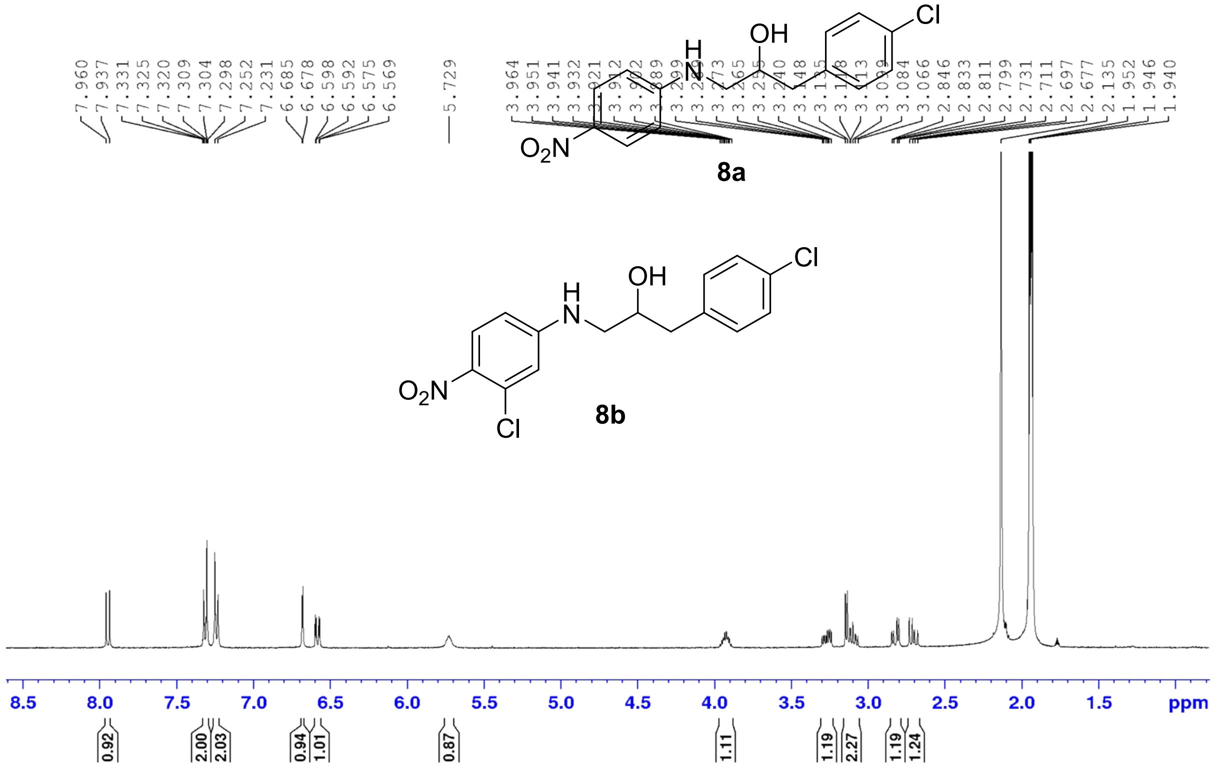

^1H NMR (400 MHz, CD_3CN) spectrum of compound **8b**.

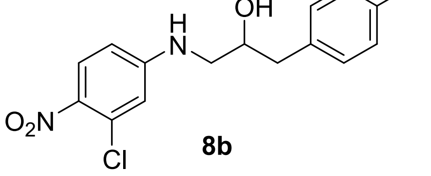

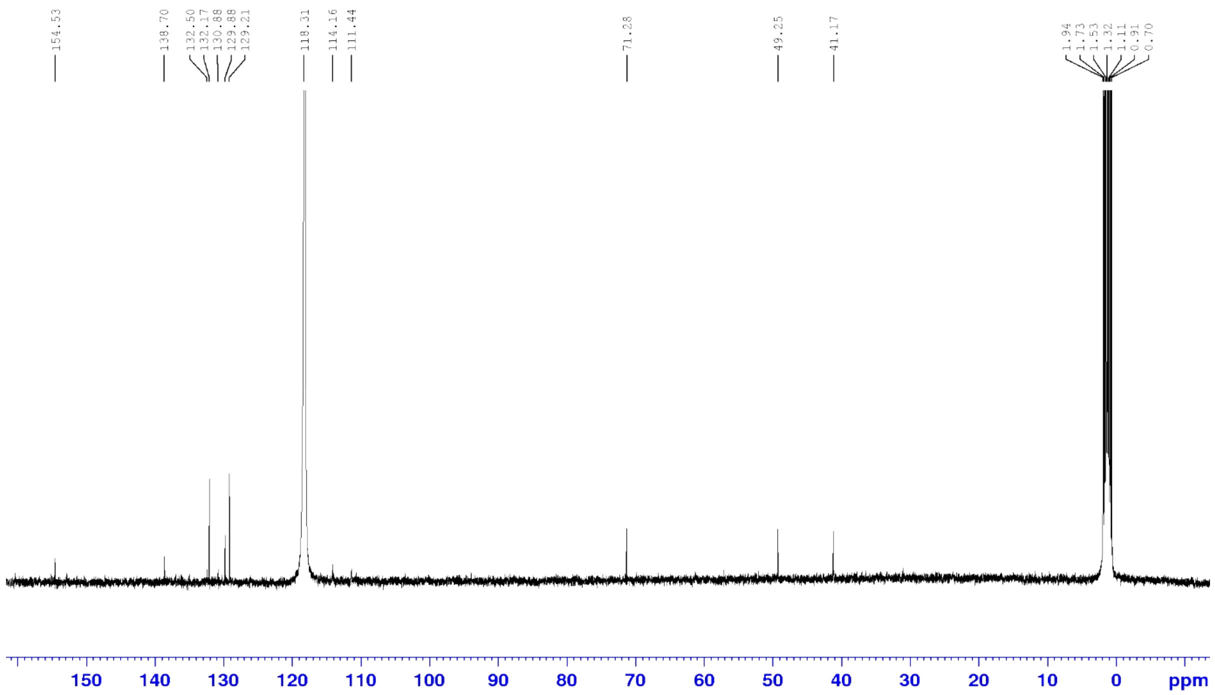

^{13}C NMR (100 MHz, CD_3CN) spectrum of compound **8b**.
